# Supplementary material for: New Cycloadditon Reaction of 2-Chloroprop-2-enethioamides with Dialkyl Acetylenedicarboxylates: Synthesis of Dialkyl 2-[4,5-bis(alkoxycarbonyl)-2-(aryl{alkyl}imino)-3(2H)-thienylidene]-1,3-dithiole-4,5-dicarboxylates
Source: Molecules. 2022 Oct 14;27(20):6887. doi: 10.3390/molecules27206887 (PMC9610143; doi:10.3390/molecules27206887)
Supplement: Supplementary file 1 [file molecules-27-06887-s001.zip › SuppInf-VAO-Mol-8.pdf]

Supplementary Material:

New cycloadditon reaction of 2-chloroprop-2-enethioamides with dialkyl acetylenedicarboxylates: synthesis of dialkyl 2-[4,5-bis(alkoxycarbonyl)-2-(aryl{alkyl}imino)-3(2*H*)-thienylidene]-1,3-dithiole-4,5-dicarboxylates

Vladimir A. Ogurtsov, and Oleg A. Rakitin*

N. D. Zelinsky Institute of Organic Chemistry, Russian Academy of Sciences, 119991 Moscow, Russia

***** Correspondence: orakitin@ioc.ac.ru; Tel.: +7 499 135 5327

Figure S1. ^{1}H NMR spectrum of dimethyl 2-(1-chloro-2-(phenylamino)-2-thioxoethylidene)-1,3-dithiole-4,5-dicarboxylate (**9a**) in $CDCl_3$.

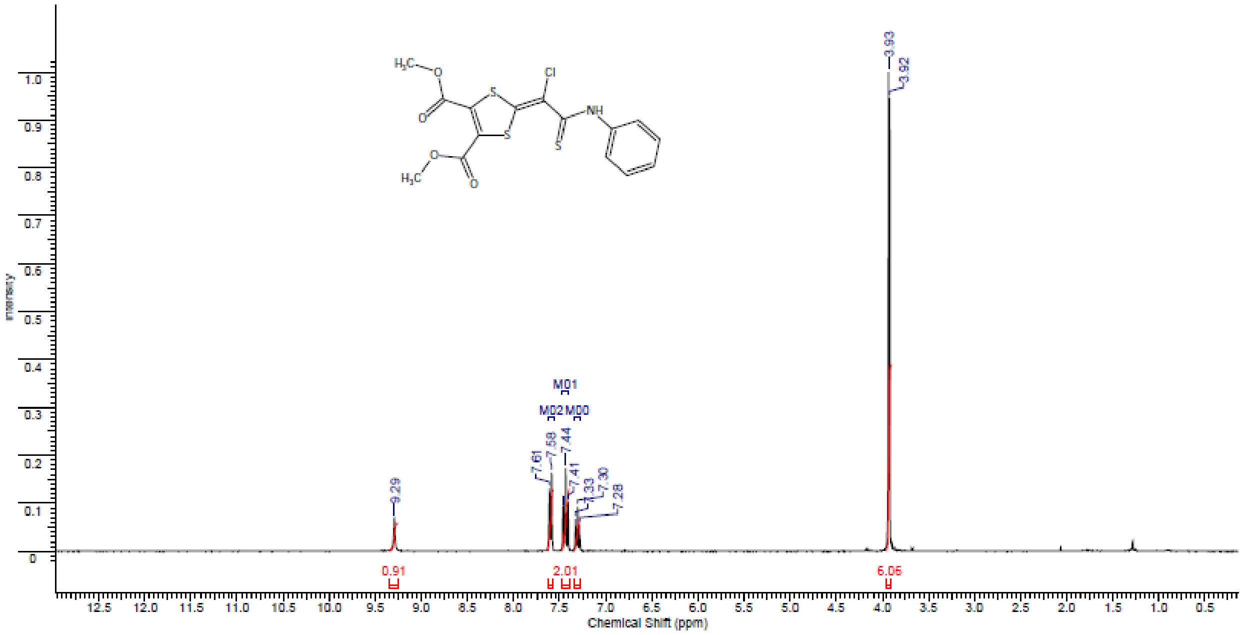

Figure S2. **^{13}C NMR** spectrum of dimethyl 2-(1-chloro-2-(phenylamino)-2-thioxoethylidene)-1,3-dithiole-4,5-dicarboxylate (**9a**) in $CDCl_3$.

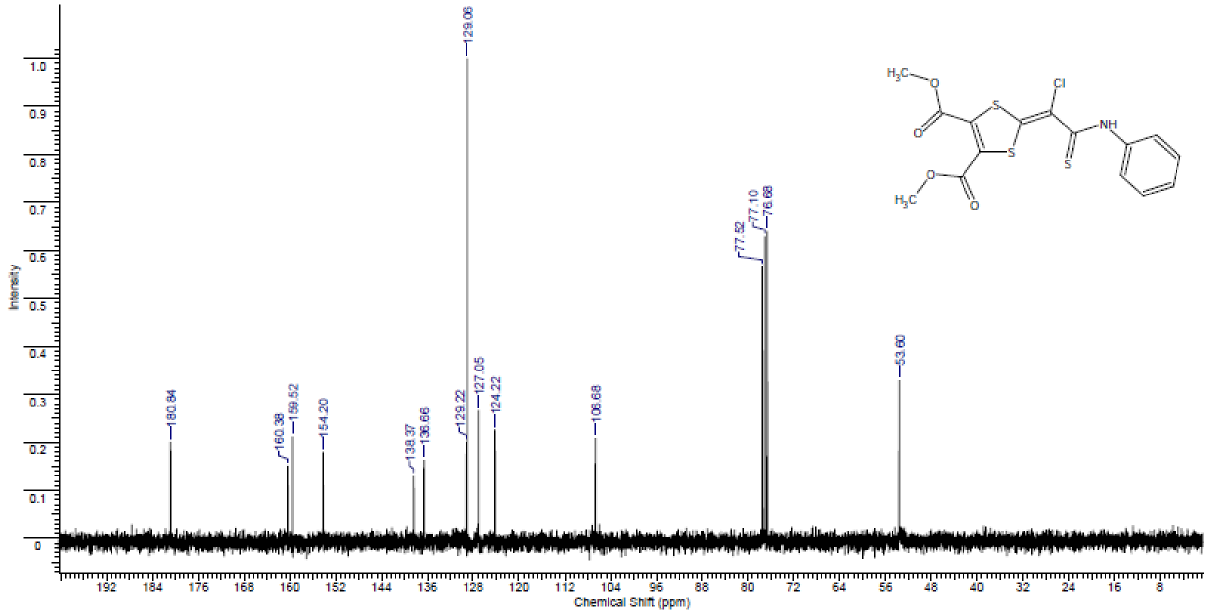

Figure S3. LRMS spectrum of dimethyl 2-(1-chloro-2-(phenylamino)-2-thioxoethylidene)-1,3-dithiole-4,5-dicarboxylate (**9a**).

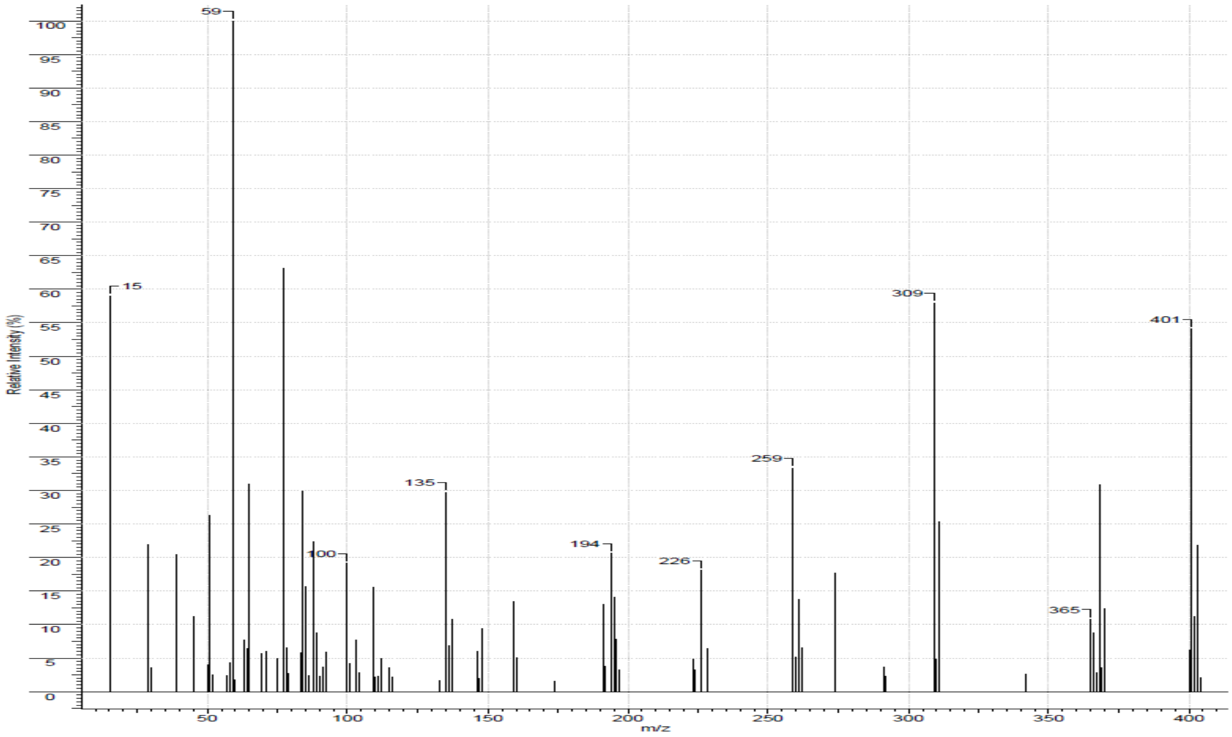

Figure S4. HRMS spectrum of dimethyl 2-(1-chloro-2-(phenylamino)-2-thioxoethylidene)-1,3-dithiole-4,5-dicarboxylate (**9a**).

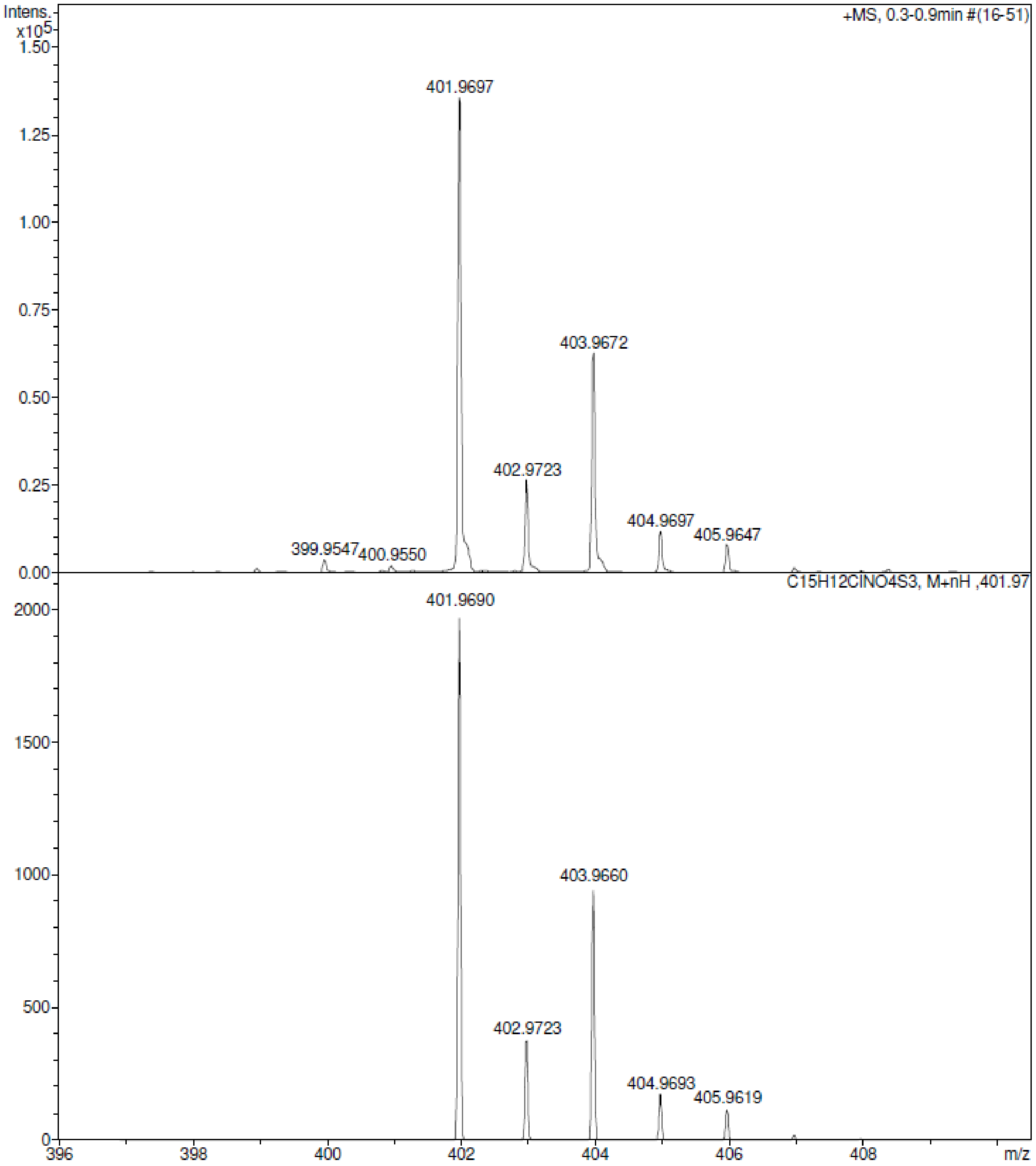

Figure S5. ^{1}H NMR spectrum of dimethyl 2-(1-chloro-2-thioxo-2-(p-tolylamino)ethylidene)-1,3-dithiole-4,5-dicarboxylate (**9b**) in $CDCl_3$.

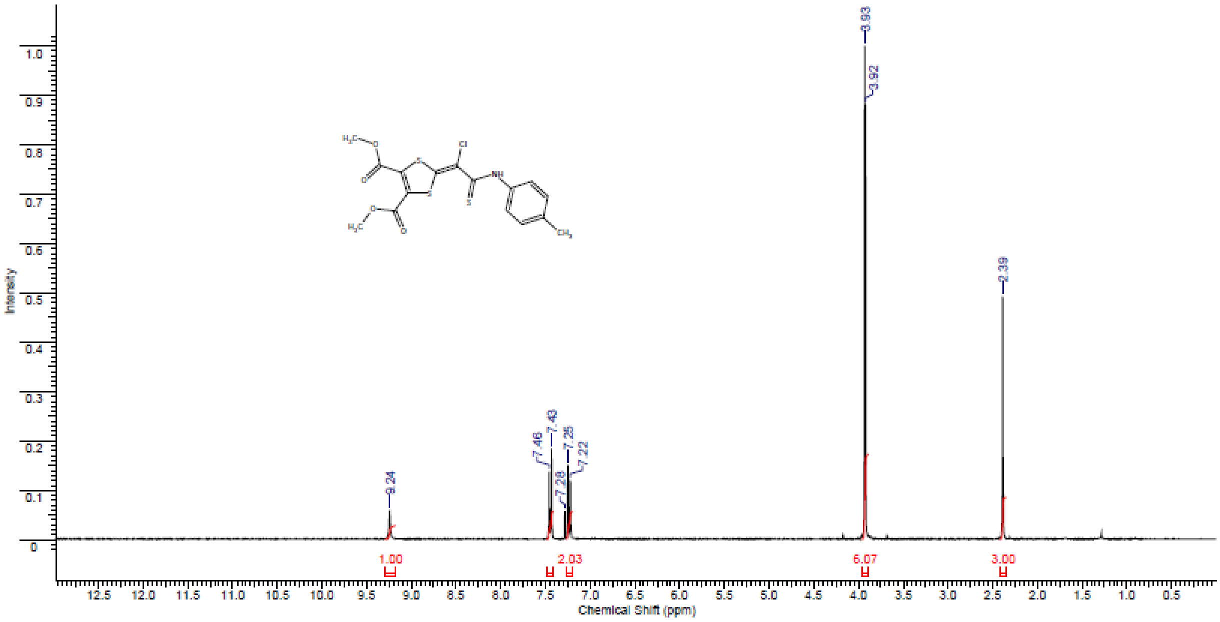

Figure S6. ^{13}C NMR spectrum of dimethyl 2-(1-chloro-2-thioxo-2-(p-tolylamino)ethylidene)-1,3-dithiole-4,5-dicarboxylate (**9b**) in $CDCl_3$.

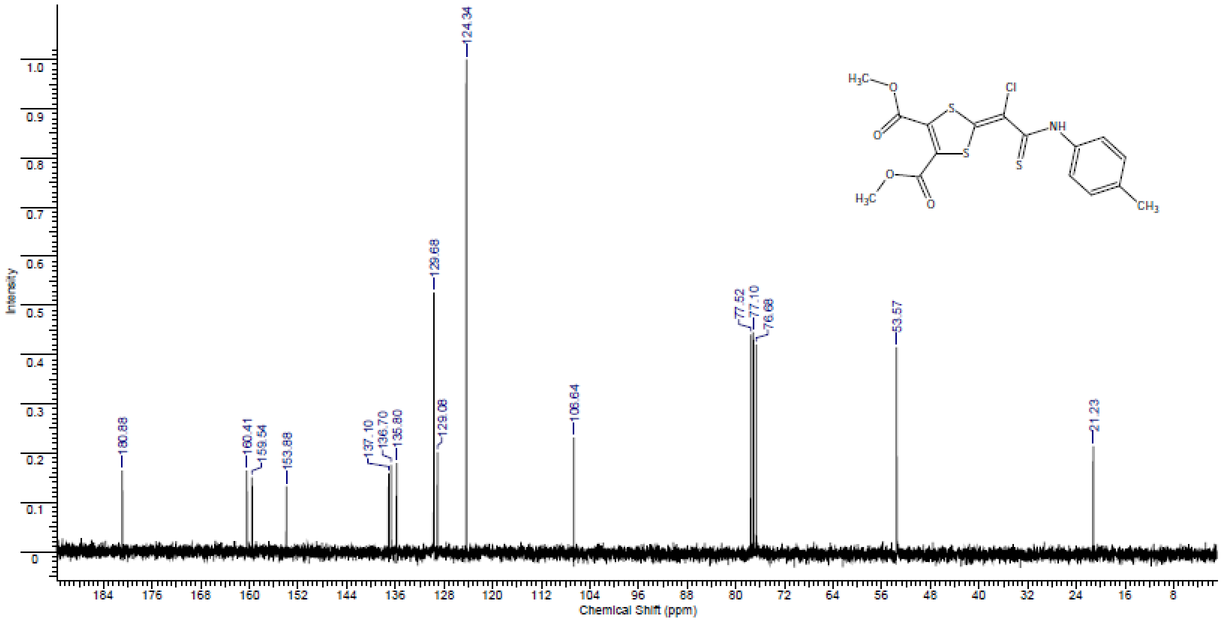

Figure S7. LRMS spectrum of dimethyl 2-(1-chloro-2-thioxo-2-(p-tolylamino)ethylidene)-1,3-dithiole-4,5-dicarboxylate (**9b**).

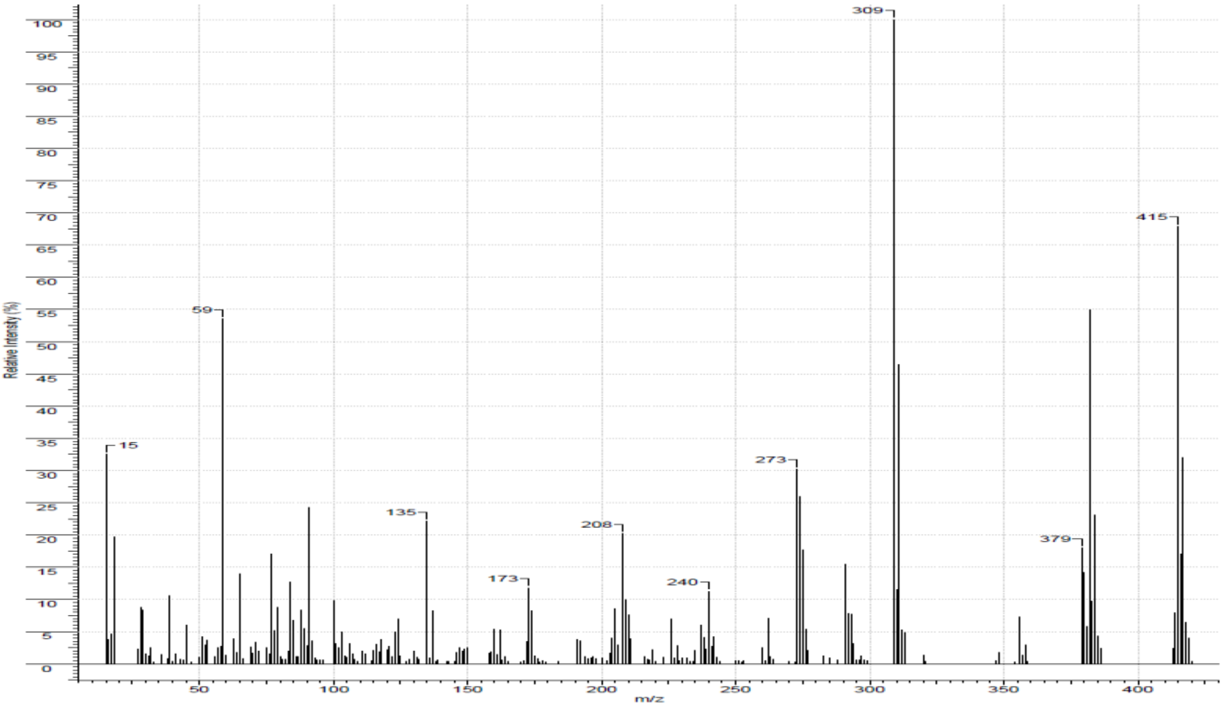

Figure S8. HRMS spectrum of dimethyl 2-(1-chloro-2-thioxo-2-(p-tolylamino)ethylidene)-1,3-dithiole-4,5-dicarboxylate (**9b**).

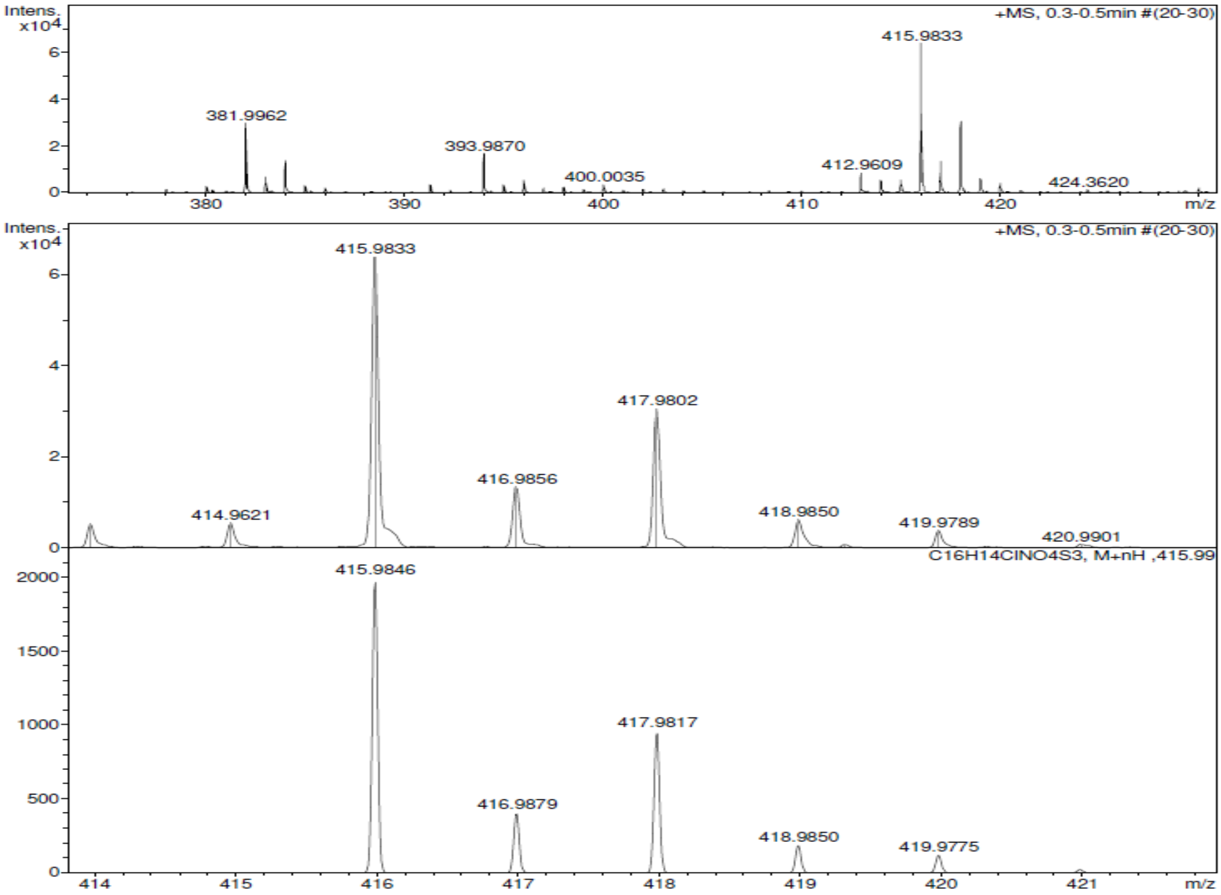

Figure S9. ^{1}H NMR spectrum of dimethyl 2-(1-chloro-2-((4-methoxyphenyl)amino)-2-thioxoethylidene)-1,3-dithiole-4,5-dicarboxylate (**9c**) in $CDCl_3$.

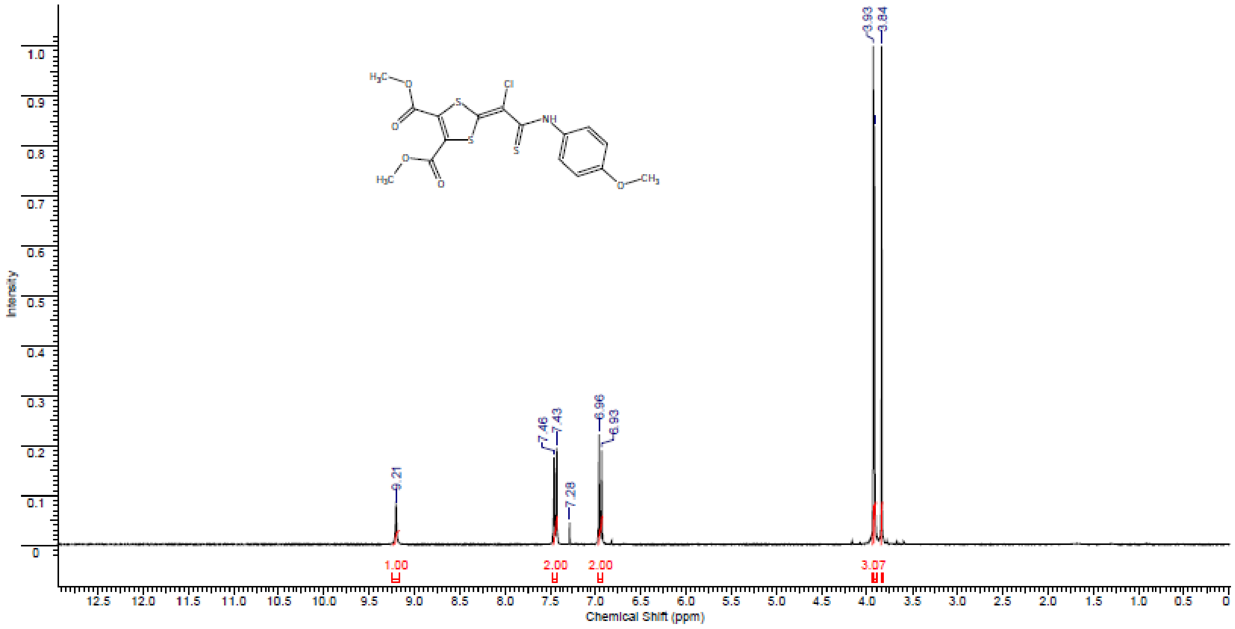

Figure S10. ^{13}C NMR spectrum of dimethyl 2-(1-chloro-2-((4-methoxyphenyl)amino)-2-thioxoethylidene)-1,3-dithiole-4,5-dicarboxylate (**9c**) in $CDCl_3$.

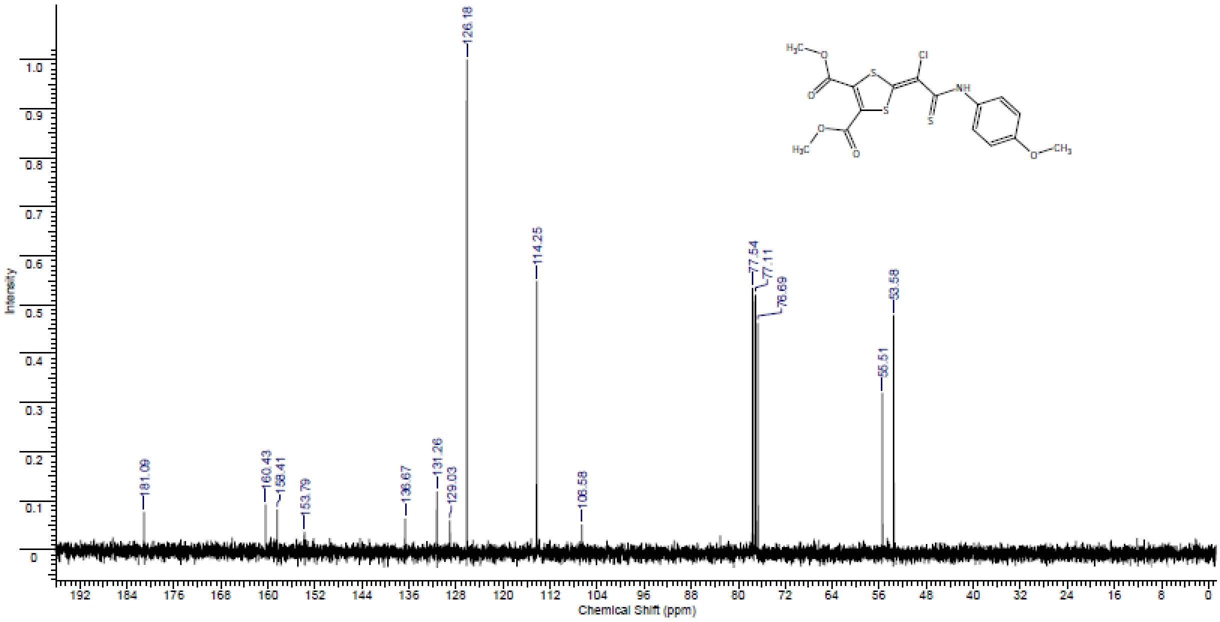

Figure S11. LRMS spectrum of dimethyl2-(1-chloro-2-((4-methoxyphenyl)amino)-2-thioxoethylidene)-1,3-dithiole-4,5-dicarboxylate (**9c**).

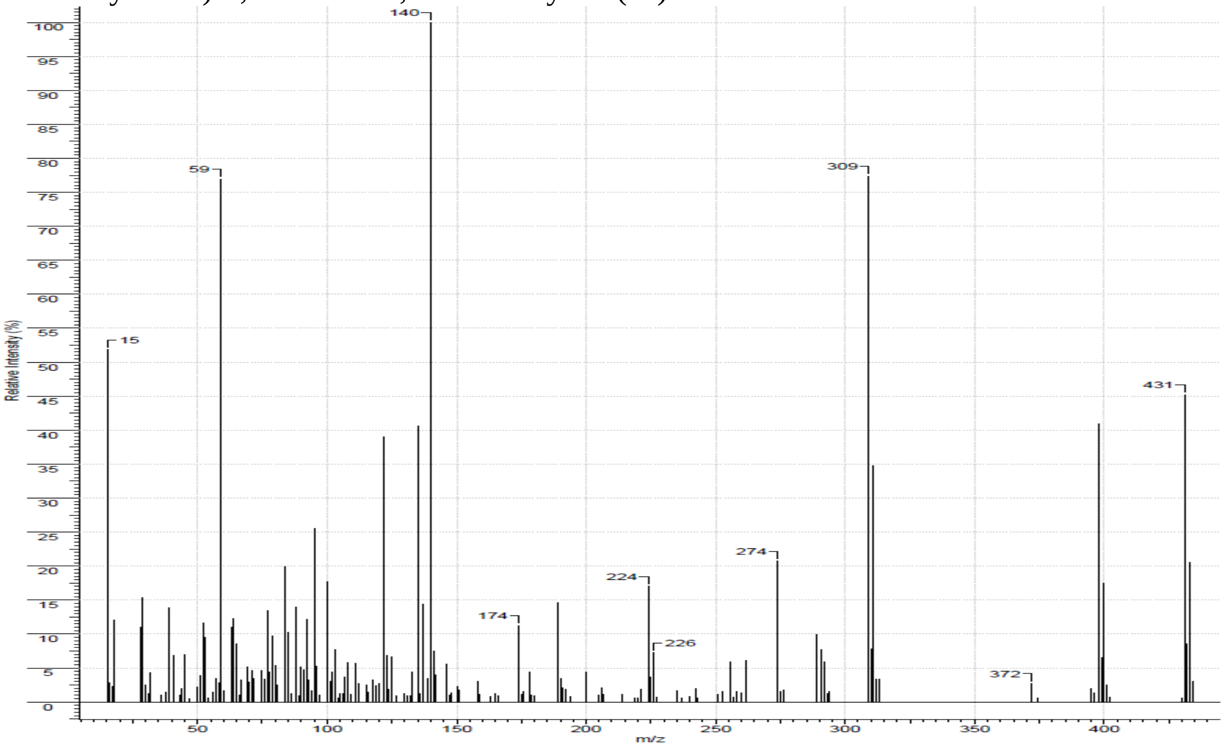

Figure S12. HRMS spectrum of dimethyl2-(1-chloro-2-((4-methoxyphenyl)amino)-2-thioxoethylidene)-1,3-dithiole-4,5-dicarboxylate (**9c**).

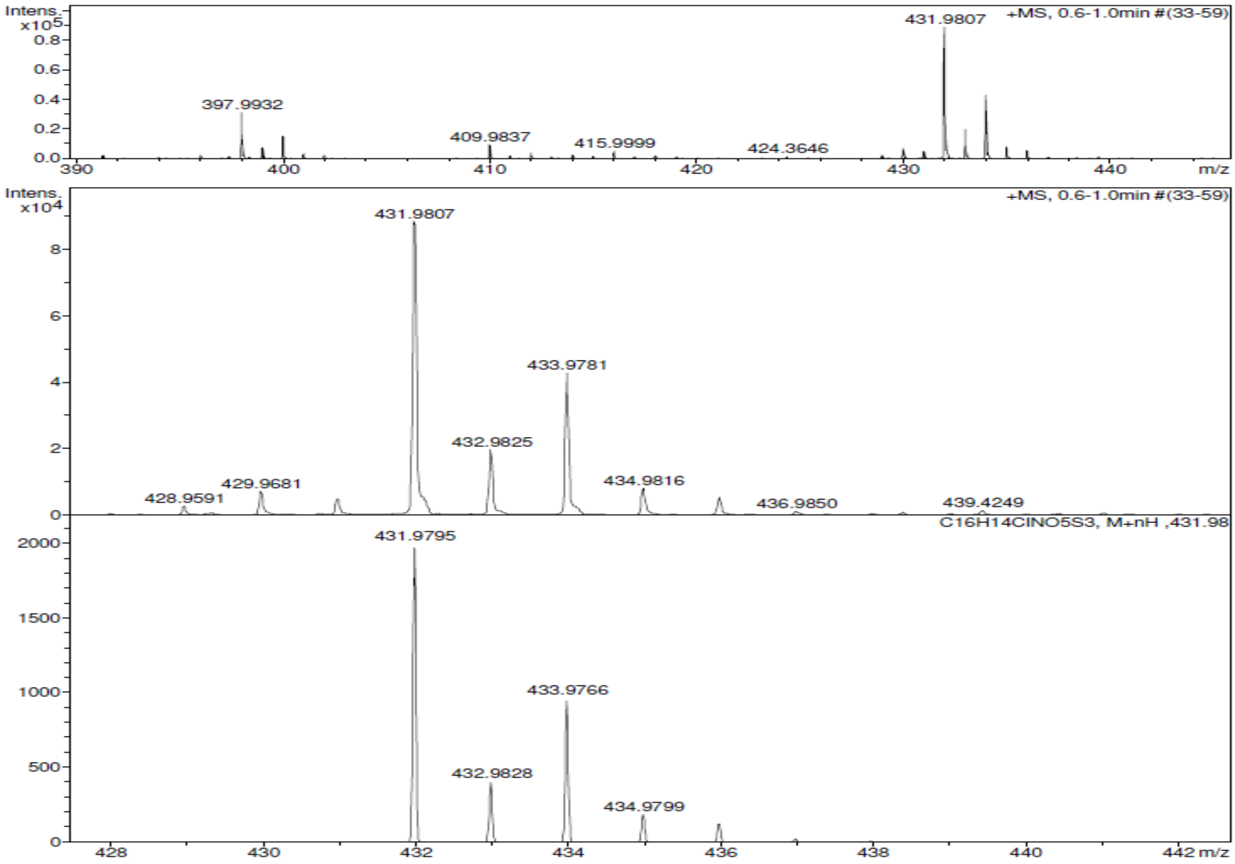

Figure S13. ^{1}H NMR spectrum of dimethyl 2-(1-chloro-2-((4-nitrophenyl)amino)-2-thioxoethylidene)-1,3-dithiole-4,5-dicarboxylate (**9d**) in $CDCl_3$.

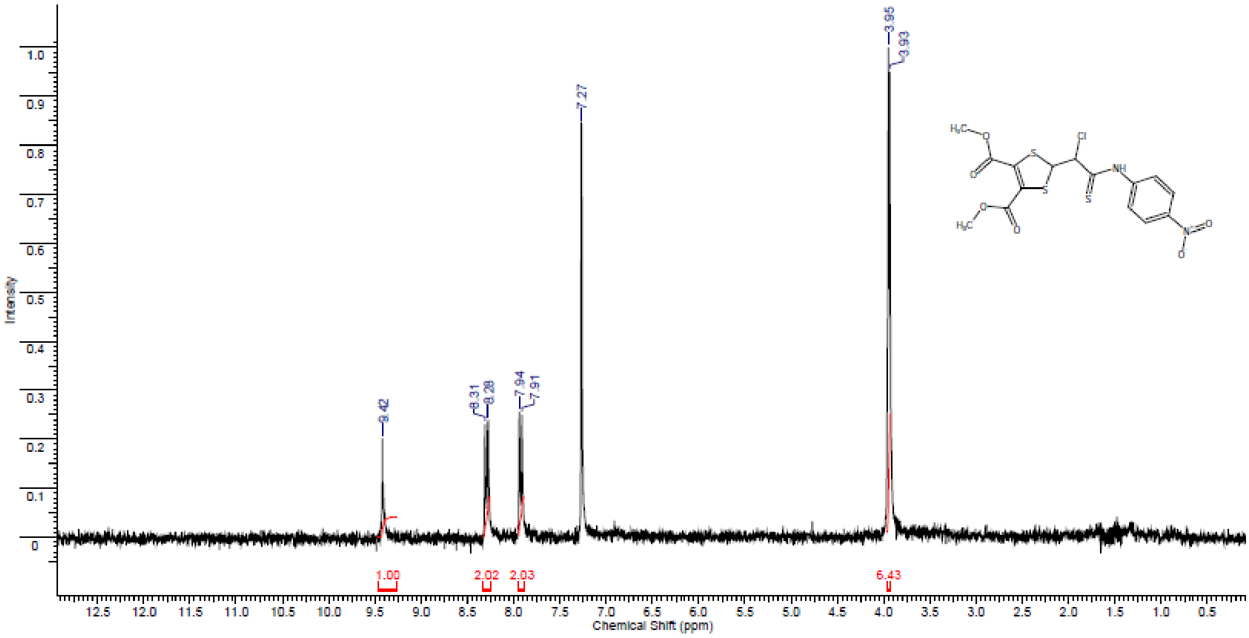

Figure S14. ^{13}C NMR spectrum of dimethyl 2-(1-chloro-2-((4-nitrophenyl)amino)-2-thioxoethylidene)-1,3-dithiole-4,5-dicarboxylate (**9d**) in $CDCl_3$.

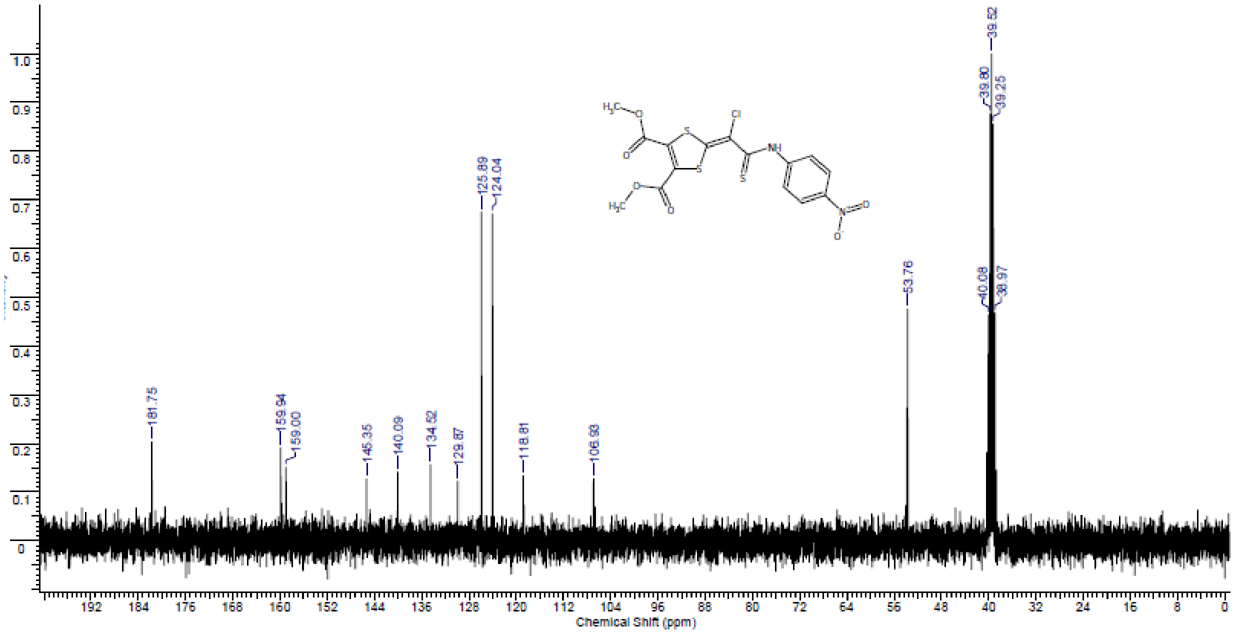

Figure S15. LRMS spectrum of dimethyl 2-(1-chloro-2-((4-nitrophenyl)amino)-2-thioxoethylidene)-1,3-dithiole-4,5-dicarboxylae (**9d**).

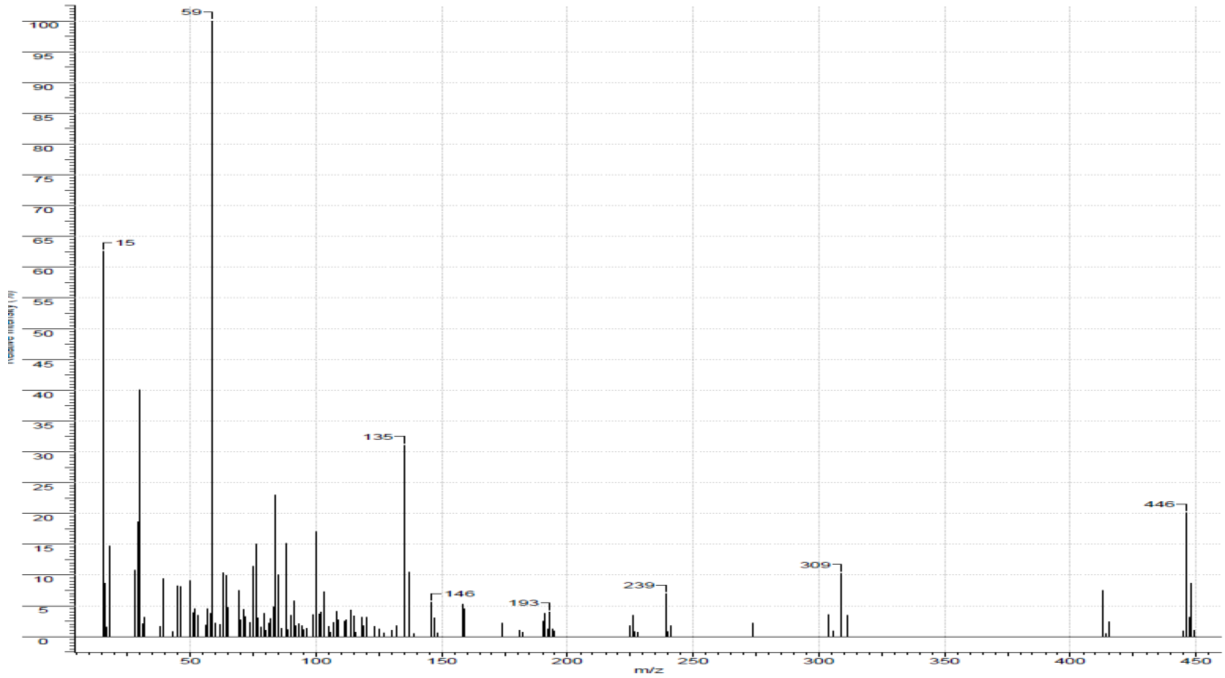

Figure S16. HRMS spectrum of dimethyl 2-(1-chloro-2-((4-nitrophenyl)amino)-2-thioxoethylidene)-1,3-dithiole-4,5-dicarboxylae (**9d**).

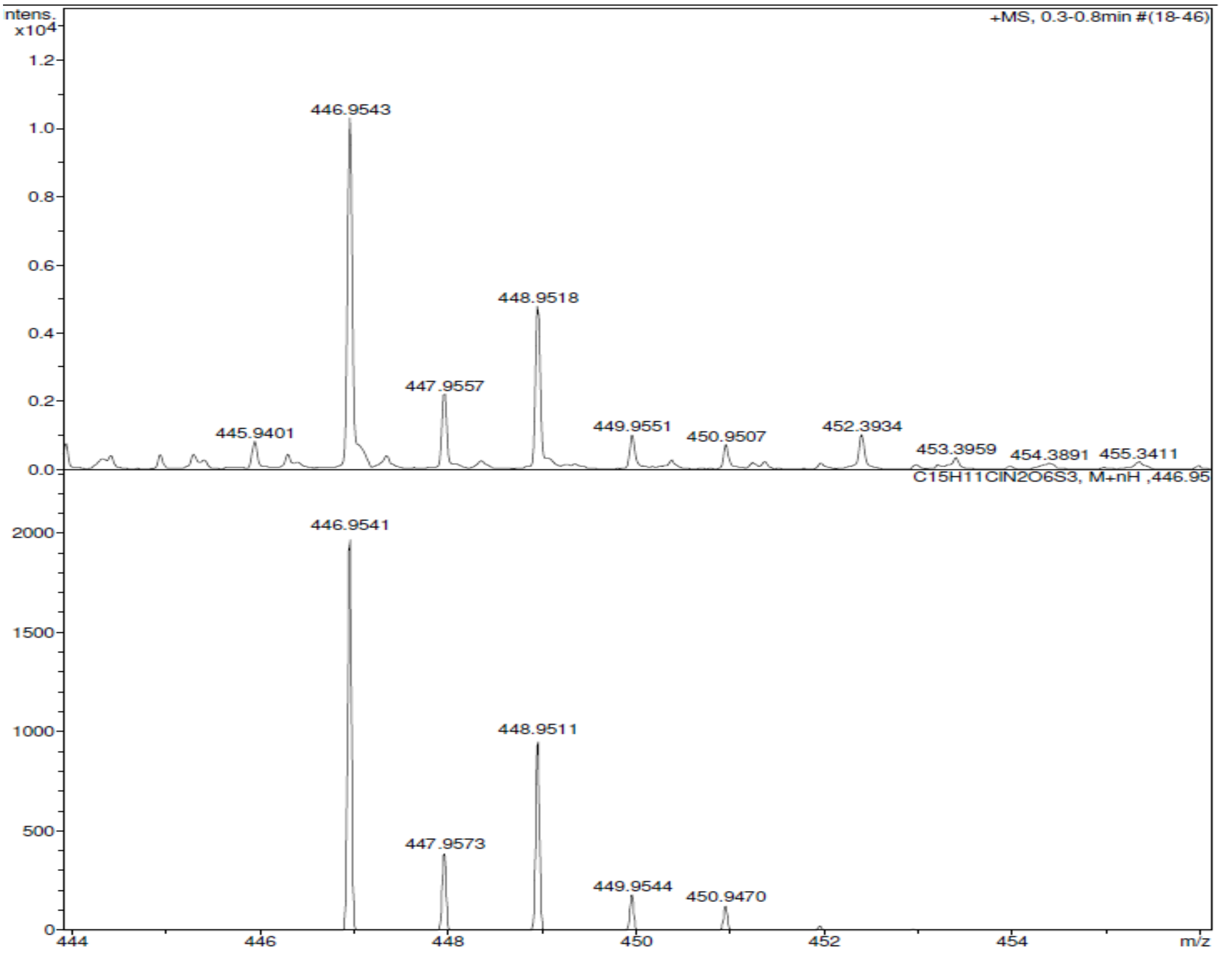

Figure S17. ^{1}H NMR spectrum of dimethyl 2-(1-chloro-2-(methylamino)-2-thioxoethylidene)-1,3-dithiole-4,5-dicarboxylate (**9e**) in $CDCl_3$.

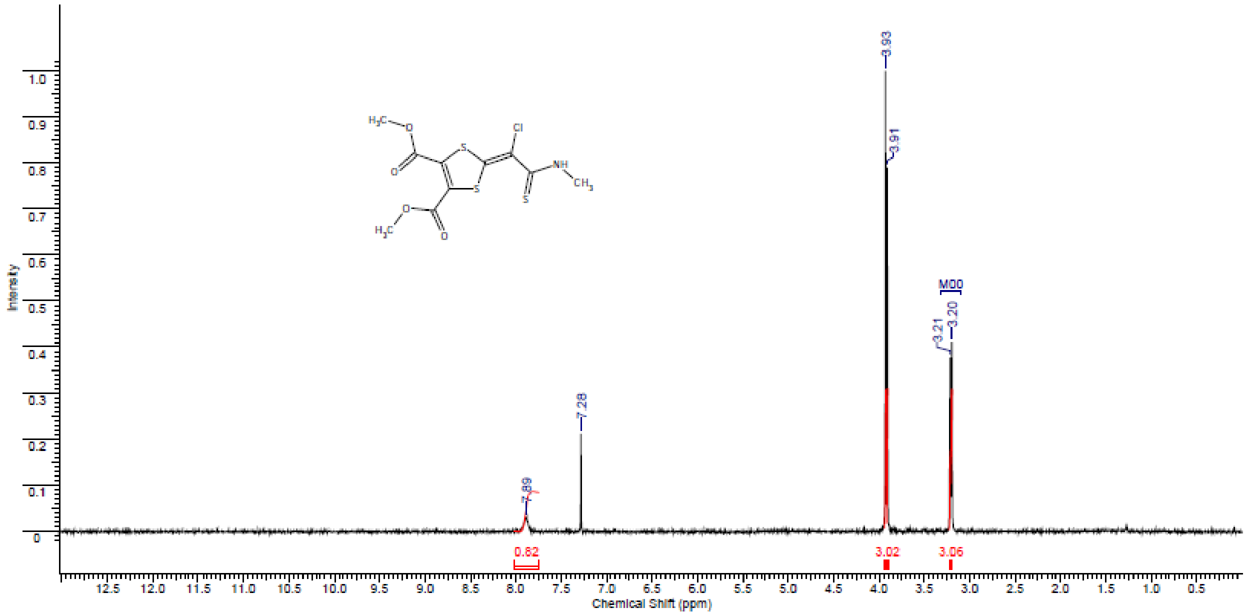

Figure S18. ^{13}C NMR spectrum of dimethyl 2-(1-chloro-2-(methylamino)-2-thioxoethylidene)-1,3-dithiole-4,5-dicarboxylate (**9e**) in $CDCl_3$.

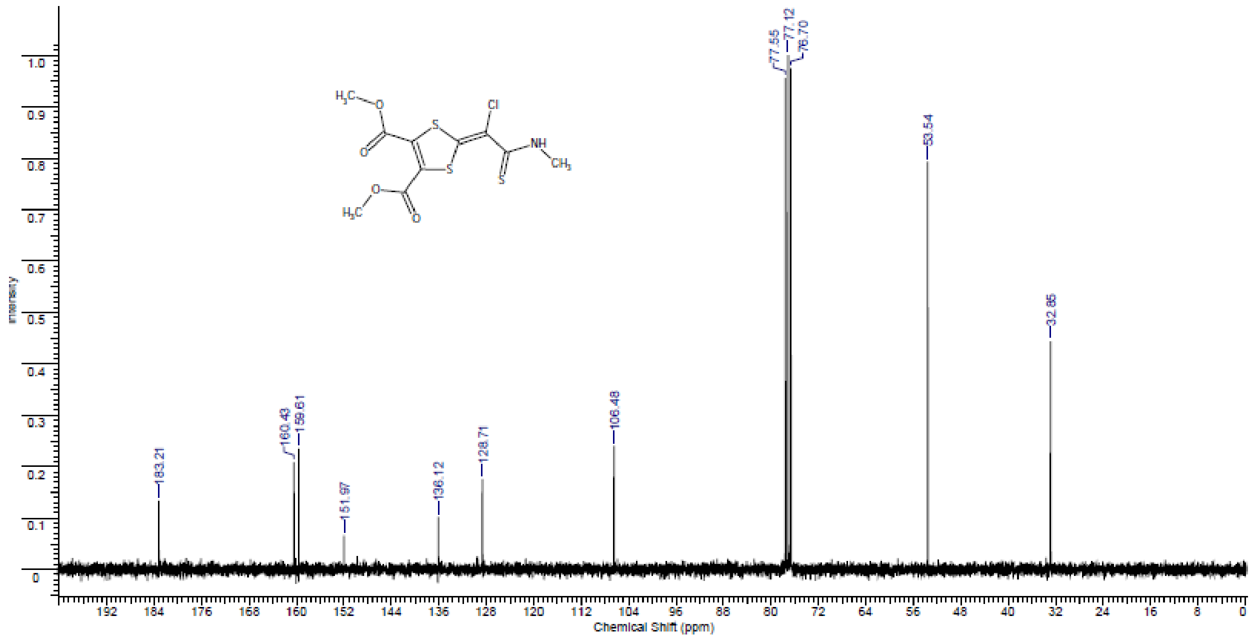

Figure S19. LRMS spectrum of dimethyl2-(1-chloro-2-(methylamino)-2-thioxoethylidene)-1,3-dithiole-4,5-dicarboxylate (**9e**).

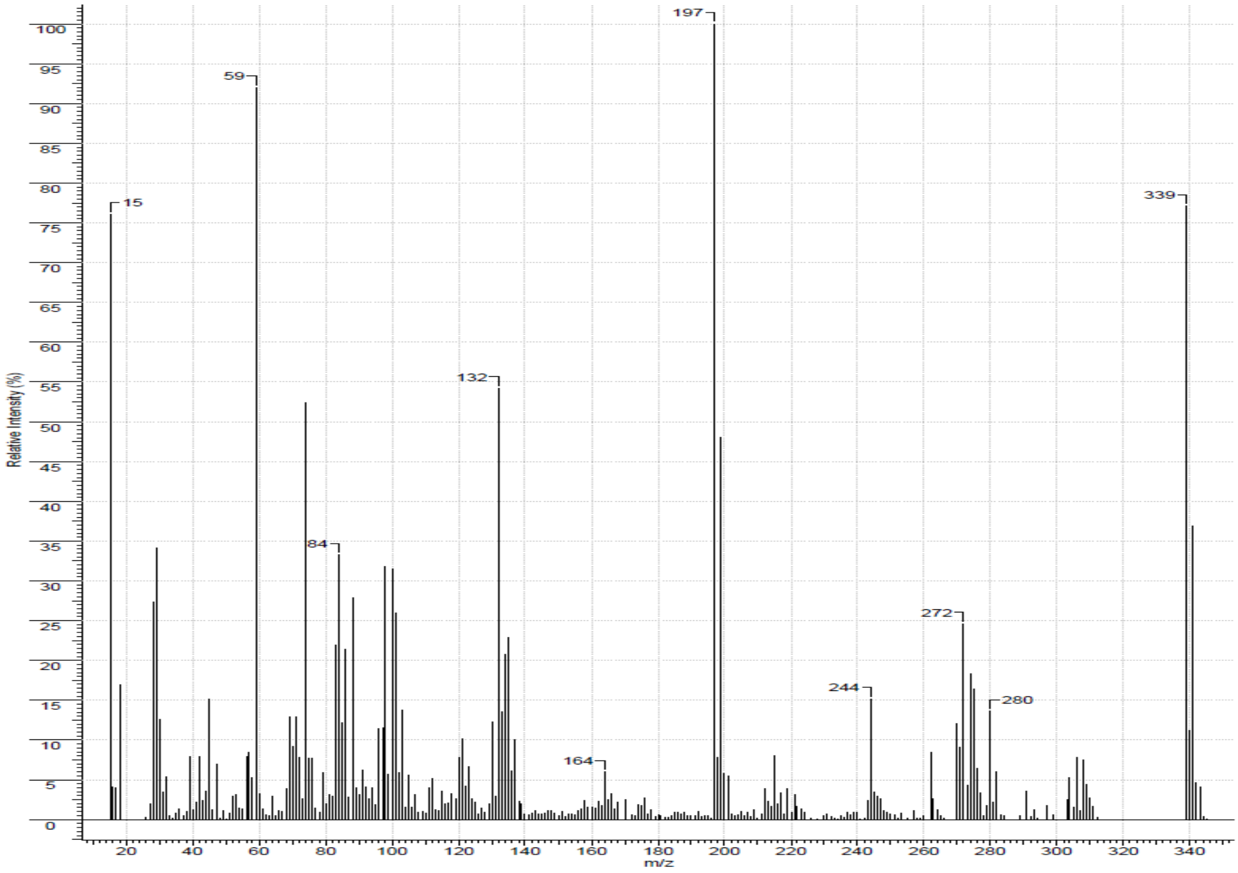

Figure S20. HRMS spectrum of dimethyl2-(1-chloro-2-(methylamino)-2-thioxoethylidene)-1,3-dithiole-4,5-dicarboxylate (**9e**).

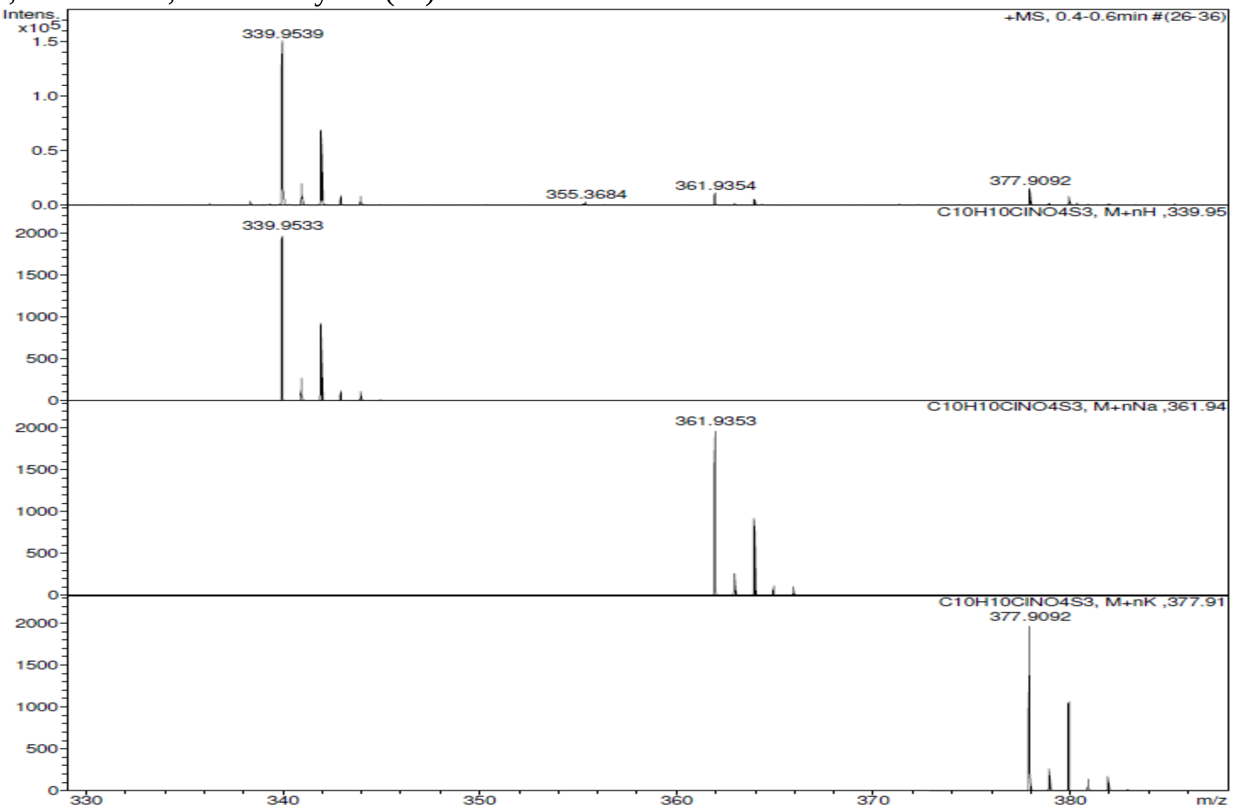

Figure S21. ^{1}H NMR spectrum of dimethyl 2-(2-(tert-butylamino)-1-chloro-2-thioxoethylidene)-1,3-dithiole-4,5-dicarboxylate (**9f**) in $CDCl_3$.

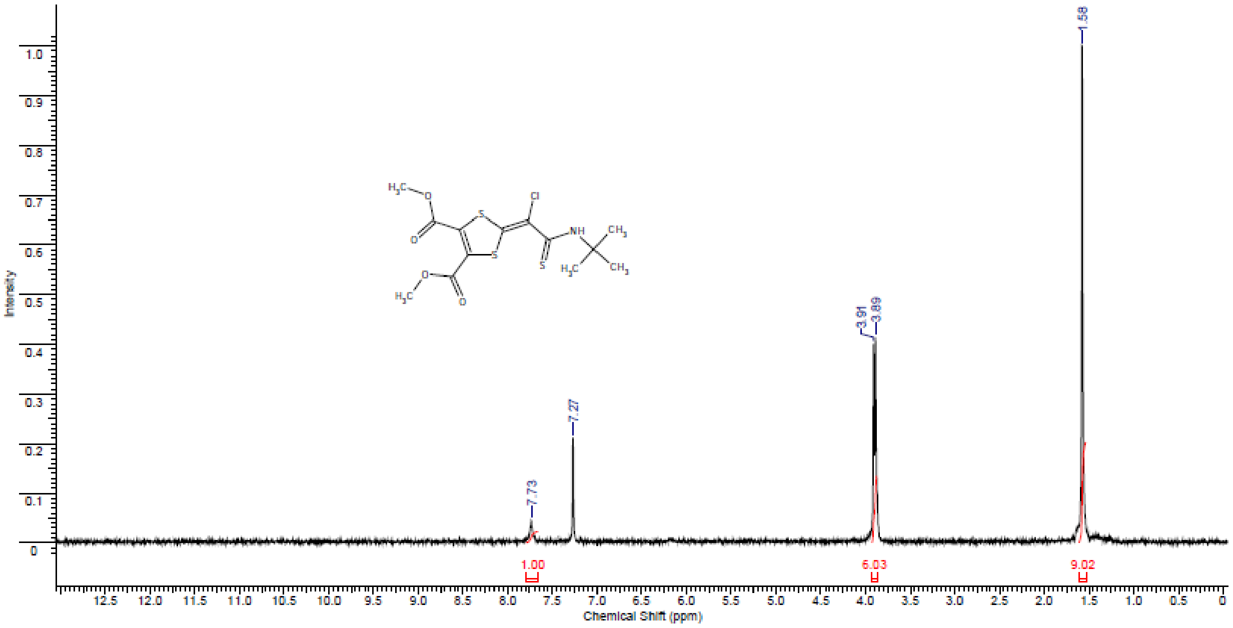

Figure S22. ^{13}C NMR spectrum of dimethyl 2-(2-(tert-butylamino)-1-chloro-2-thioxoethylidene)-1,3-dithiole-4,5-dicarboxylate (**9f**) in $CDCl_3$.

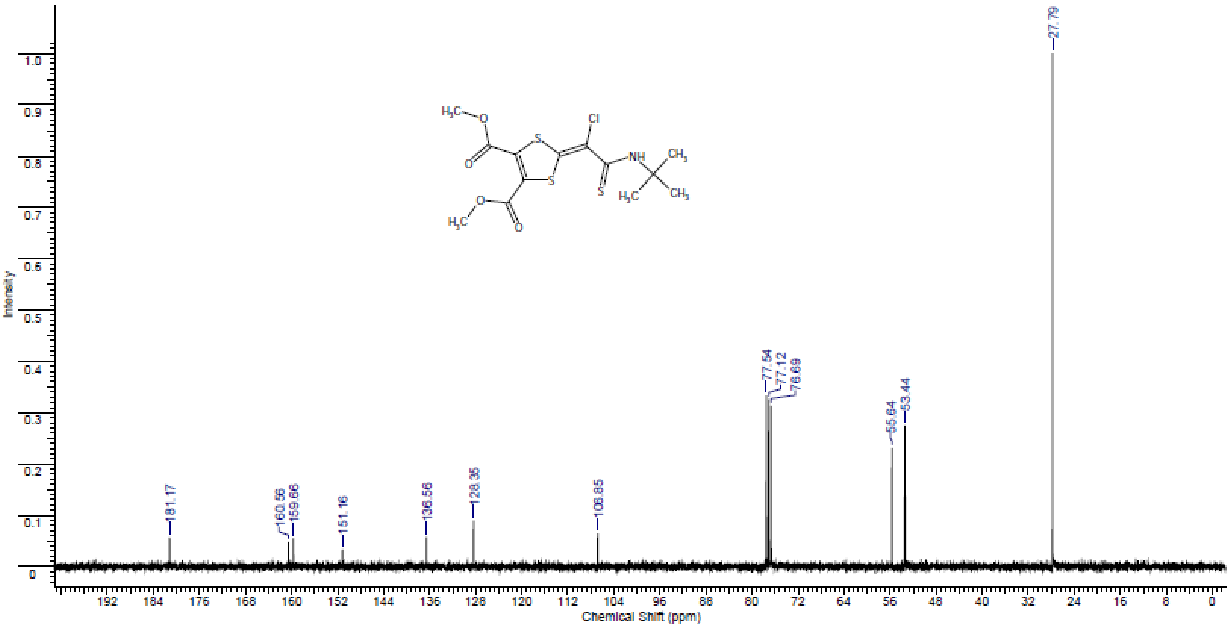

Figure S23. LRMS spectrum of dimethyl2-(2-(tert-butylamino)-1-chloro-2-thioxoethylidene)-1,3-dithiole-4,5-dicarboxylate (**9f**).

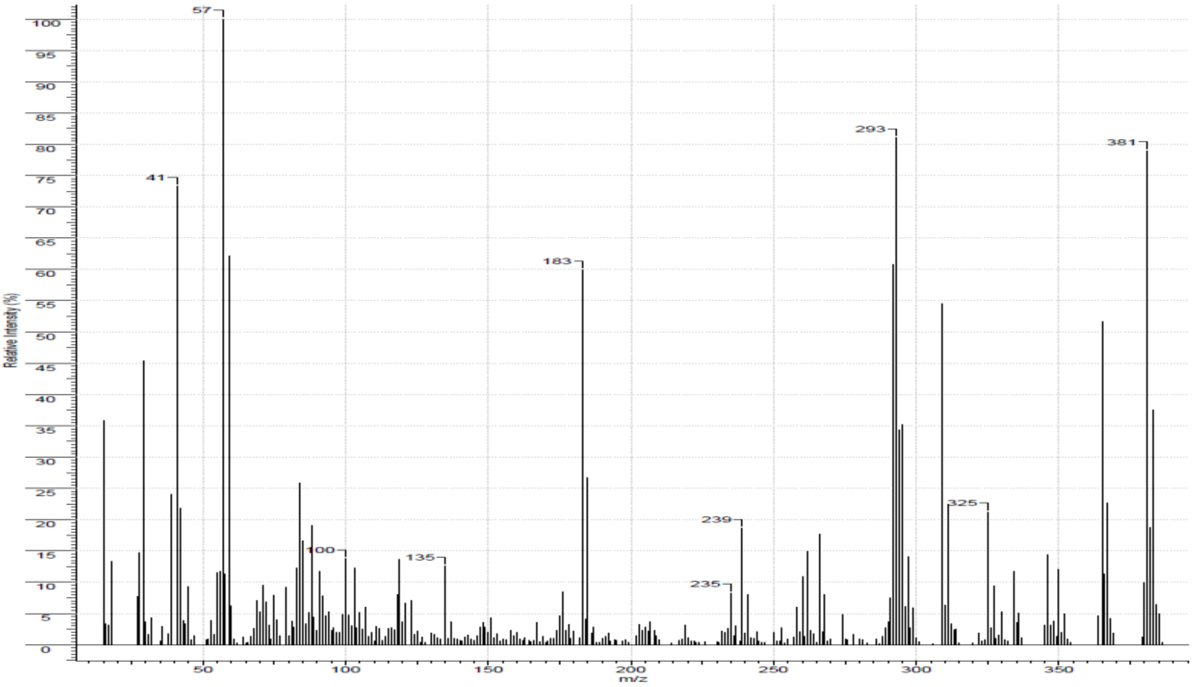

Figure S24. HRMS spectrum of dimethyl2-(2-(tert-butylamino)-1-chloro-2-thioxoethylidene)-1,3-dithiole-4,5-dicarboxylate (**9f**).

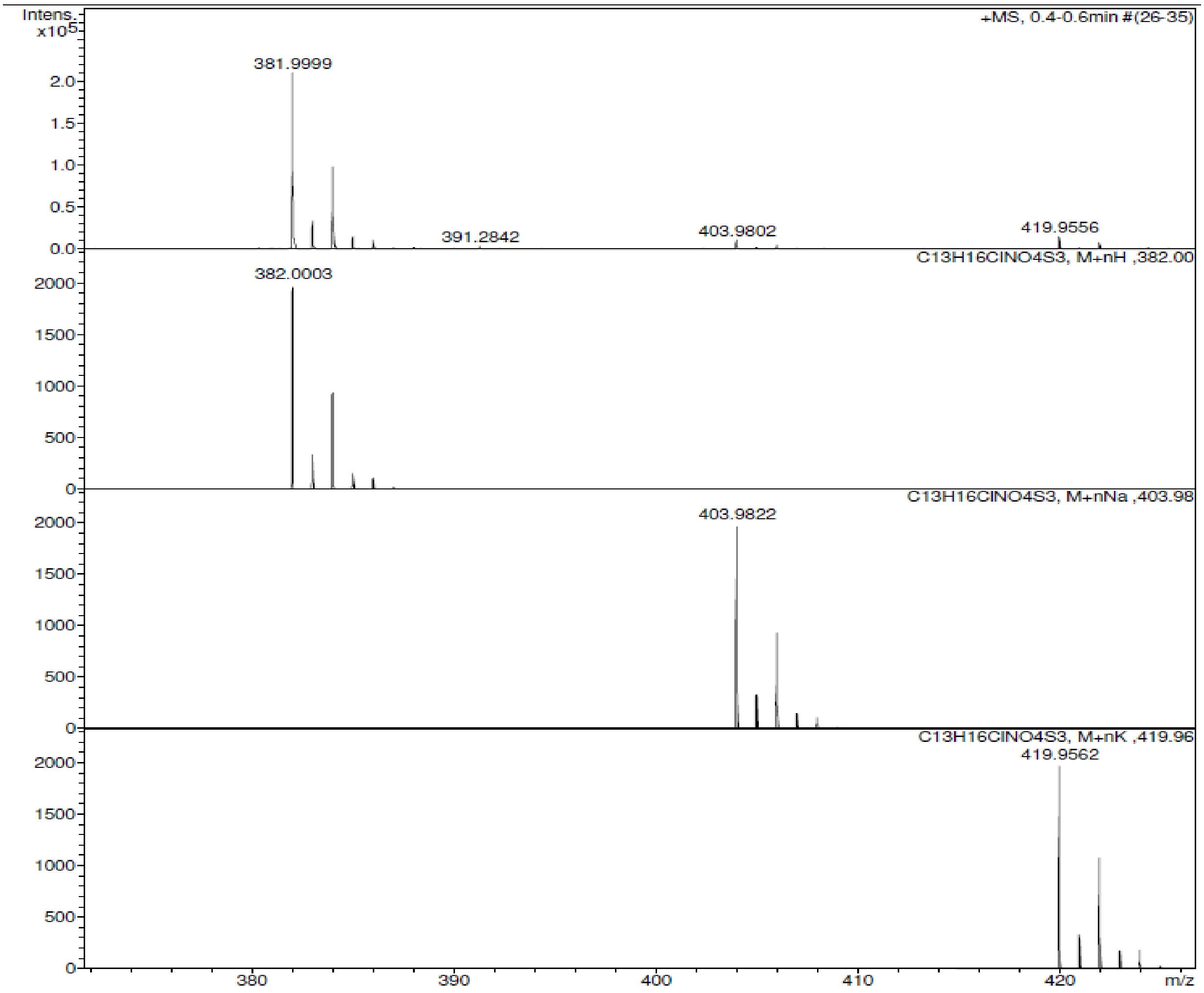

Figure S25. ^{1}H NMR spectrum of dimethyl 2-(1-chloro-2-(dimethylamino)-2-thioxoethylidene)-1,3-dithiole-4,5-dicarboxylate (**9g**) in $CDCl_3$.

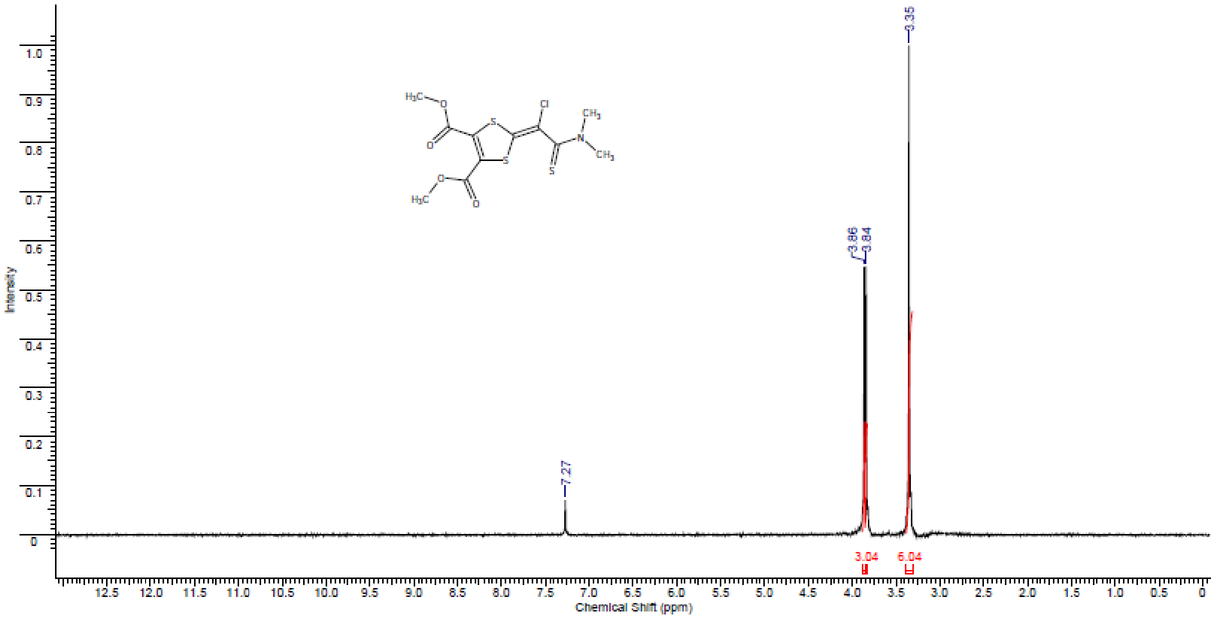

Figure S26. ^{13}C NMR spectrum of dimethyl 2-(1-chloro-2-(dimethylamino)-2-thioxoethylidene)-1,3-dithiole-4,5-dicarboxylate (**9g**) in $CDCl_3$.

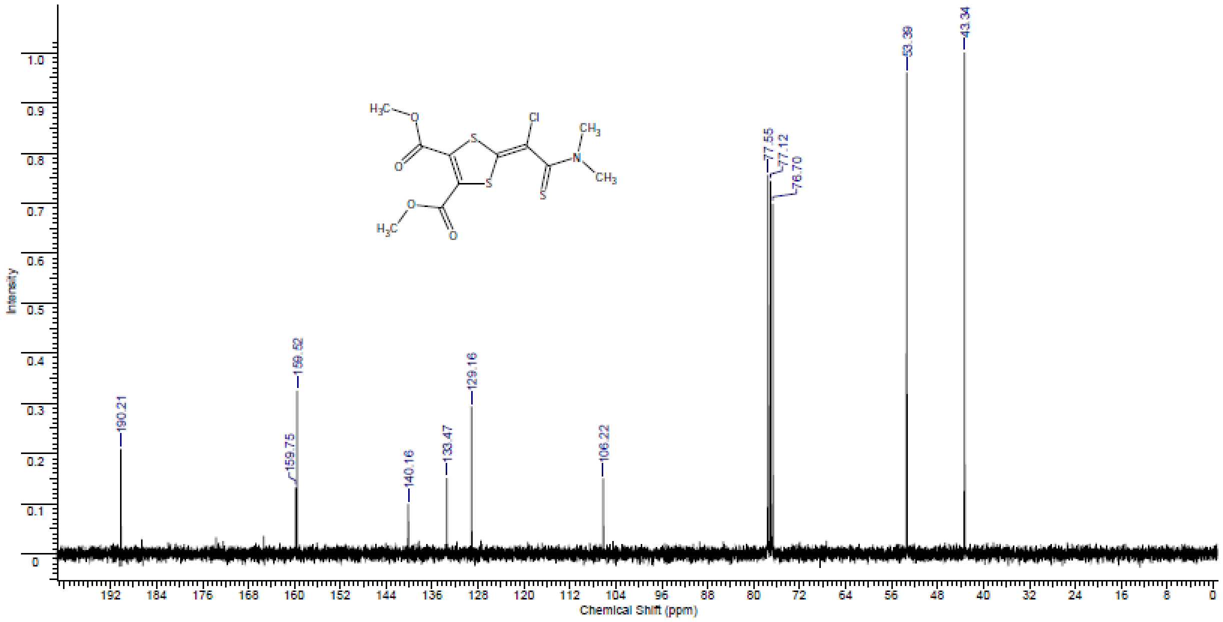

Figure S27. LRMS spectrum of dimethyl2-(1-chloro-2-(dimethylamino)-2-thioxoethylidene)-1,3-dithiole-4,5-dicarboxylate (**9g**).

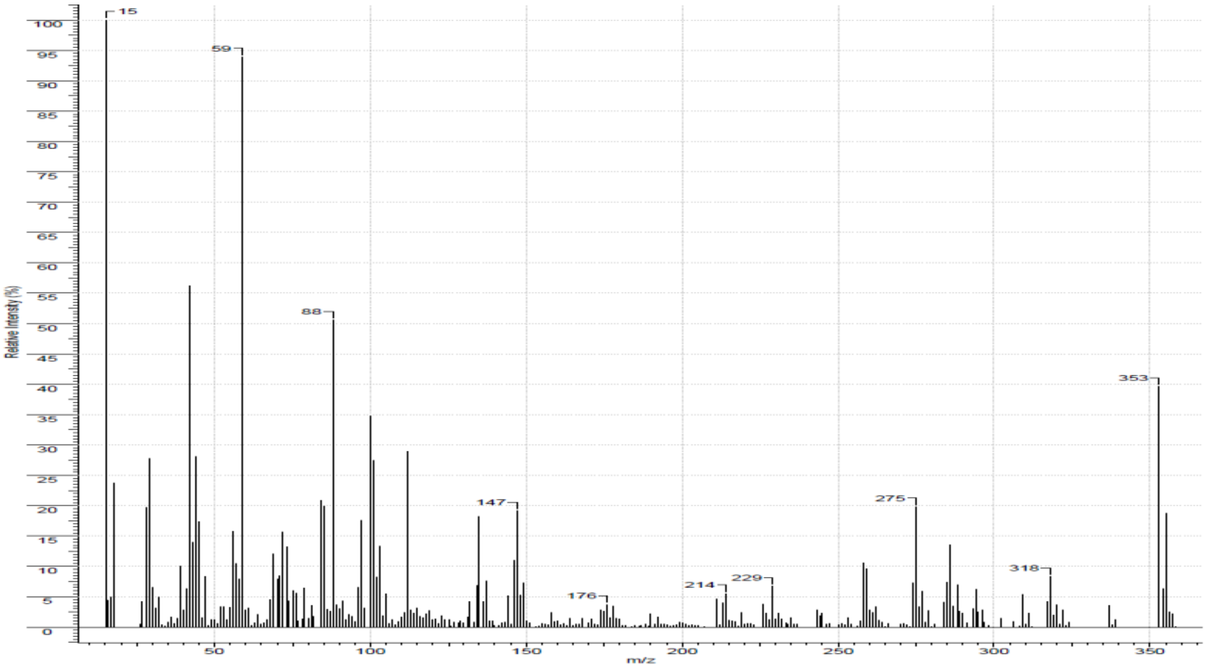

Figure S28. HRMS spectrum of dimethyl2-(1-chloro-2-(dimethylamino)-2-thioxoethylidene)-1,3-dithiole-4,5-dicarboxylate (**9g**).

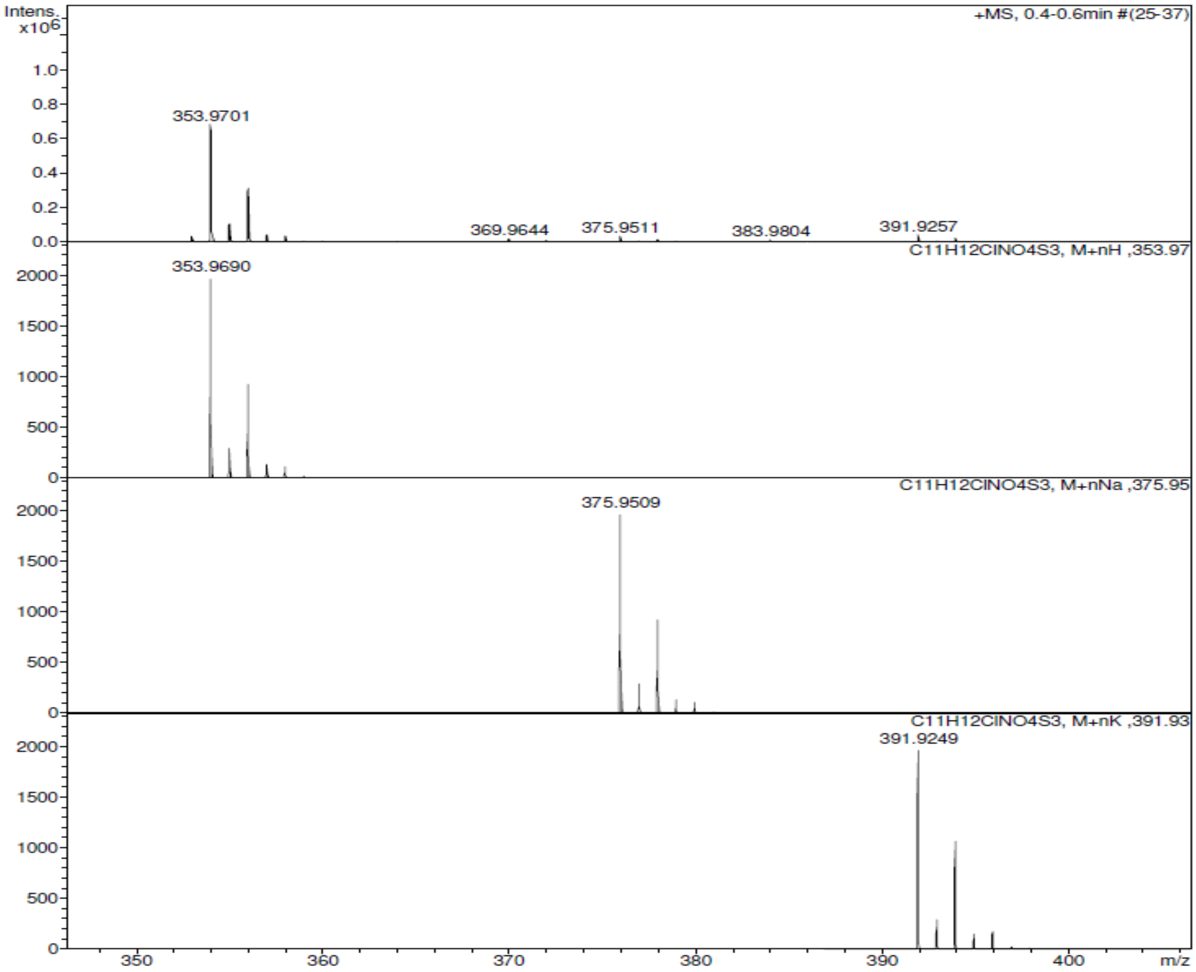

Figure S29. ^{1}H NMR spectrum of diethyl 2-(1-chloro-2-(phenylamino)-2-thioxoethylidene)-1,3-dithiole-4,5-dicarboxylate (**12**) in $CDCl_3$.

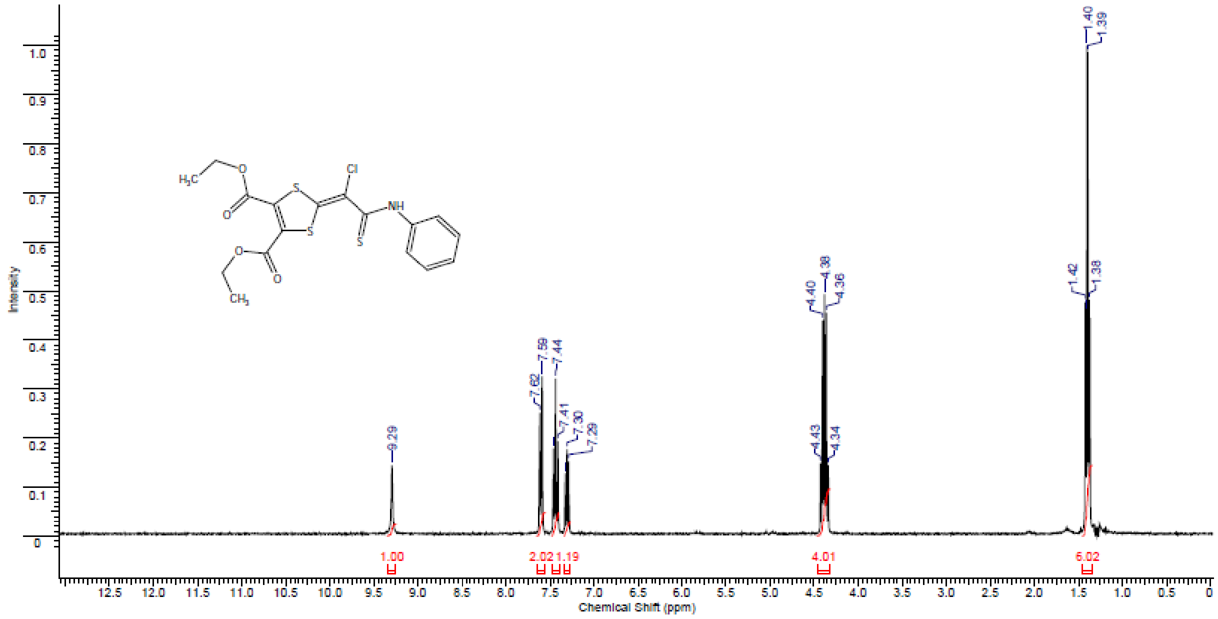

Figure S30. ^{13}C NMR spectrum of diethyl 2-(1-chloro-2-(phenylamino)-2-thioxoethylidene)-1,3-dithiole-4,5-dicarboxylate (**12**) in $CDCl_3$.

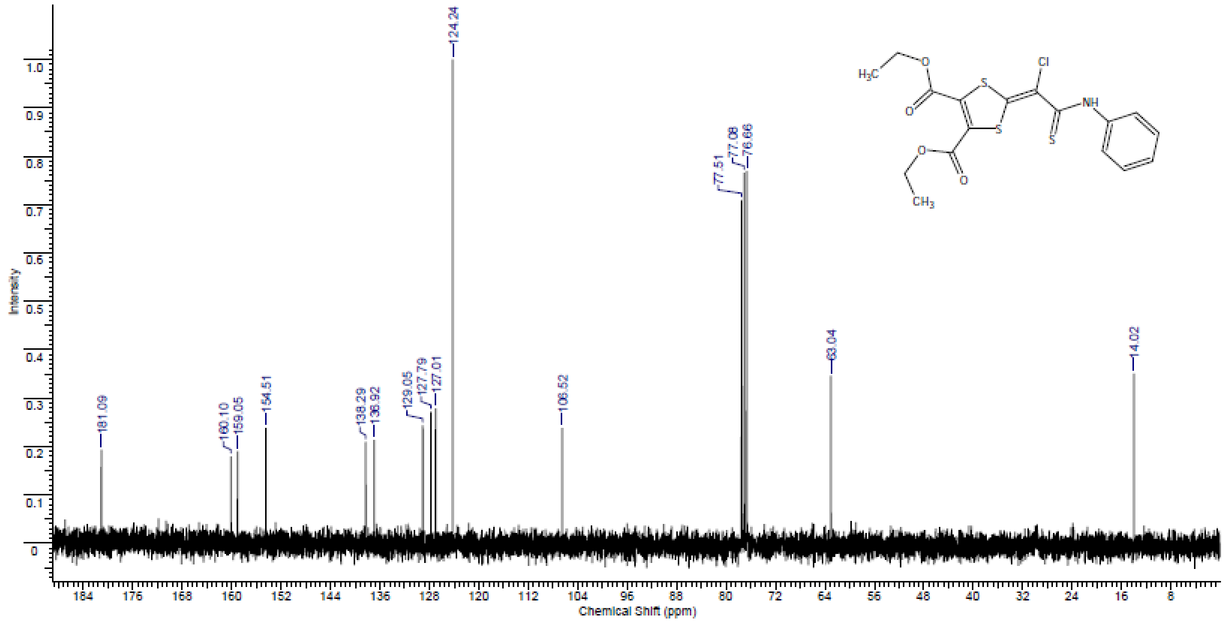

Figure S31. LRMS spectrum of dimethyl2-(1-chloro-2-(phenylamino)-2-thioxoethylidene)-1,3-dithiole-4,5-dicarboxylate (**12**).

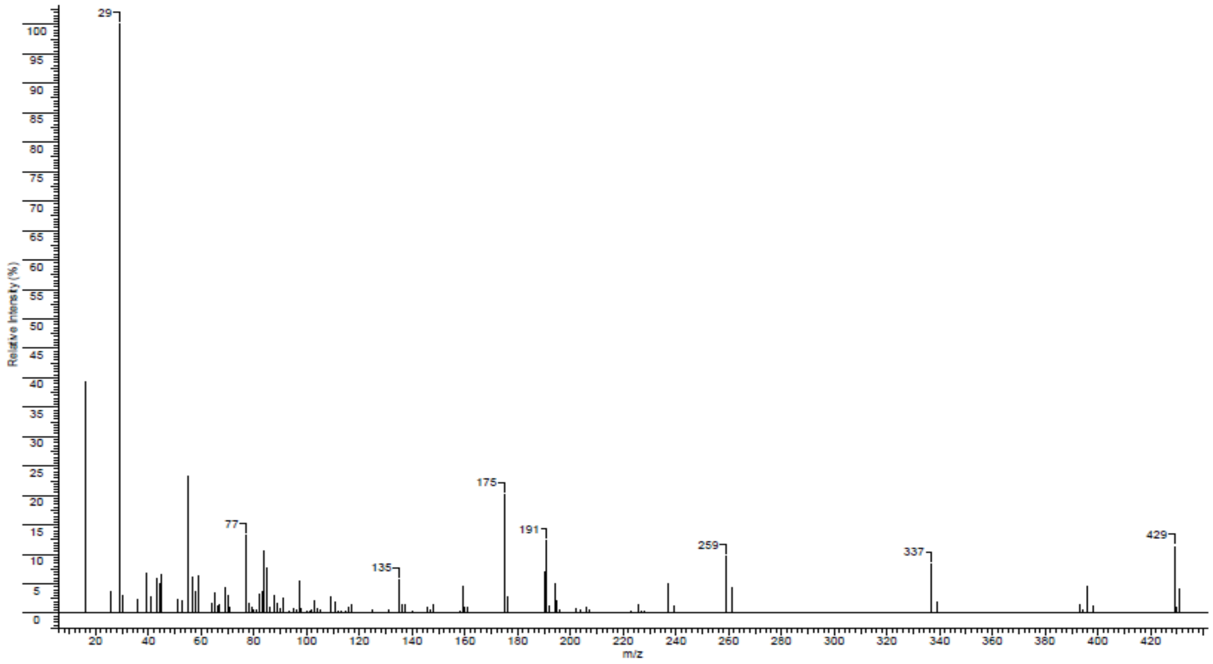

Figure S32. HRMS spectrum of dimethyl2-(1-chloro-2-(phenylamino)-2-thioxoethylidene)-1,3-dithiole-4,5-dicarboxylate (**12**).

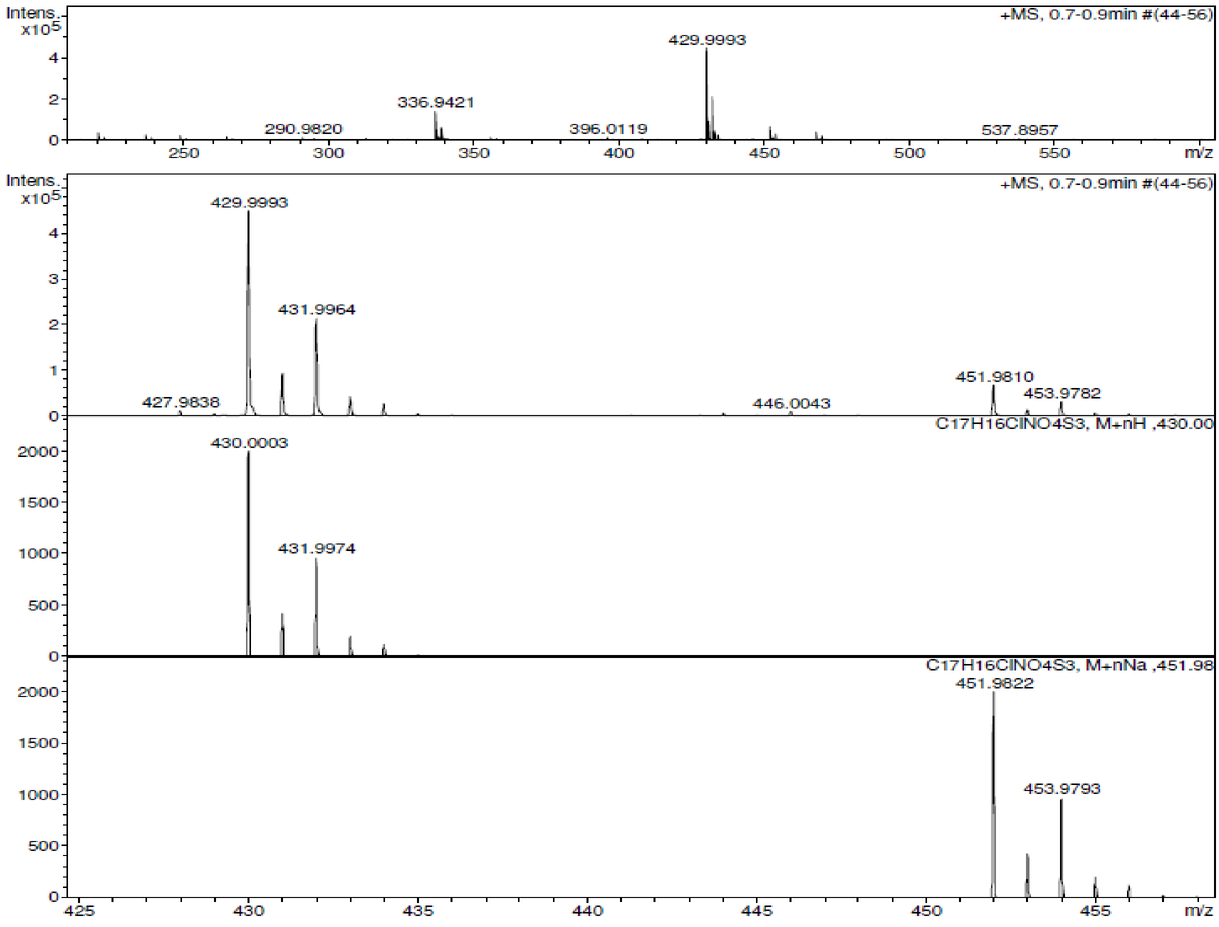

Figure S33. ^{1}H NMR spectrum of dimethyl 2-(4,5-bis(methoxycarbonyl)-2-(phenylimino)thiophen-3(2H)-ylidene)-1,3-dithiole-4,5-dicarboxylate (**10a**) in DMSO-d$_6$.

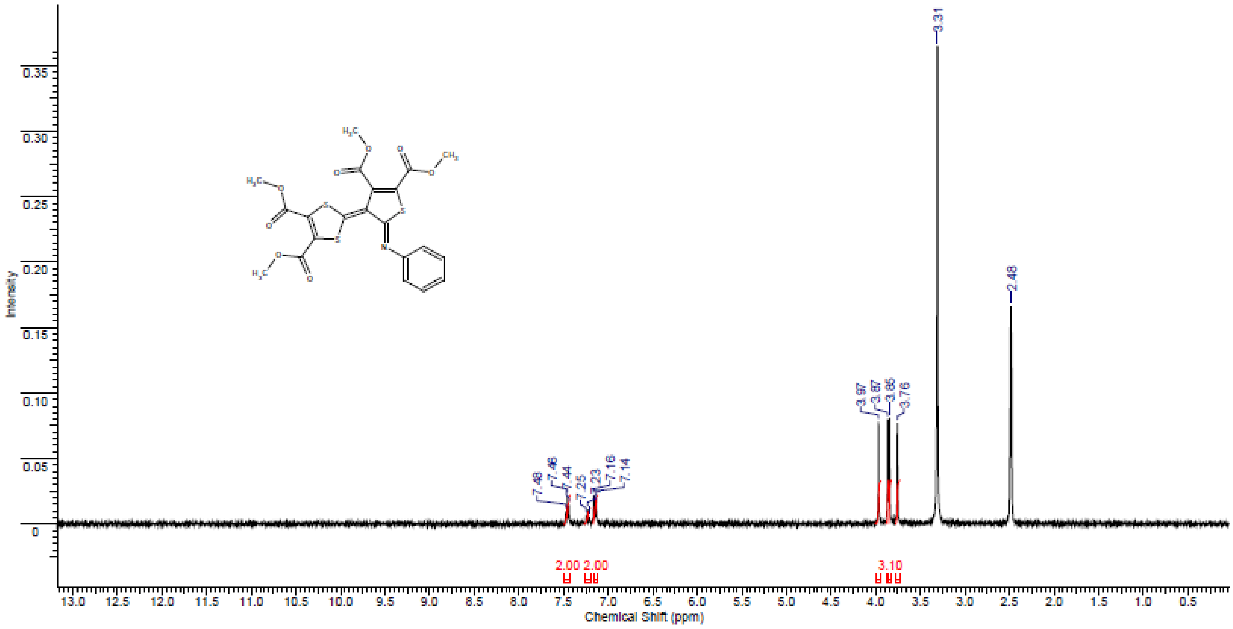

Figure S34. ^{13}C NMR spectrum of dimethyl 2-(4,5-bis(methoxycarbonyl)-2-(phenylimino)thiophen-3(2H)-ylidene)-1,3-dithiole-4,5-dicarboxylate (**10a**) in DMSO-d$_6$.

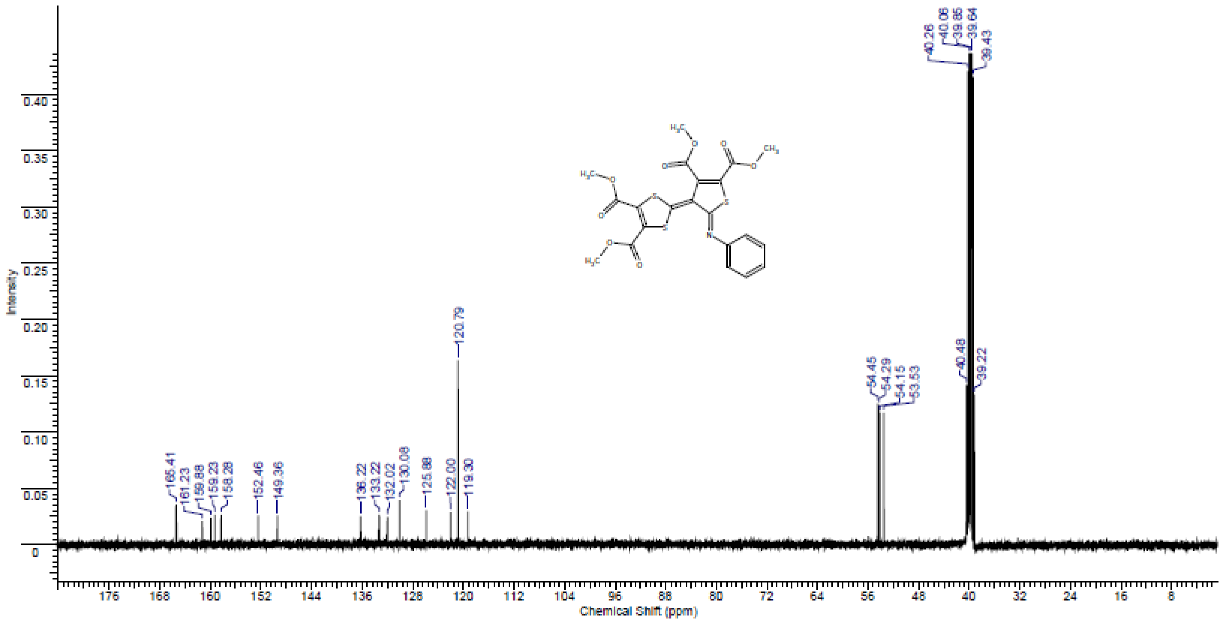

Figure S35. LRMS spectrum of dimethyl 2-(4,5-bis(methoxycarbonyl)-2-(phenylimino)thiophen-3(2H)-ylidene)-1,3-dithiole-4,5-dicarboxylate (**10a**).

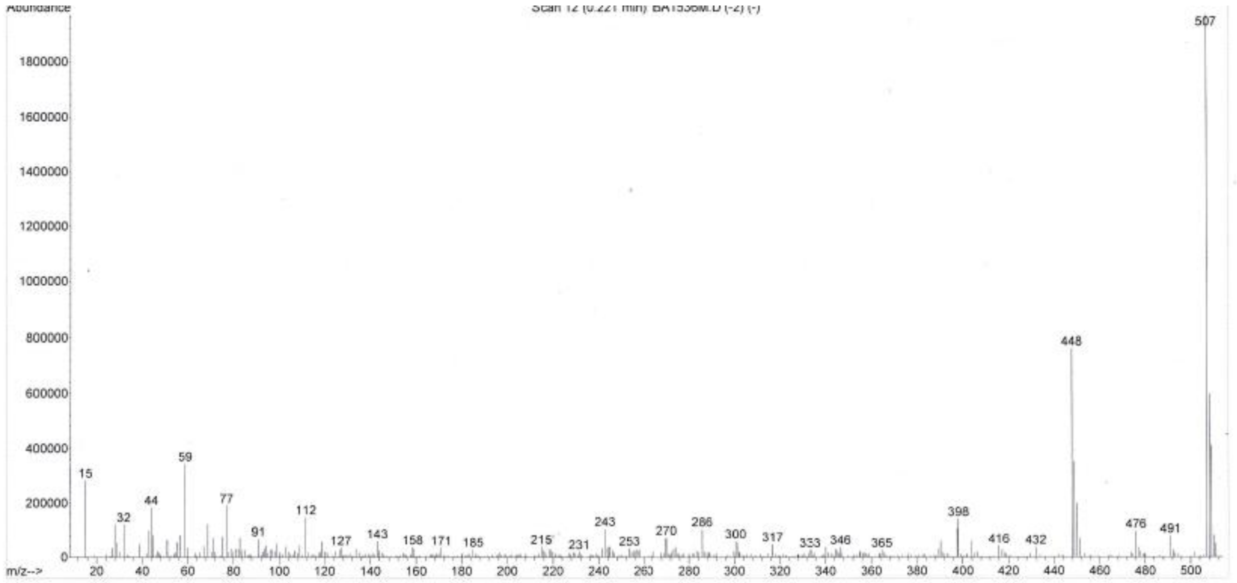

Figure S36. HRMS spectrum of dimethyl 2-(4,5-bis(methoxycarbonyl)-2-(phenylimino)thiophen-3(2H)-ylidene)-1,3-dithiole-4,5-dicarboxylate (**10a**).

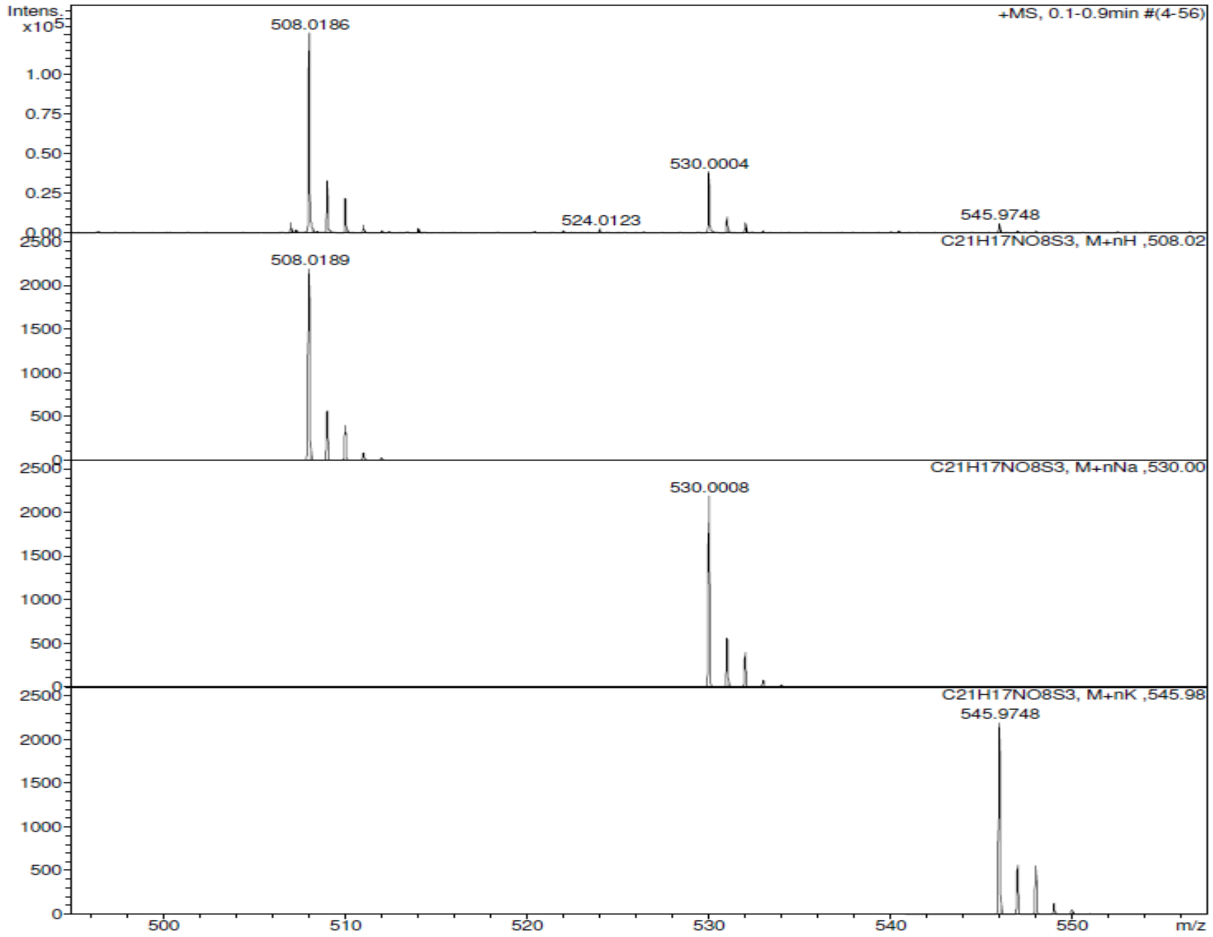

Figure S37. ^{1}H NMR spectrum of dimethyl 2-(4,5-bis(methoxycarbonyl)-2-(p-tolylimino)thiophen-3(2H)-ylidene)-1,3-dithiole-4,5-dicarboxylate (**10b**) in $CDCl_3$.

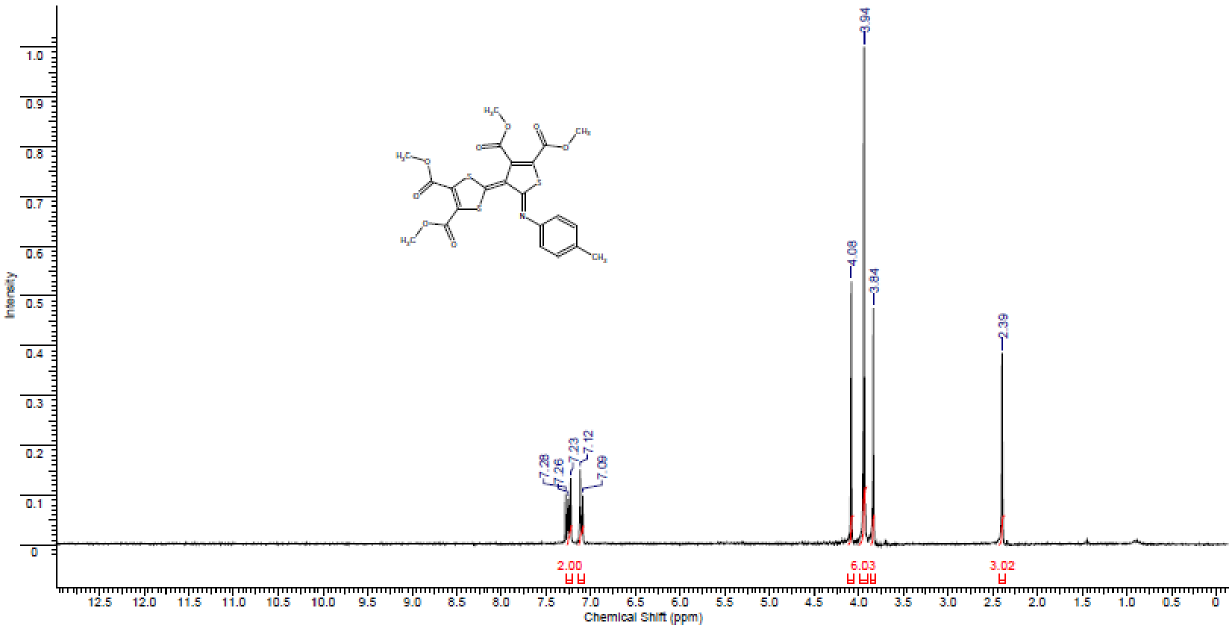

Figure S38. ^{13}C NMR spectrum of dimethyl 2-(4,5-bis(methoxycarbonyl)-2-(p-tolylimino)thiophen-3(2H)-ylidene)-1,3-dithiole-4,5-dicarboxylate (**10b**) in $CDCl_3$.

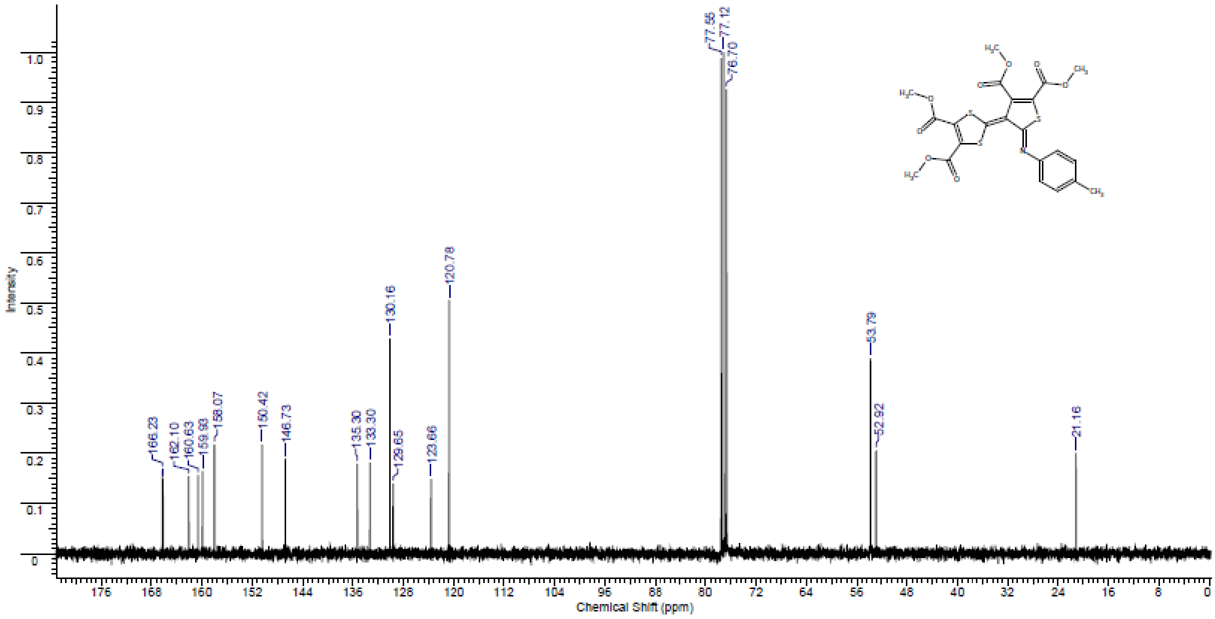

Figure S39. LRMS spectrum of dimethyl2-(4,5-bis(methoxycarbonyl)-2-(p-tolylimino)thiophen-3(2H)-ylidene)-1,3-dithiole-4,5-dicarboxylate (**10b**).

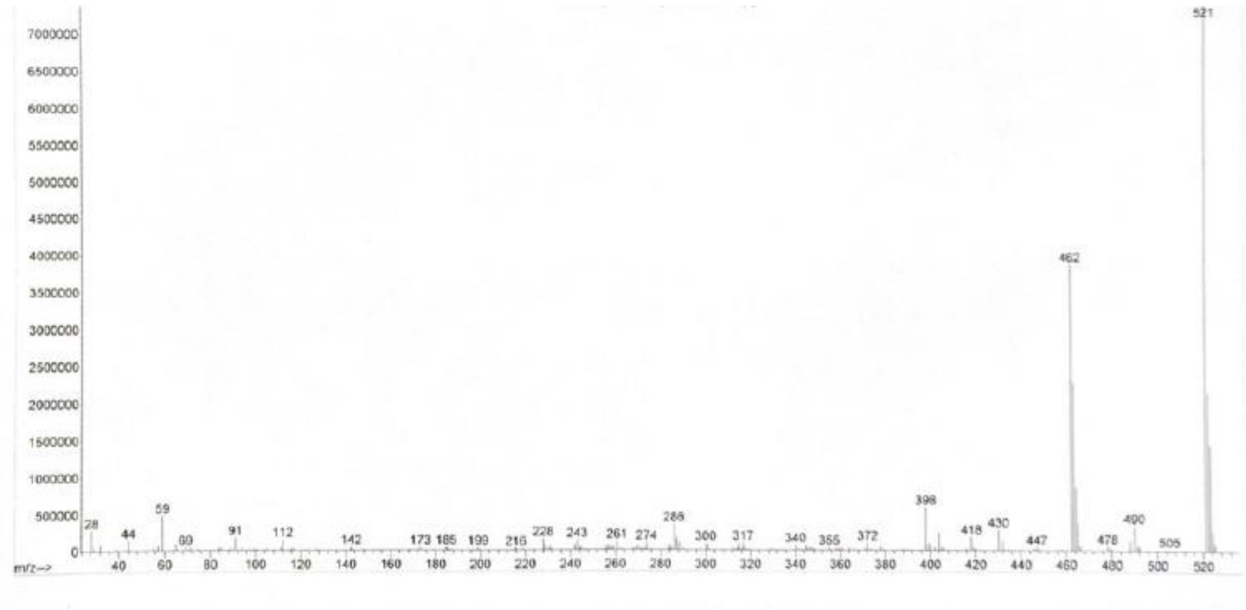

Figure S40. HRMS spectrum of dimethyl2-(4,5-bis(methoxycarbonyl)-2-(p-tolylimino)thiophen-3(2H)-ylidene)-1,3-dithiole-4,5-dicarboxylate (**10b**).

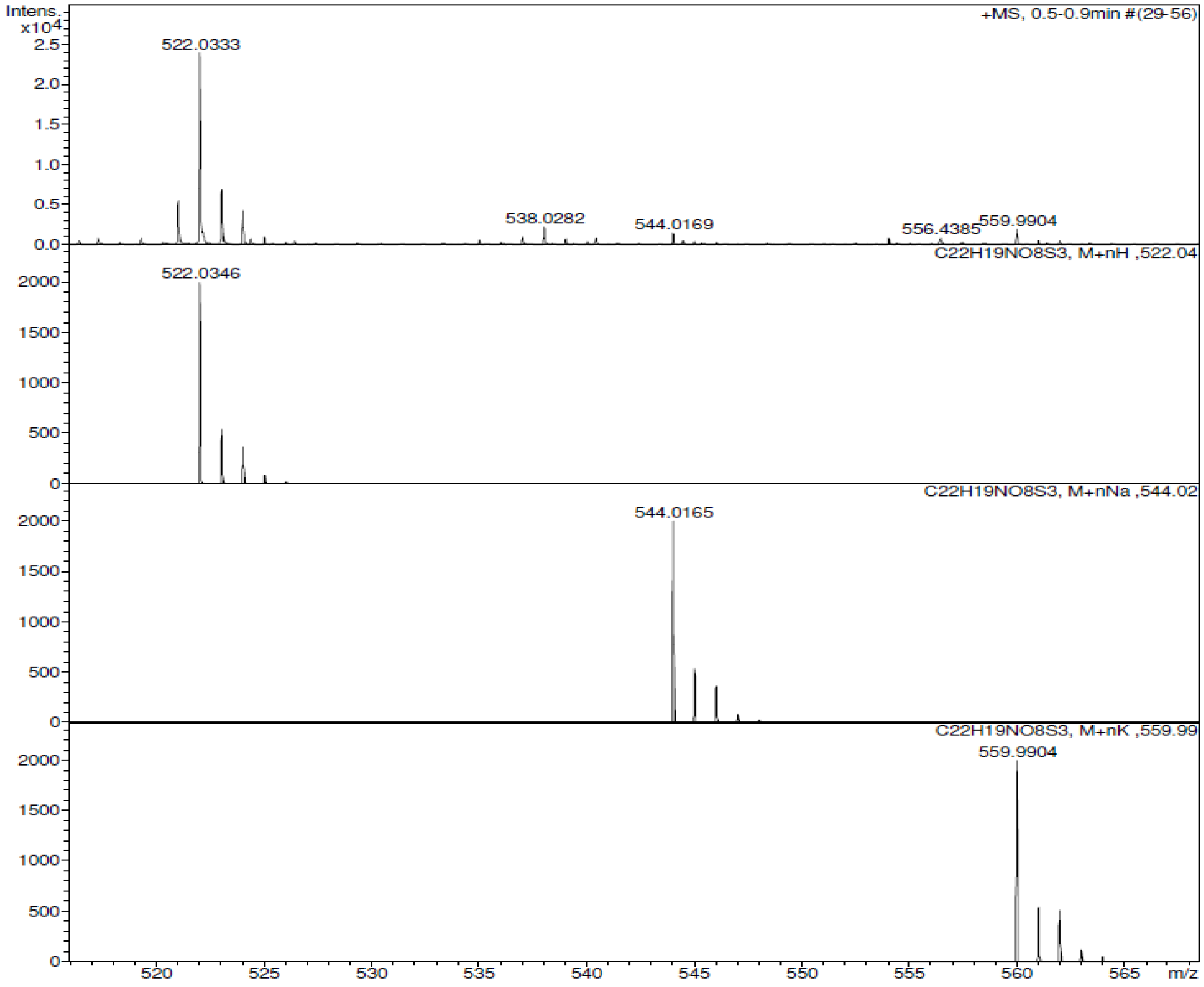

Figure S41. ^{1}H NMR spectrum of dimethyl 2-(4,5-bis(methoxycarbonyl)-2-((4-methoxyphenyl)imino)thiophen-3(2H)-ylidene)-1,3-dithiole-4,5-dicarboxylate (**10c**) in $CDCl_3$.

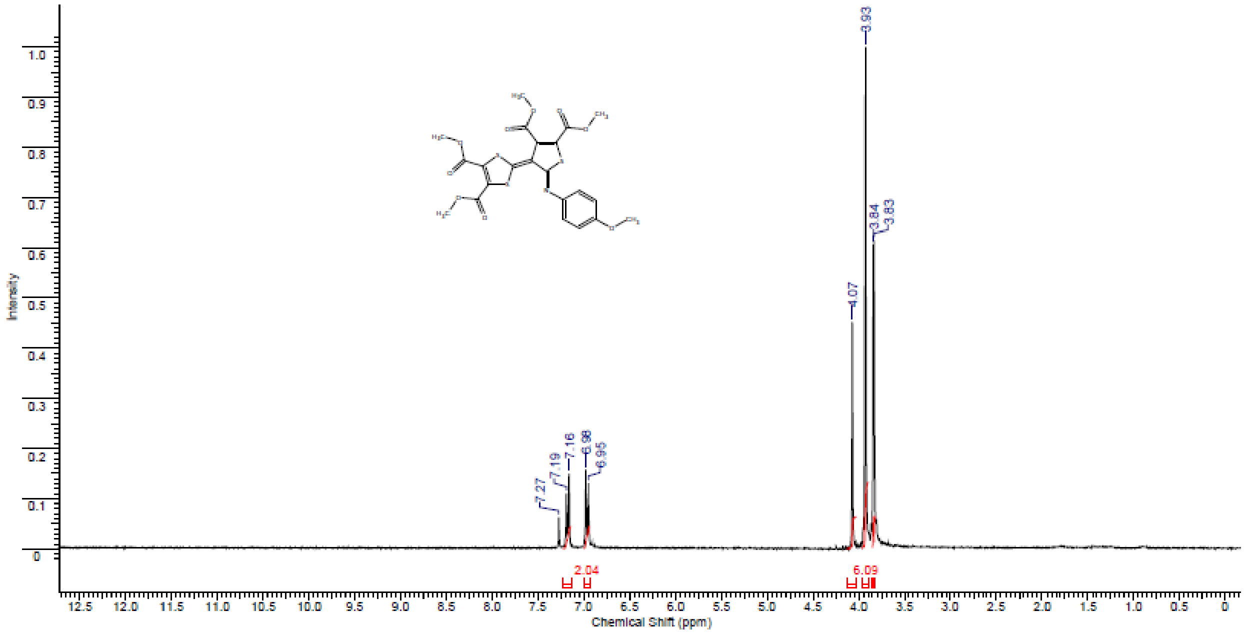

Figure S42. ^{13}C NMR spectrum of dimethyl 2-(4,5-bis(methoxycarbonyl)-2-((4-methoxyphenyl)imino)thiophen-3(2H)-ylidene)-1,3-dithiole-4,5-dicarboxylate (**10c**) in $CDCl_3$.

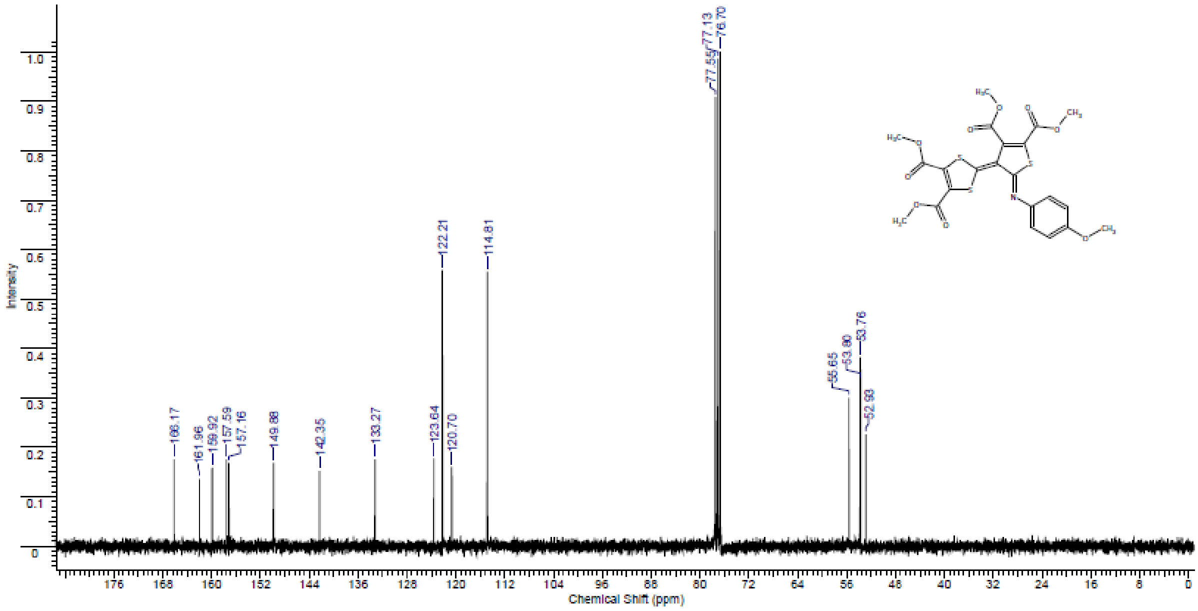

Figure S43. LRMS spectrum of dimethyl2-(4,5-bis(methoxycarbonyl)-2-((4-methoxyphenyl)imino)thiophen-3(2H)-ylidene)-1,3-dithiole-4,5-dicarboxylate (**10c**).

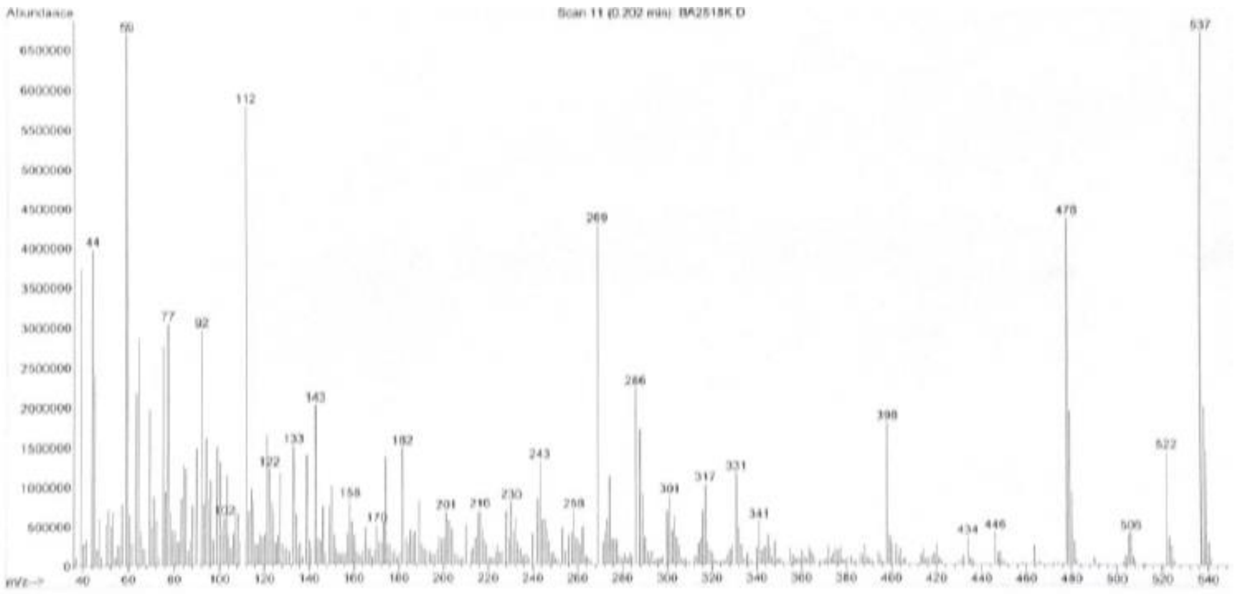

Figure S44. HRMS spectrum of dimethyl2-(4,5-bis(methoxycarbonyl)-2-((4-methoxyphenyl)imino)thiophen-3(2H)-ylidene)-1,3-dithiole-4,5-dicarboxylate (**10c**).

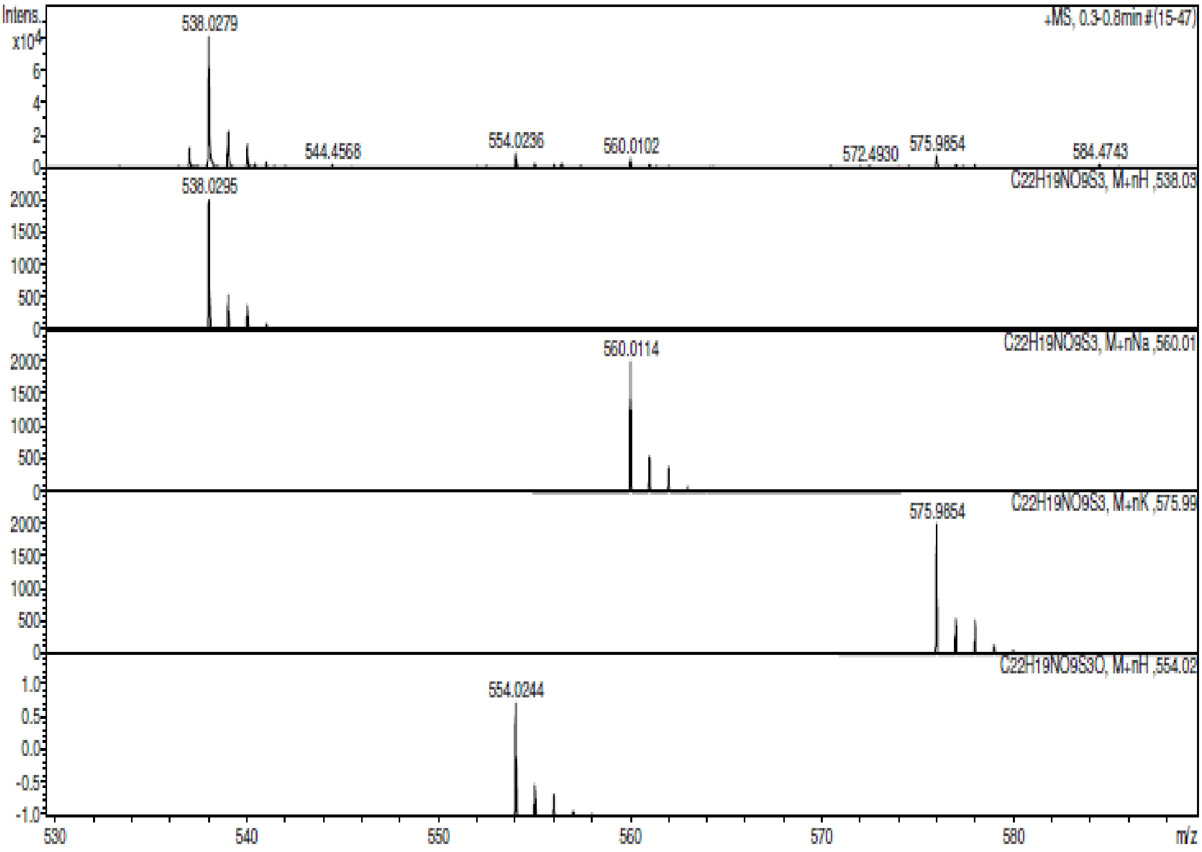

Figure S45. ^{1}H NMR spectrum of dimethyl 2-(4,5-bis(methoxycarbonyl)-2-((4-nitrophenyl)imino)thiophen-3(2H)-ylidene)-1,3-dithiole-4,5-dicarboxylate (**10d**) in $CDCl_3$.

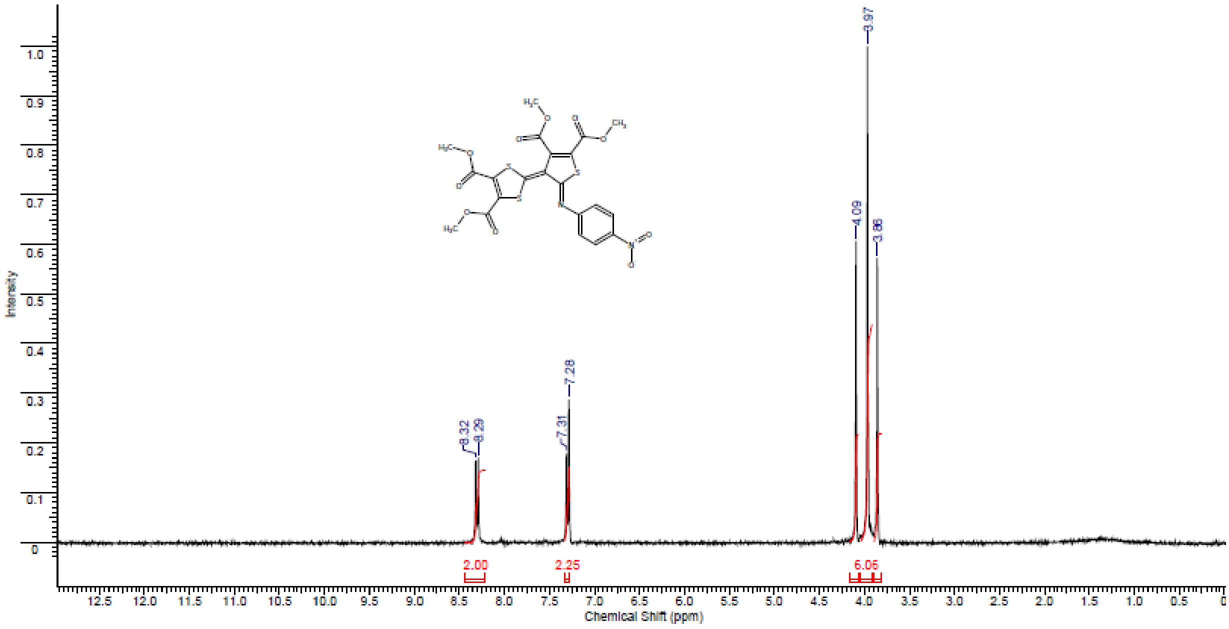

Figure S46. ^{13}C NMR spectrum of dimethyl 2-(4,5-bis(methoxycarbonyl)-2-((4-nitrophenyl)imino)thiophen-3(2H)-ylidene)-1,3-dithiole-4,5-dicarboxylate (**10d**) in $CDCl_3$.

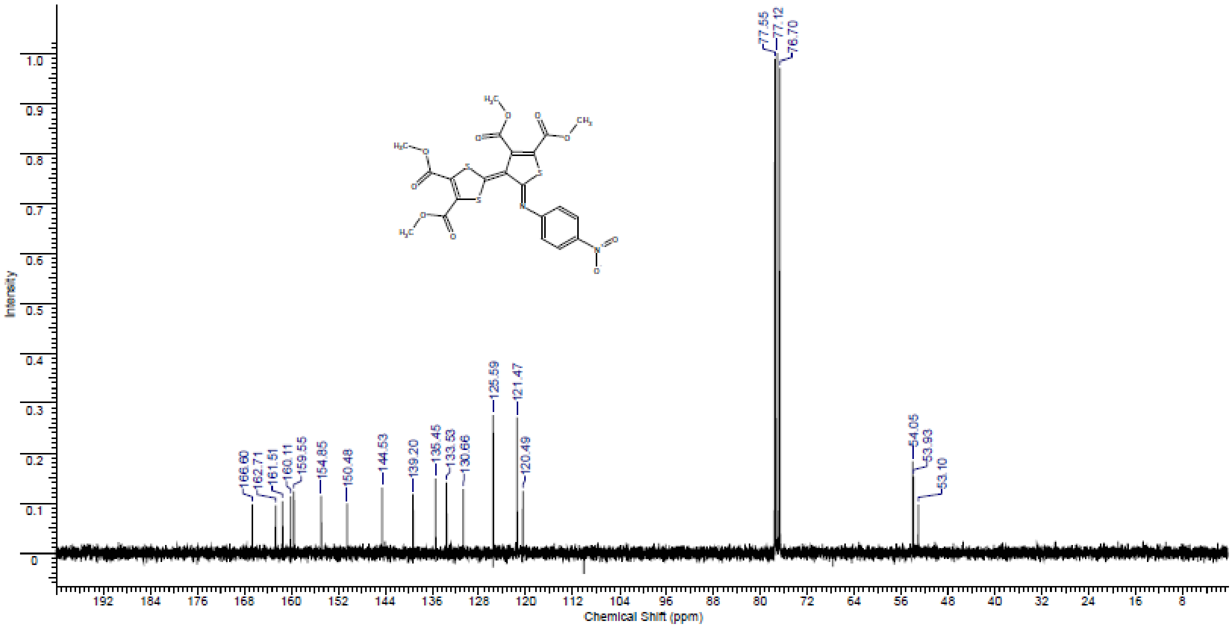

Figure S47. LRMS spectrum of dimethyl2-(4,5-bis(methoxycarbonyl)-2-((4-nitrophenyl)imino)thiophen-3(2H)-ylidene)-1,3-dithiole-4,5-dicarboxylate (**10d**).

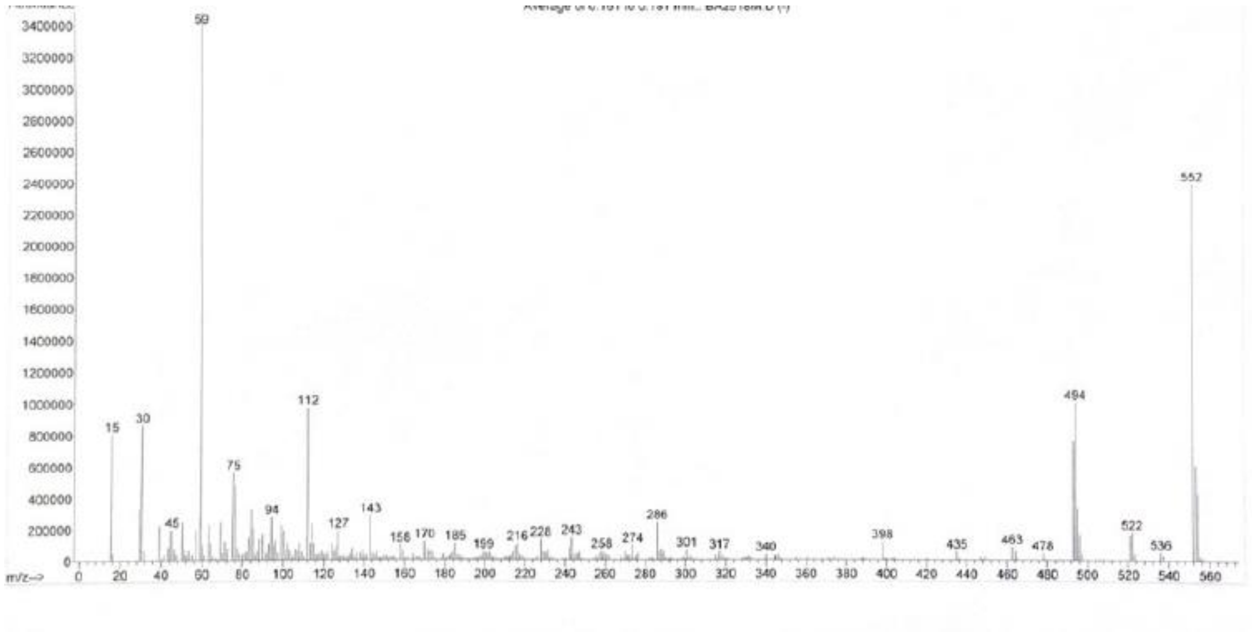

Figure S48. HRMS spectrum of dimethyl2-(4,5-bis(methoxycarbonyl)-2-((4-nitrophenyl)imino)thiophen-3(2H)-ylidene)-1,3-dithiole-4,5-dicarboxylate (**10d**).

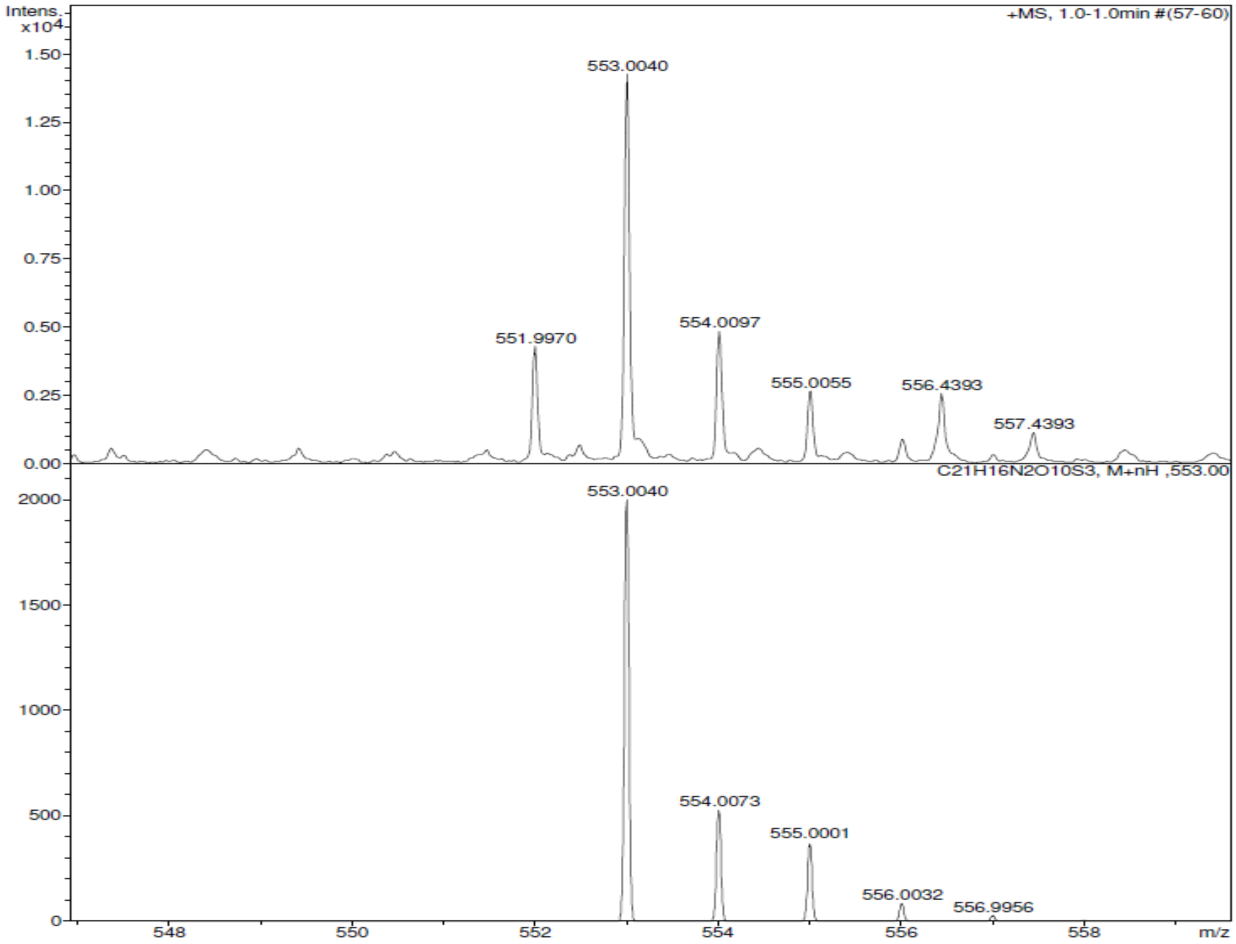

Figure S49. ^{1}H NMR spectrum of dimethyl 2-(4,5-bis(methoxycarbonyl)-2-(methylimino)thiophen-3(2H)-ylidene)-1,3-dithiole-4,5-dicarboxylate (**10e**) in $CDCl_3$.

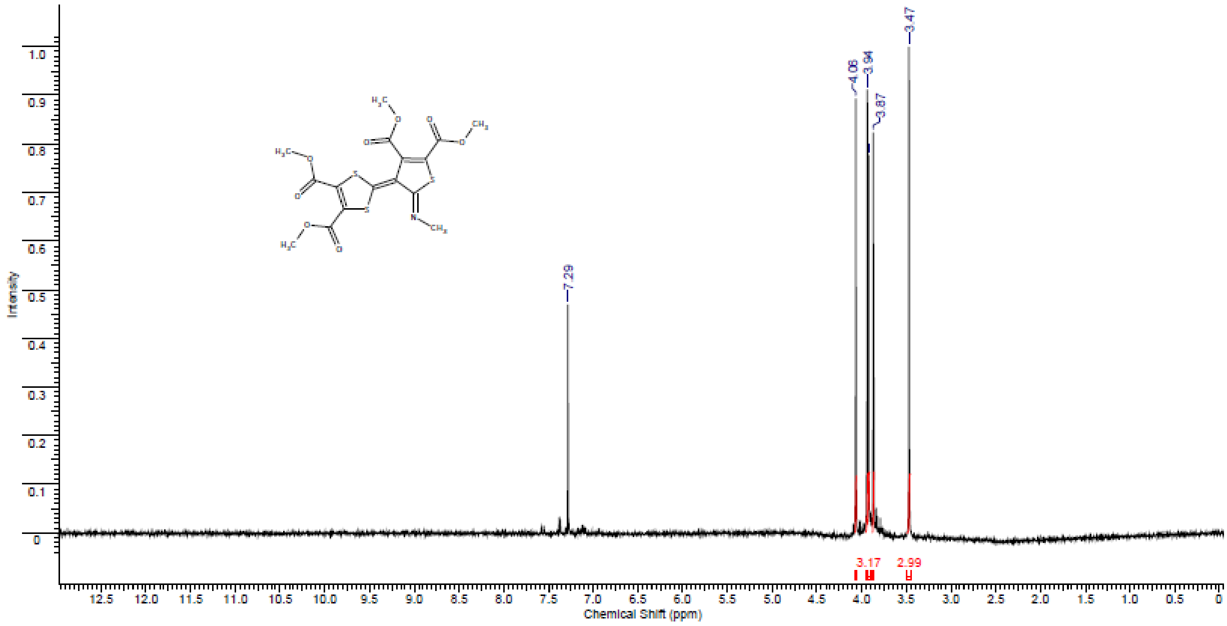

Figure S50. ^{13}C NMR spectrum of dimethyl 2-(4,5-bis(methoxycarbonyl)-2-(methylimino)thiophen-3(2H)-ylidene)-1,3-dithiole-4,5-dicarboxylate (**10e**) in $CDCl_3$.

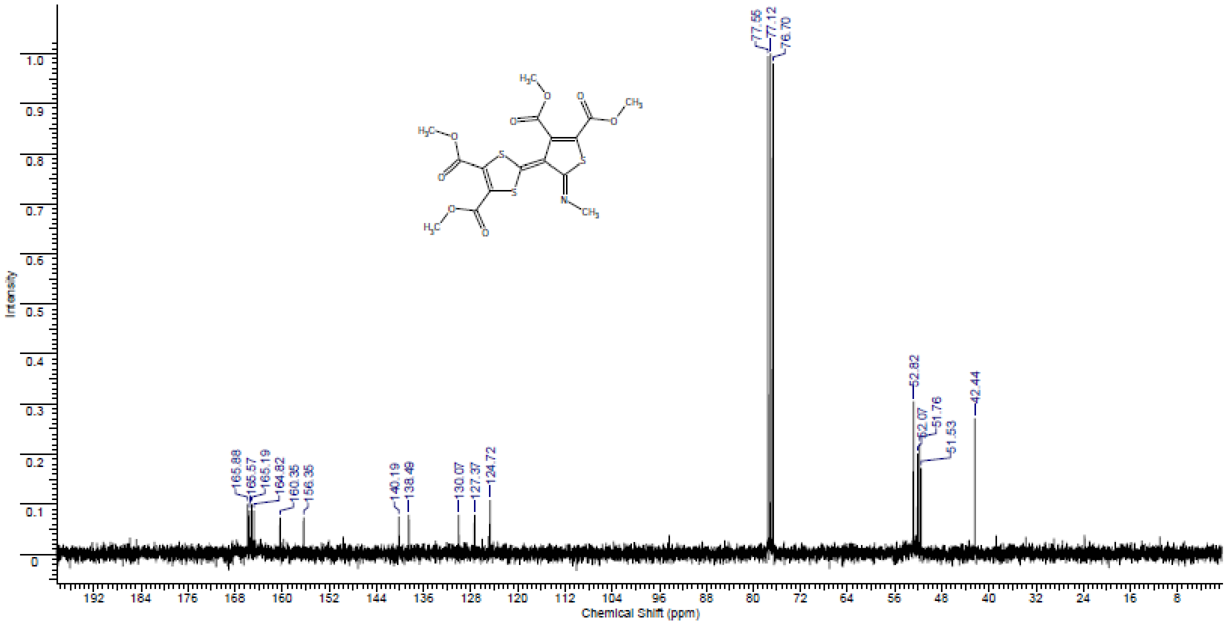

Figure S51. LRMS spectrum of dimethyl2-(4,5-bis(methoxycarbonyl)-2-(methylimino)thiophen-3(2H)-ylidene)-1,3-dithiole-4,5-dicarboxylate (**10e**).

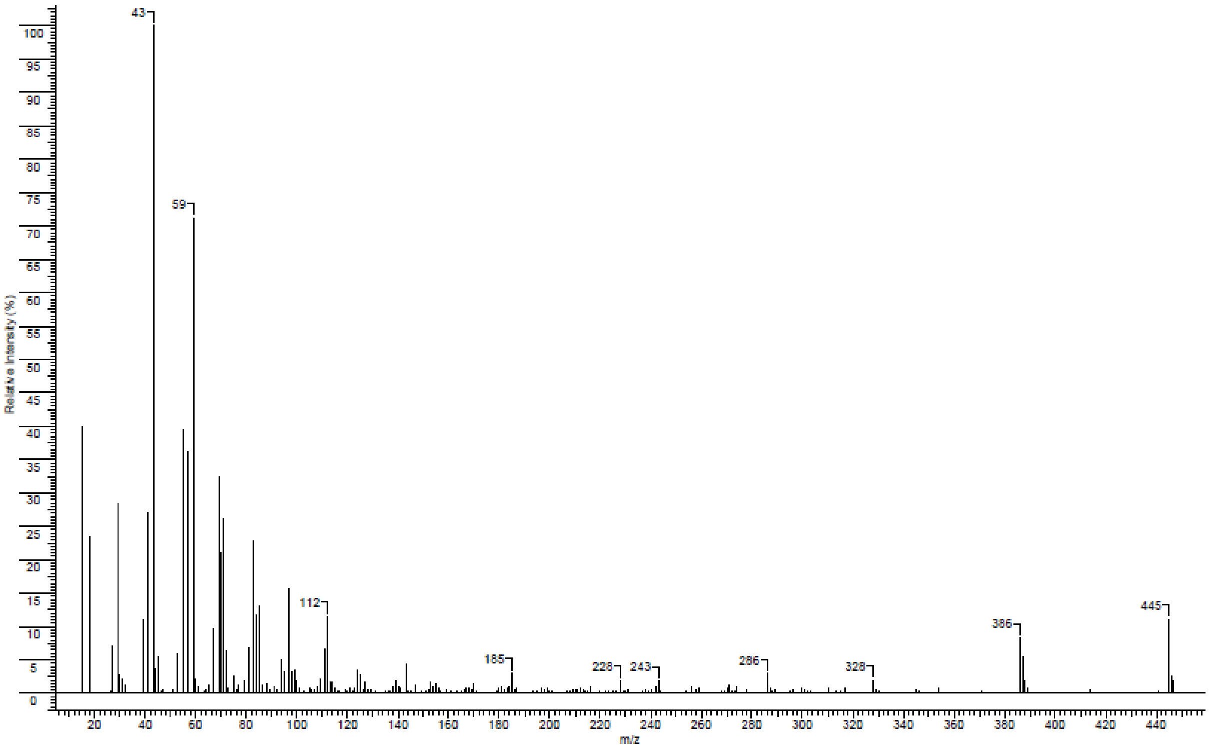

Figure S52. HRMS spectrum of dimethyl2-(4,5-bis(methoxycarbonyl)-2-(methylimino)thiophen-3(2H)-ylidene)-1,3-dithiole-4,5-dicarboxylate (**10e**).

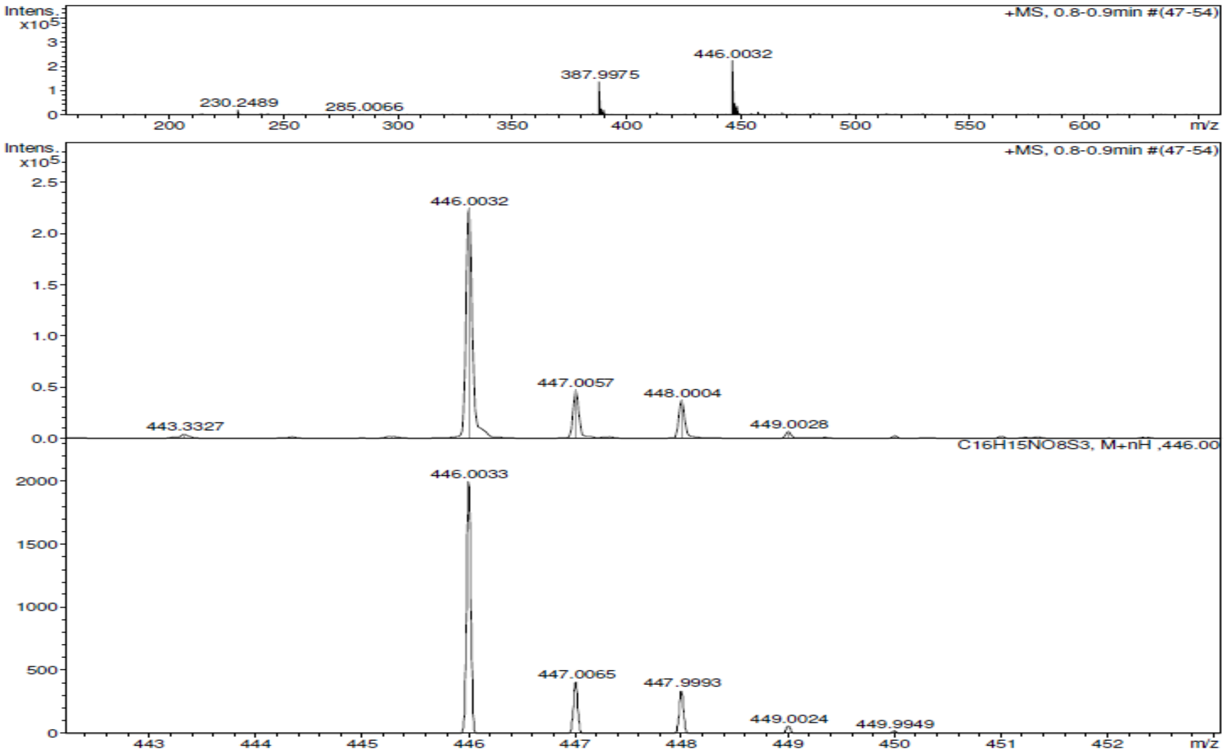

Figure S53. ^{1}H NMR spectrum of dimethyl 2-(2-(tert-butylimino)-4,5-bis(methoxycarbonyl)thiophen-3(2H)-ylidene)-1,3-dithiole-4,5-dicarboxylate (**10f**) in $CDCl_3$.

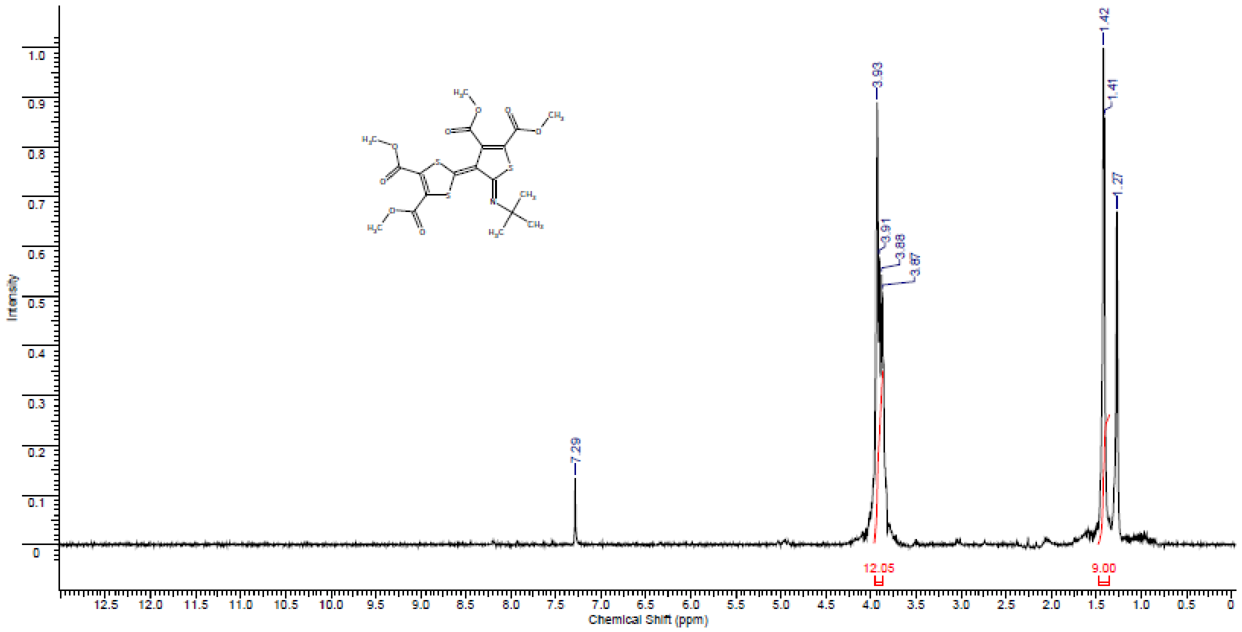

Figure S54. ^{13}C NMR spectrum of dimethyl 2-(2-(tert-butylimino)-4,5-bis(methoxycarbonyl)thiophen-3(2H)-ylidene)-1,3-dithiole-4,5-dicarboxylate (**10f**) in $CDCl_3$.

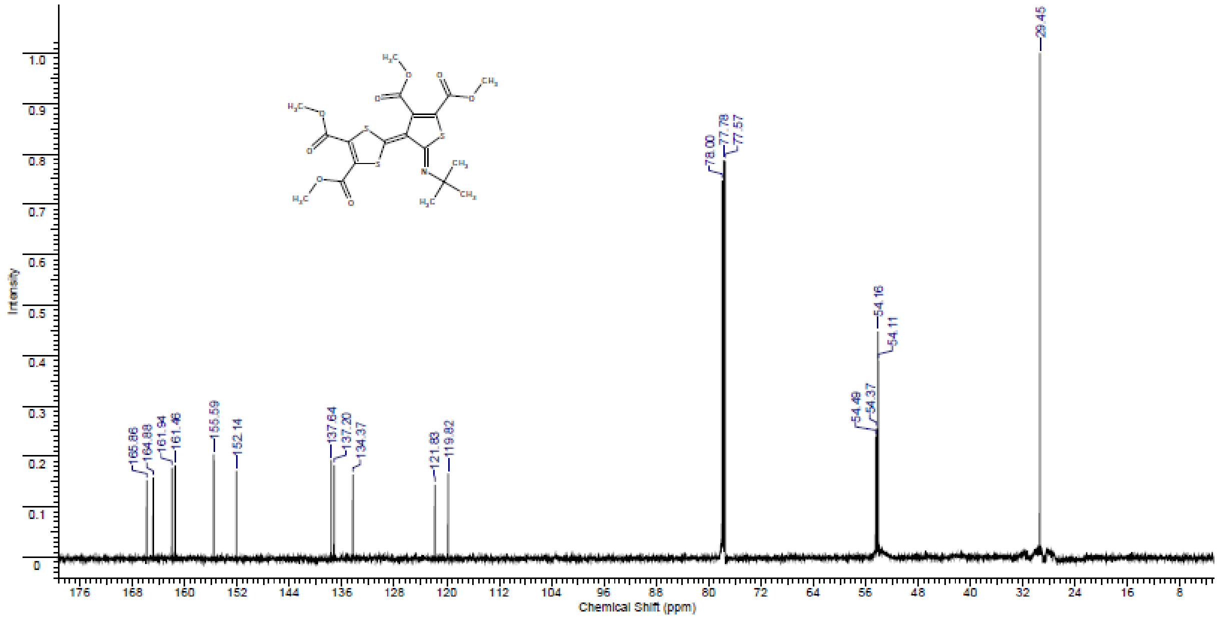

Figure S55. LRMS spectrum of dimethyl2-(2-(tert-butylimino)-4,5-bis(methoxycarbonyl)thiophen-3(2H)-ylidene)-1,3-dithiole-4,5-dicarboxylate (**10f**).

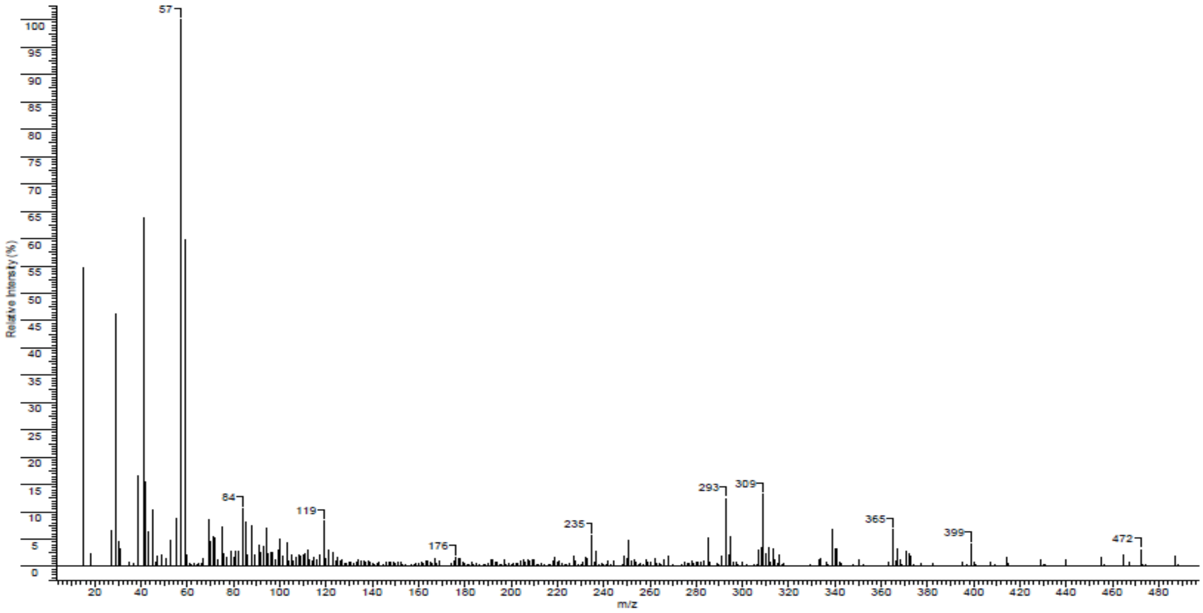

Figure S56. HRMS spectrum of dimethyl2-(2-(tert-butylimino)-4,5-bis(methoxycarbonyl)thiophen-3(2H)-ylidene)-1,3-dithiole-4,5-dicarboxylate (**10f**).

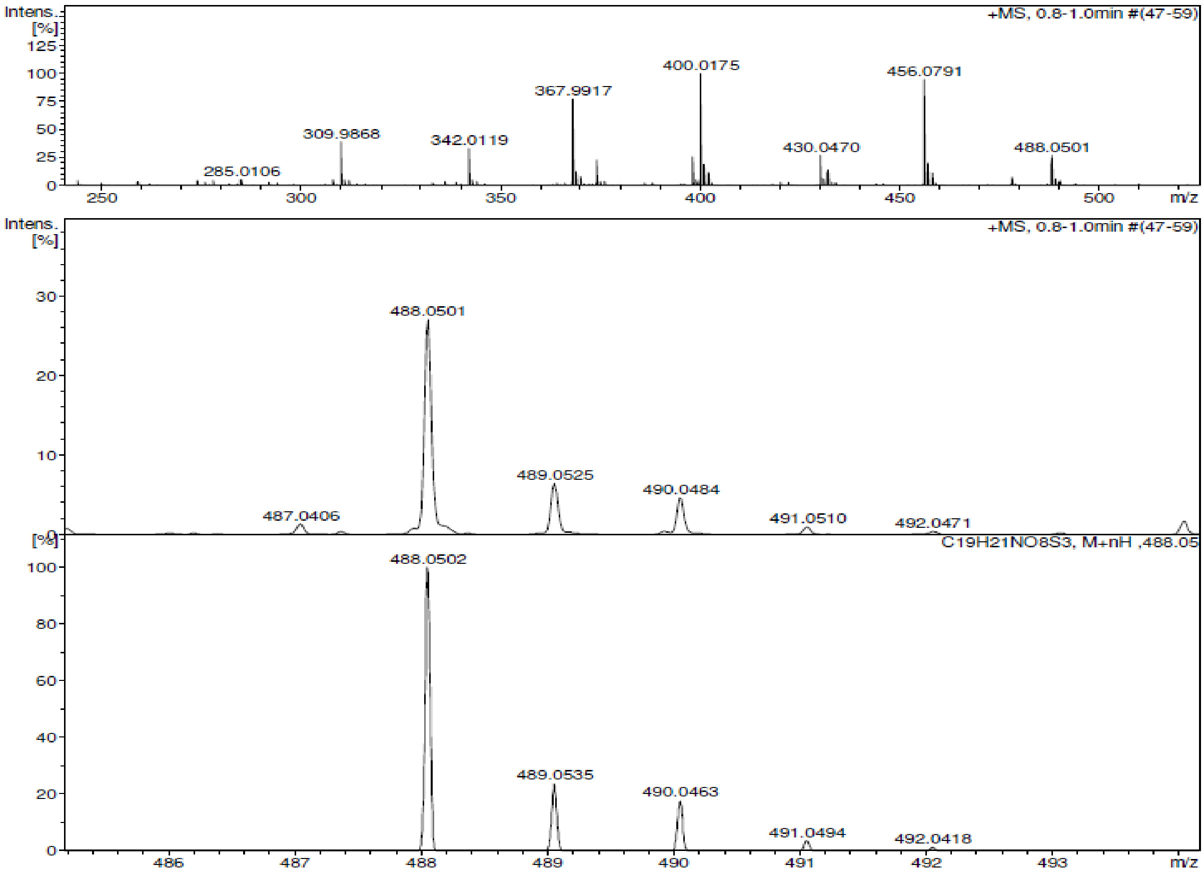

Figure S57. ^{1}H NMR spectrum of dimethyl 2-(4,5-bis(ethoxycarbonyl)-2-(phenylimino)thiophen-3(2H)-ylidene)-1,3-dithiole-4,5-dicarboxylate (**11a**) in $CDCl_3$.

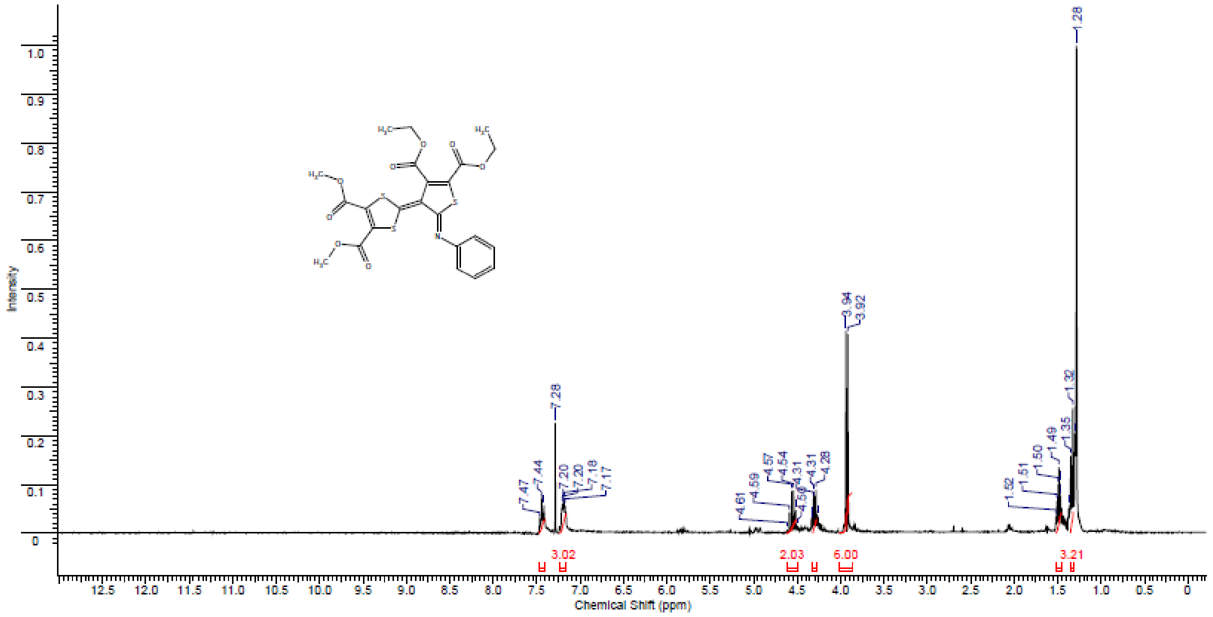

Figure S58. ^{13}C NMR spectrum of dimethyl 2-(4,5-bis(ethoxycarbonyl)-2-(phenylimino)thiophen-3(2H)-ylidene)-1,3-dithiole-4,5-dicarboxylate (**11a**) in $CDCl_3$.

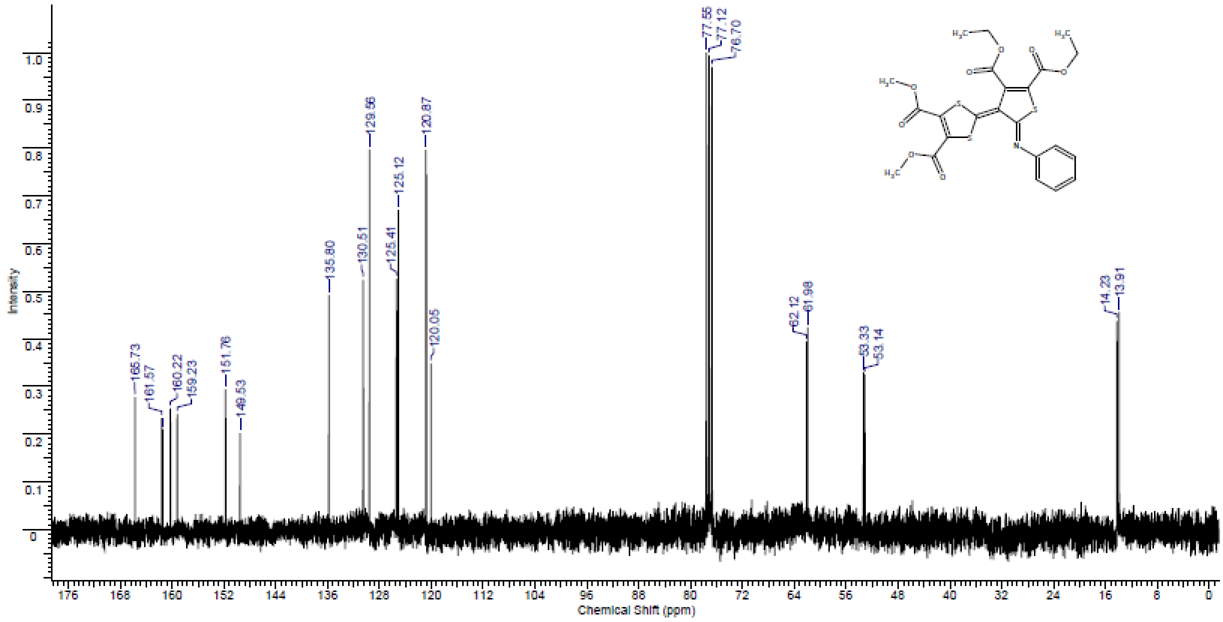

Figure S59. LRMS spectrum of dimethyl 2-(4,5-bis(ethoxycarbonyl)-2-(phenylimino)thiophen-3(2H)-ylidene)-1,3-dithiole-4,5-dicarboxylate (**11a**).

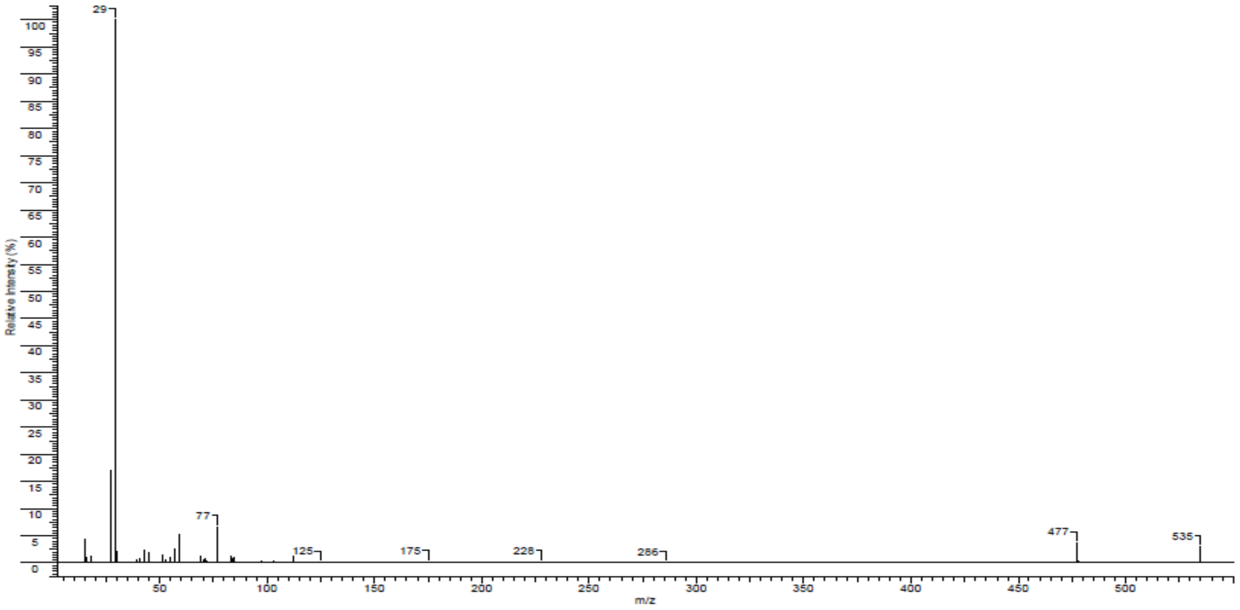

Figure S60. HRMS spectrum of dimethyl 2-(4,5-bis(ethoxycarbonyl)-2-(phenylimino)thiophen-3(2H)-ylidene)-1,3-dithiole-4,5-dicarboxylate (**11a**).

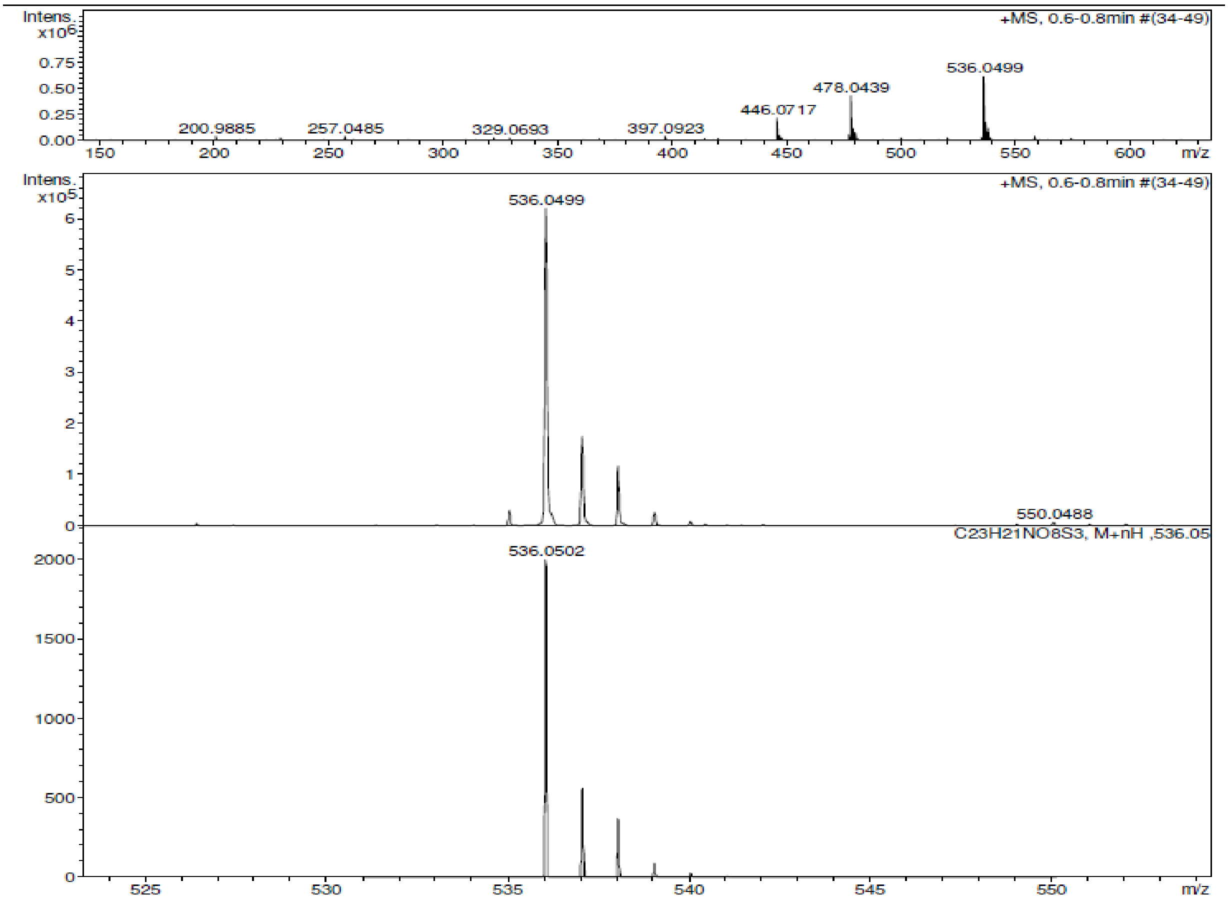

Figure S61. ^{1}H NMR spectrum of diethyl 2-(4,5-bis(methoxycarbonyl)-2-(phenylimino)thiophen-3(2H)-ylidene)-1,3-dithiole-4,5-dicarboxylate (**11b**) in $CDCl_3$.

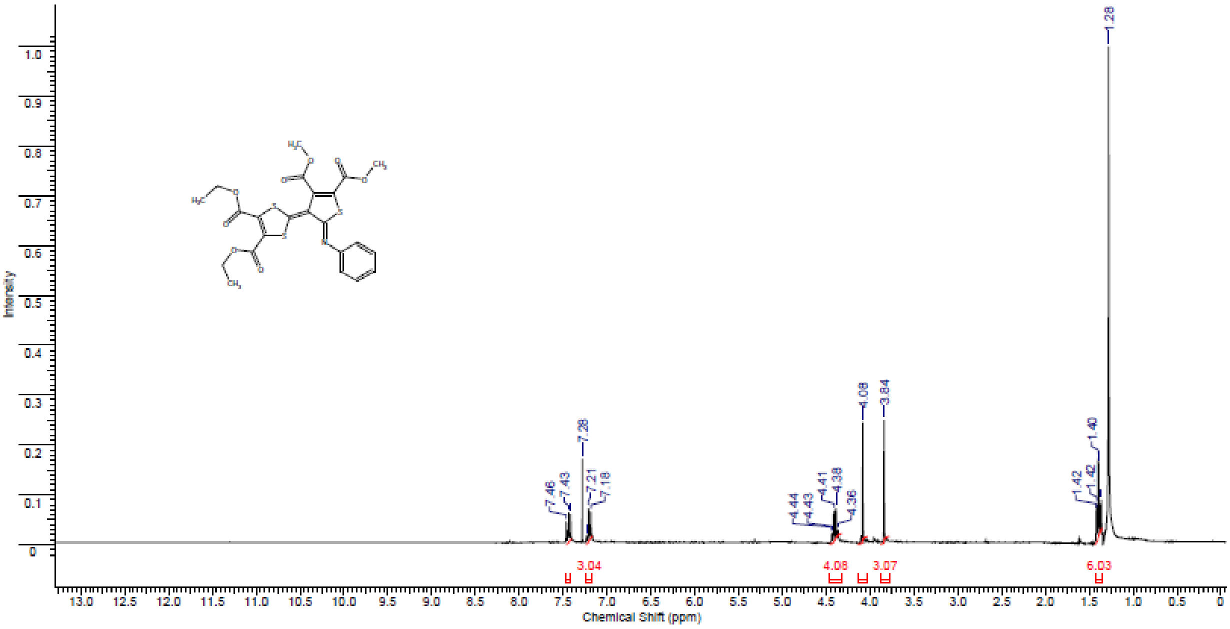

Figure S62. ^{13}C NMR spectrum of diethyl 2-(4,5-bis(methoxycarbonyl)-2-(phenylimino)thiophen-3(2H)-ylidene)-1,3-dithiole-4,5-dicarboxylate (**11b**) in $CDCl_3$.

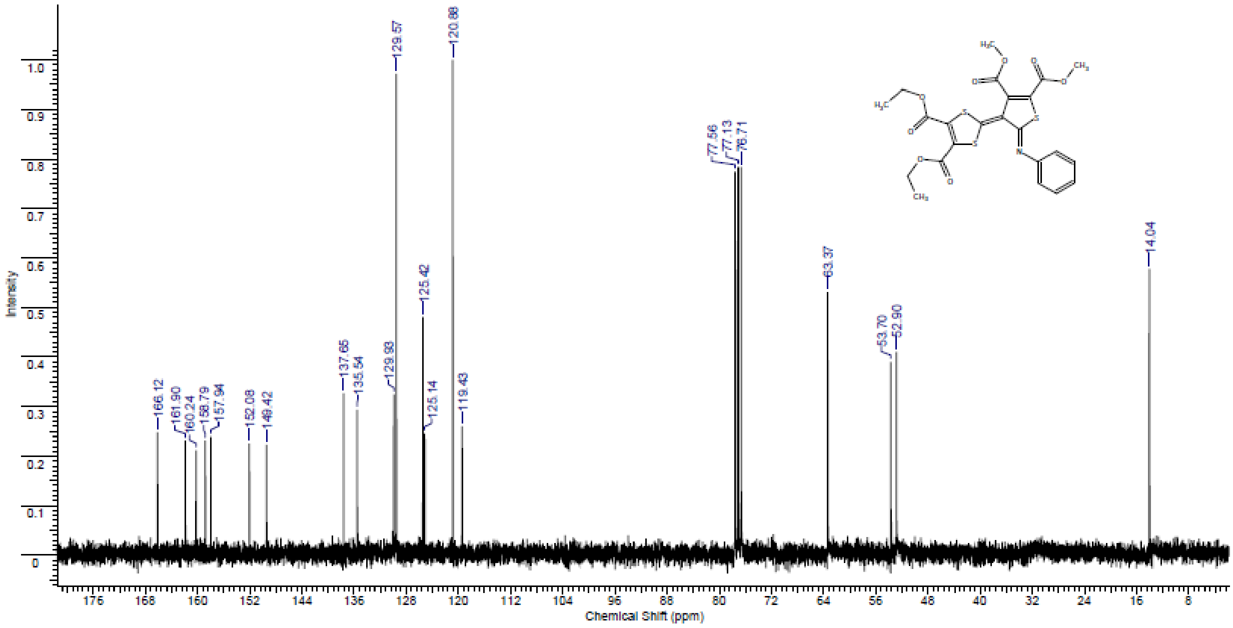

Figure S63. LRMS spectrum of dimethyl 2-(4,5-bis(methoxycarbonyl)-2-(phenylimino)thiophen-3(2H)-ylidene)-1,3-dithiole-4,5-dicarboxylate (**11b**).

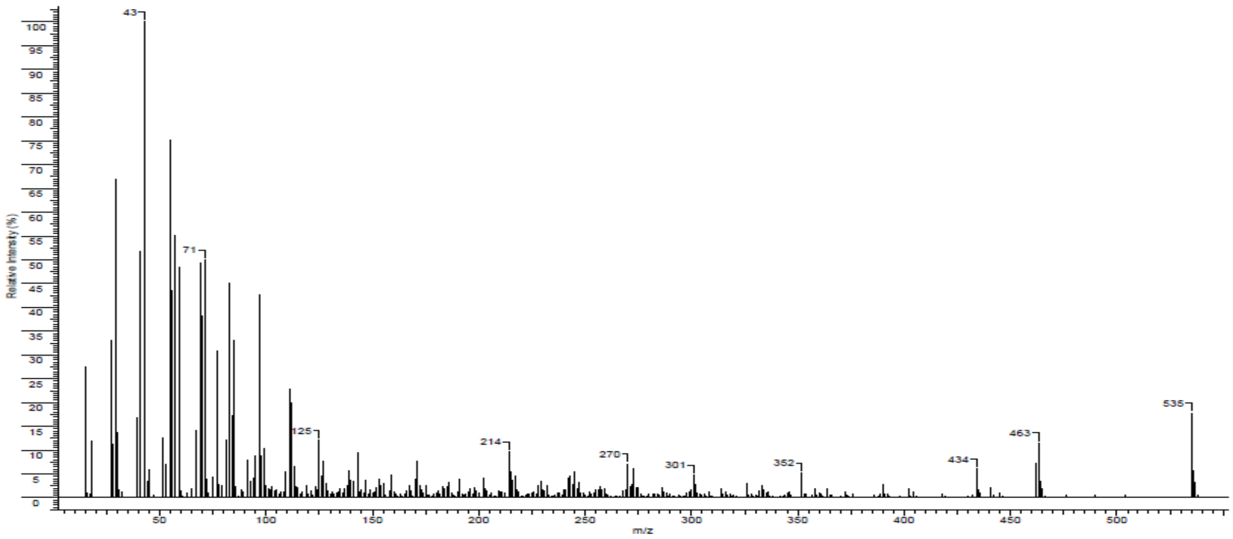

Figure S64. HRMS spectrum of dimethyl 2-(4,5-bis(methoxycarbonyl)-2-(phenylimino)thiophen-3(2H)-ylidene)-1,3-dithiole-4,5-dicarboxylate (**11b**).

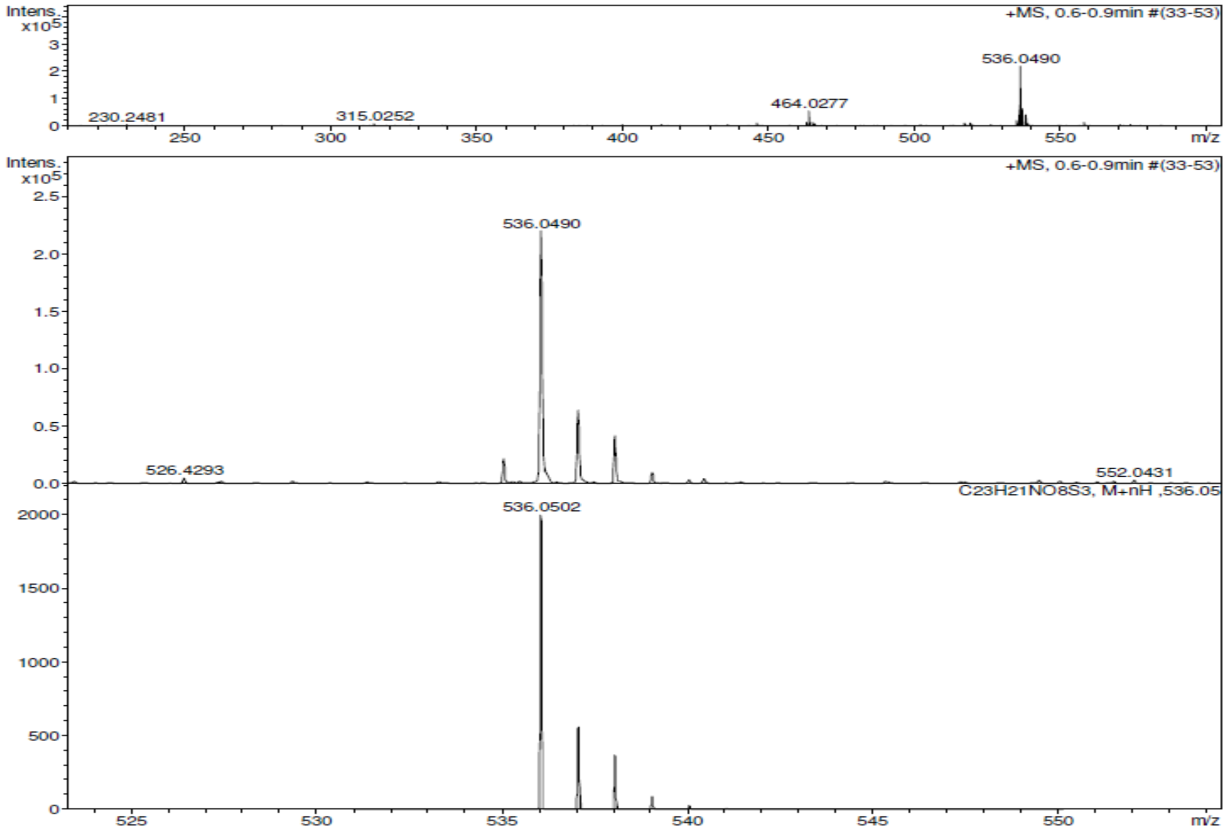

Table S1. Crystal data and structure refinement for compound 10a (No. CCDC 2205782).

Identification code	VAO236	
Empirical formula	C21 H17 N O8 S3	
Formula weight	507.54	
Temperature	100(2) K	
Wavelength	0.71073 Å	
Crystal system	Triclinic	
Space group	P-1	
Unit cell dimensions	a = 7.4551(7) Å	□= 87.787(3)°.
	b = 14.1465(13) Å	□= 81.400(3)°.
	c = 20.9888(18) Å	□ = 76.890(3)°.
Volume	2131.6(3) $Å^3$	
Z	4	
Density (calculated)	1.582 g/cm^3	
Absorption coefficient	0.399 mm^{-1}	
F(000)	1048	
Crystal size	0.17 x 0.11 x 0.09 mm^3	
Theta range for data collection	2.451 to 32.034°.	
Index ranges	-11<=h<=11, -21<=k<=21, -31<=l<=31	
Reflections collected	91687	
Independent reflections	14809 [R(int) = 0.0984]	
Observed reflections	9749	
Completeness to theta = 25.242°	99.9 %	
Absorption correction	Semi-empirical from equivalents	
Max. and min. transmission	0.7464 and 0.6165	
Refinement method	Full-matrix least-squares on F^2	
Data / restraints / parameters	14809 / 0 / 603	
Goodness-of-fit on F^2	1.031	
Final R indices [I>2sigma(I)]	R1 = 0.0571, wR2 = 0.1294	
R indices (all data)	R1 = 0.1011, wR2 = 0.1540	
Largest diff. peak and hole	0.670 and -0.754 e.$Å^{-3}$	

Table S2. Atomic coordinates (x 10^4) and equivalent isotropic displacement parameters ($Å^2$x 10^3) for compound 10a (No. CCDC 2205782). U(eq) is defined as one third of the trace of the orthogonalized U^{ij} tensor.

	x	y	z	U(eq)
S(1)	6345(1)	5212(1)	6249(1)	21(1)
S(2)	6641(1)	6049(1)	4954(1)	20(1)
S(3)	10025(1)	2809(1)	4327(1)	22(1)
O(1)	4819(3)	8540(1)	5750(1)	28(1)
O(2)	4588(3)	7926(1)	4799(1)	26(1)
O(3)	4474(3)	6487(1)	7317(1)	28(1)
O(4)	2842(3)	7618(1)	6713(1)	27(1)
O(5)	6637(3)	2790(1)	6418(1)	27(1)
O(6)	9075(3)	3365(1)	6613(1)	25(1)
O(7)	11204(3)	875(1)	4897(1)	32(1)
O(8)	10275(3)	1309(1)	5937(1)	26(1)
N(1)	8316(3)	4739(2)	4118(1)	20(1)
C(1)	7183(3)	5026(2)	5440(1)	19(1)
C(2)	5440(3)	6817(2)	5590(1)	21(1)
C(3)	5238(3)	6418(2)	6187(1)	20(1)
C(4)	8777(3)	4022(2)	4499(1)	20(1)
C(5)	8180(3)	4133(2)	5192(1)	19(1)
C(6)	8680(3)	3232(2)	5534(1)	20(1)
C(7)	9684(3)	2492(2)	5143(1)	21(1)
C(8)	4883(3)	7868(2)	5413(1)	20(1)
C(9)	4128(4)	8902(2)	4541(1)	31(1)
C(10)	4160(4)	6848(2)	6804(1)	21(1)
C(11)	1728(4)	8096(2)	7284(1)	31(1)
C(12)	8686(3)	4752(2)	3442(1)	20(1)
C(13)	10050(4)	4084(2)	3058(1)	24(1)
C(14)	10277(4)	4199(2)	2391(1)	27(1)
C(15)	9160(4)	4959(2)	2102(1)	29(1)
C(16)	7803(4)	5623(2)	2480(1)	26(1)
C(17)	7569(4)	5526(2)	3145(1)	24(1)
C(18)	8016(3)	3083(2)	6233(1)	20(1)
C(19)	8390(5)	3318(3)	7296(1)	35(1)
C(20)	10468(4)	1479(2)	5302(1)	22(1)

C(21)	10953(4)	306(2)	6124(1)	28(1)
S(4)	7839(1)	416(1)	10968(1)	21(1)
S(5)	7617(1)	-935(1)	9979(1)	20(1)
S(6)	6772(1)	1916(1)	8625(1)	23(1)
O(9)	9062(3)	-2963(1)	11212(1)	26(1)
O(10)	7528(3)	-2844(1)	10353(1)	24(1)
O(11)	8672(3)	-437(2)	12225(1)	32(1)
O(12)	7052(3)	-1588(1)	12165(1)	26(1)
O(13)	5790(3)	2754(2)	10848(1)	33(1)
O(14)	8878(3)	2568(1)	10528(1)	29(1)
O(15)	6937(3)	4113(1)	9702(1)	27(1)
O(16)	6159(3)	4008(1)	8712(1)	27(1)
N(2)	7158(3)	-53(2)	8904(1)	21(1)
C(22)	7554(3)	254(2)	10179(1)	19(1)
C(23)	7901(3)	-1408(2)	10752(1)	20(1)
C(24)	7932(3)	-776(2)	11214(1)	20(1)
C(25)	7114(3)	815(2)	9076(1)	19(1)
C(26)	7319(3)	1011(2)	9735(1)	19(1)
C(27)	7169(3)	2029(2)	9833(1)	19(1)
C(28)	6910(4)	2576(2)	9295(1)	21(1)
C(29)	8224(4)	-2484(2)	10820(1)	21(1)
C(30)	7973(4)	-3891(2)	10312(2)	31(1)
C(31)	7977(3)	-926(2)	11922(1)	21(1)
C(32)	6885(4)	-1708(2)	12857(1)	30(1)
C(33)	6992(3)	-383(2)	8299(1)	21(1)
C(34)	7202(4)	105(2)	7714(1)	26(1)
C(35)	6969(4)	-314(2)	7151(1)	31(1)
C(36)	6538(4)	-1219(2)	7161(1)	32(1)
C(37)	6359(4)	-1719(2)	7742(1)	31(1)
C(38)	6592(4)	-1308(2)	8303(1)	25(1)
C(39)	7162(4)	2496(2)	10462(1)	23(1)
C(40)	9058(5)	3061(3)	11094(2)	45(1)
C(41)	6698(4)	3636(2)	9270(1)	22(1)
C(42)	5805(5)	5060(2)	8666(2)	33(1)

Table S3. Bond lengths [Å] and angles [°] for compound 10a (No. CCDC 2205782).

S(1)-C(1)	1.728(2)
S(1)-C(3)	1.728(3)
S(2)-C(1)	1.745(2)
S(2)-C(2)	1.754(3)
S(3)-C(7)	1.750(2)
S(3)-C(4)	1.779(3)
O(1)-C(8)	1.195(3)
O(2)-C(8)	1.336(3)
O(2)-C(9)	1.449(3)
O(3)-C(10)	1.206(3)
O(4)-C(10)	1.320(3)
O(4)-C(11)	1.448(3)
O(5)-C(18)	1.200(3)
O(6)-C(18)	1.333(3)
O(6)-C(19)	1.453(3)
O(7)-C(20)	1.208(3)
O(8)-C(20)	1.336(3)
O(8)-C(21)	1.454(3)
N(1)-C(4)	1.282(3)
N(1)-C(12)	1.404(3)
C(1)-C(5)	1.388(3)
C(2)-C(3)	1.356(3)
C(2)-C(8)	1.497(3)
C(3)-C(10)	1.490(3)
C(4)-C(5)	1.461(3)
C(5)-C(6)	1.440(3)
C(6)-C(7)	1.363(3)
C(6)-C(18)	1.499(3)
C(7)-C(20)	1.466(4)
C(9)-H(9A)	0.9800
C(9)-H(9B)	0.9800
C(9)-H(9C)	0.9800
C(11)-H(11A)	0.9800
C(11)-H(11B)	0.9800
C(11)-H(11C)	0.9800
C(12)-C(13)	1.400(4)
C(12)-C(17)	1.404(4)
C(13)-C(14)	1.392(3)

C(13)-H(13)	0.9500
C(14)-C(15)	1.383(4)
C(14)-H(14)	0.9500
C(15)-C(16)	1.387(4)
C(15)-H(15)	0.9500
C(16)-C(17)	1.385(3)
C(16)-H(16)	0.9500
C(17)-H(17)	0.9500
C(19)-H(19A)	0.9800
C(19)-H(19B)	0.9800
C(19)-H(19C)	0.9800
C(21)-H(21A)	0.9800
C(21)-H(21B)	0.9800
C(21)-H(21C)	0.9800
S(4)-C(22)	1.732(2)
S(4)-C(24)	1.733(2)
S(5)-C(22)	1.738(2)
S(5)-C(23)	1.755(2)
S(6)-C(28)	1.747(3)
S(6)-C(25)	1.780(2)
O(9)-C(29)	1.202(3)
O(10)-C(29)	1.341(3)
O(10)-C(30)	1.446(3)
O(11)-C(31)	1.203(3)
O(12)-C(31)	1.327(3)
O(12)-C(32)	1.446(3)
O(13)-C(39)	1.201(3)
O(14)-C(39)	1.333(3)
O(14)-C(40)	1.439(3)
O(15)-C(41)	1.208(3)
O(16)-C(41)	1.345(3)
O(16)-C(42)	1.453(3)
N(2)-C(25)	1.284(3)
N(2)-C(33)	1.401(3)
C(22)-C(26)	1.390(3)
C(23)-C(24)	1.351(3)
C(23)-C(29)	1.490(3)
C(24)-C(31)	1.497(3)
C(25)-C(26)	1.457(3)
C(26)-C(27)	1.440(3)

C(27)-C(28)	1.356(3)
C(27)-C(39)	1.499(3)
C(28)-C(41)	1.471(3)
C(30)-H(30A)	0.9800
C(30)-H(30B)	0.9800
C(30)-H(30C)	0.9800
C(32)-H(32A)	0.9800
C(32)-H(32B)	0.9800
C(32)-H(32C)	0.9800
C(33)-C(34)	1.392(3)
C(33)-C(38)	1.406(3)
C(34)-C(35)	1.392(4)
C(34)-H(34)	0.9500
C(35)-C(36)	1.387(4)
C(35)-H(35)	0.9500
C(36)-C(37)	1.389(4)
C(36)-H(36)	0.9500
C(37)-C(38)	1.384(4)
C(37)-H(37)	0.9500
C(38)-H(38)	0.9500
C(40)-H(40A)	0.9800
C(40)-H(40B)	0.9800
C(40)-H(40C)	0.9800
C(42)-H(42A)	0.9800
C(42)-H(42B)	0.9800
C(42)-H(42C)	0.9800
C(1)-S(1)-C(3)	97.06(12)
C(1)-S(2)-C(2)	95.00(12)
C(7)-S(3)-C(4)	91.17(12)
C(8)-O(2)-C(9)	115.2(2)
C(10)-O(4)-C(11)	116.7(2)
C(18)-O(6)-C(19)	113.9(2)
C(20)-O(8)-C(21)	115.2(2)
C(4)-N(1)-C(12)	127.8(2)
C(5)-C(1)-S(1)	123.07(18)
C(5)-C(1)-S(2)	122.42(18)
S(1)-C(1)-S(2)	114.50(14)
C(3)-C(2)-C(8)	127.2(2)
C(3)-C(2)-S(2)	117.5(2)

C(8)-C(2)-S(2)	115.15(17)
C(2)-C(3)-C(10)	130.2(2)
C(2)-C(3)-S(1)	115.82(19)
C(10)-C(3)-S(1)	113.92(18)
N(1)-C(4)-C(5)	119.9(2)
N(1)-C(4)-S(3)	130.34(19)
C(5)-C(4)-S(3)	109.67(18)
C(1)-C(5)-C(6)	128.1(2)
C(1)-C(5)-C(4)	119.9(2)
C(6)-C(5)-C(4)	112.0(2)
C(7)-C(6)-C(5)	113.0(2)
C(7)-C(6)-C(18)	122.6(2)
C(5)-C(6)-C(18)	124.2(2)
C(6)-C(7)-C(20)	129.8(2)
C(6)-C(7)-S(3)	114.15(19)
C(20)-C(7)-S(3)	116.05(18)
O(1)-C(8)-O(2)	125.6(2)
O(1)-C(8)-C(2)	126.0(2)
O(2)-C(8)-C(2)	108.2(2)
O(2)-C(9)-H(9A)	109.5
O(2)-C(9)-H(9B)	109.5
H(9A)-C(9)-H(9B)	109.5
O(2)-C(9)-H(9C)	109.5
H(9A)-C(9)-H(9C)	109.5
H(9B)-C(9)-H(9C)	109.5
O(3)-C(10)-O(4)	126.0(2)
O(3)-C(10)-C(3)	121.5(2)
O(4)-C(10)-C(3)	112.5(2)
O(4)-C(11)-H(11A)	109.5
O(4)-C(11)-H(11B)	109.5
H(11A)-C(11)-H(11B)	109.5
O(4)-C(11)-H(11C)	109.5
H(11A)-C(11)-H(11C)	109.5
H(11B)-C(11)-H(11C)	109.5
C(13)-C(12)-N(1)	126.0(2)
C(13)-C(12)-C(17)	119.0(2)
N(1)-C(12)-C(17)	115.0(2)
C(14)-C(13)-C(12)	119.6(2)
C(14)-C(13)-H(13)	120.2
C(12)-C(13)-H(13)	120.2

C(15)-C(14)-C(13)	121.0(3)
C(15)-C(14)-H(14)	119.5
C(13)-C(14)-H(14)	119.5
C(14)-C(15)-C(16)	119.7(2)
C(14)-C(15)-H(15)	120.1
C(16)-C(15)-H(15)	120.1
C(17)-C(16)-C(15)	120.1(3)
C(17)-C(16)-H(16)	119.9
C(15)-C(16)-H(16)	119.9
C(16)-C(17)-C(12)	120.6(3)
C(16)-C(17)-H(17)	119.7
C(12)-C(17)-H(17)	119.7
O(5)-C(18)-O(6)	125.0(2)
O(5)-C(18)-C(6)	123.0(2)
O(6)-C(18)-C(6)	111.9(2)
O(6)-C(19)-H(19A)	109.5
O(6)-C(19)-H(19B)	109.5
H(19A)-C(19)-H(19B)	109.5
O(6)-C(19)-H(19C)	109.5
H(19A)-C(19)-H(19C)	109.5
H(19B)-C(19)-H(19C)	109.5
O(7)-C(20)-O(8)	124.6(2)
O(7)-C(20)-C(7)	122.8(2)
O(8)-C(20)-C(7)	112.6(2)
O(8)-C(21)-H(21A)	109.5
O(8)-C(21)-H(21B)	109.5
H(21A)-C(21)-H(21B)	109.5
O(8)-C(21)-H(21C)	109.5
H(21A)-C(21)-H(21C)	109.5
H(21B)-C(21)-H(21C)	109.5
C(22)-S(4)-C(24)	96.44(12)
C(22)-S(5)-C(23)	95.12(11)
C(28)-S(6)-C(25)	91.09(12)
C(29)-O(10)-C(30)	115.1(2)
C(31)-O(12)-C(32)	115.3(2)
C(39)-O(14)-C(40)	116.3(2)
C(41)-O(16)-C(42)	114.7(2)
C(25)-N(2)-C(33)	128.7(2)
C(26)-C(22)-S(4)	122.89(18)
C(26)-C(22)-S(5)	122.33(18)

S(4)-C(22)-S(5)	114.78(13)
C(24)-C(23)-C(29)	126.4(2)
C(24)-C(23)-S(5)	117.27(18)
C(29)-C(23)-S(5)	116.21(18)
C(23)-C(24)-C(31)	130.4(2)
C(23)-C(24)-S(4)	116.21(18)
C(31)-C(24)-S(4)	113.37(17)
N(2)-C(25)-C(26)	120.4(2)
N(2)-C(25)-S(6)	129.79(19)
C(26)-C(25)-S(6)	109.83(17)
C(22)-C(26)-C(27)	128.8(2)
C(22)-C(26)-C(25)	119.7(2)
C(27)-C(26)-C(25)	111.5(2)
C(28)-C(27)-C(26)	113.5(2)
C(28)-C(27)-C(39)	120.6(2)
C(26)-C(27)-C(39)	125.8(2)
C(27)-C(28)-C(41)	124.1(2)
C(27)-C(28)-S(6)	114.04(19)
C(41)-C(28)-S(6)	121.85(19)
O(9)-C(29)-O(10)	124.9(2)
O(9)-C(29)-C(23)	125.4(2)
O(10)-C(29)-C(23)	109.6(2)
O(10)-C(30)-H(30A)	109.5
O(10)-C(30)-H(30B)	109.5
H(30A)-C(30)-H(30B)	109.5
O(10)-C(30)-H(30C)	109.5
H(30A)-C(30)-H(30C)	109.5
H(30B)-C(30)-H(30C)	109.5
O(11)-C(31)-O(12)	125.9(2)
O(11)-C(31)-C(24)	121.7(2)
O(12)-C(31)-C(24)	112.2(2)
O(12)-C(32)-H(32A)	109.5
O(12)-C(32)-H(32B)	109.5
H(32A)-C(32)-H(32B)	109.5
O(12)-C(32)-H(32C)	109.5
H(32A)-C(32)-H(32C)	109.5
H(32B)-C(32)-H(32C)	109.5
C(34)-C(33)-N(2)	126.7(2)
C(34)-C(33)-C(38)	118.5(2)
N(2)-C(33)-C(38)	114.8(2)

C(33)-C(34)-C(35)	120.0(3)
C(33)-C(34)-H(34)	120.0
C(35)-C(34)-H(34)	120.0
C(36)-C(35)-C(34)	121.0(3)
C(36)-C(35)-H(35)	119.5
C(34)-C(35)-H(35)	119.5
C(35)-C(36)-C(37)	119.4(2)
C(35)-C(36)-H(36)	120.3
C(37)-C(36)-H(36)	120.3
C(38)-C(37)-C(36)	119.9(3)
C(38)-C(37)-H(37)	120.1
C(36)-C(37)-H(37)	120.1
C(37)-C(38)-C(33)	121.1(3)
C(37)-C(38)-H(38)	119.4
C(33)-C(38)-H(38)	119.4
O(13)-C(39)-O(14)	125.9(2)
O(13)-C(39)-C(27)	123.8(2)
O(14)-C(39)-C(27)	110.3(2)
O(14)-C(40)-H(40A)	109.5
O(14)-C(40)-H(40B)	109.5
H(40A)-C(40)-H(40B)	109.5
O(14)-C(40)-H(40C)	109.5
H(40A)-C(40)-H(40C)	109.5
H(40B)-C(40)-H(40C)	109.5
O(15)-C(41)-O(16)	124.3(2)
O(15)-C(41)-C(28)	124.3(2)
O(16)-C(41)-C(28)	111.4(2)
O(16)-C(42)-H(42A)	109.5
O(16)-C(42)-H(42B)	109.5
H(42A)-C(42)-H(42B)	109.5
O(16)-C(42)-H(42C)	109.5
H(42A)-C(42)-H(42C)	109.5
H(42B)-C(42)-H(42C)	109.5

Table S4. Anisotropic displacement parameters (Å^{2}x 10^3) for compound 10a (No. CCDC 2205782). The anisotropic displacement factor exponent takes the form: $-2\pi^2$[h^2 $a^{*2}U^{11}$ + 2 h k a* b* U^{12}]

	U^{11}	U^{22}	U^{33}	U^{23}	U^{13}	U^{12}
S(1)	27(1)	18(1)	19(1)	2(1)	-3(1)	-6(1)
S(2)	25(1)	18(1)	18(1)	2(1)	-4(1)	-7(1)
S(3)	26(1)	20(1)	19(1)	1(1)	-3(1)	-6(1)
O(1)	39(1)	21(1)	25(1)	1(1)	-9(1)	-7(1)
O(2)	39(1)	20(1)	20(1)	2(1)	-8(1)	-4(1)
O(3)	38(1)	26(1)	20(1)	1(1)	-6(1)	-8(1)
O(4)	28(1)	26(1)	24(1)	2(1)	-1(1)	-2(1)
O(5)	30(1)	31(1)	23(1)	1(1)	-2(1)	-15(1)
O(6)	31(1)	29(1)	17(1)	1(1)	-4(1)	-15(1)
O(7)	45(1)	23(1)	24(1)	1(1)	0(1)	-3(1)
O(8)	36(1)	21(1)	21(1)	4(1)	-5(1)	-4(1)
N(1)	24(1)	20(1)	18(1)	2(1)	-3(1)	-6(1)
C(1)	24(1)	18(1)	19(1)	2(1)	-5(1)	-8(1)
C(2)	22(1)	20(1)	21(1)	-1(1)	-5(1)	-7(1)
C(3)	21(1)	20(1)	20(1)	0(1)	-4(1)	-6(1)
C(4)	19(1)	19(1)	22(1)	-1(1)	-3(1)	-7(1)
C(5)	21(1)	18(1)	20(1)	1(1)	-4(1)	-7(1)
C(6)	22(1)	20(1)	19(1)	2(1)	-4(1)	-8(1)
C(7)	23(1)	21(1)	18(1)	3(1)	-4(1)	-7(1)
C(8)	22(1)	20(1)	20(1)	1(1)	-3(1)	-5(1)
C(9)	44(2)	22(1)	25(1)	5(1)	-6(1)	-3(1)
C(10)	25(1)	21(1)	21(1)	-2(1)	-4(1)	-11(1)
C(11)	32(2)	27(1)	30(1)	-3(1)	4(1)	-2(1)
C(12)	24(1)	20(1)	19(1)	3(1)	-4(1)	-11(1)
C(13)	30(1)	22(1)	21(1)	2(1)	-3(1)	-9(1)
C(14)	35(1)	25(1)	21(1)	0(1)	-1(1)	-9(1)
C(15)	41(2)	28(1)	20(1)	4(1)	-6(1)	-15(1)
C(16)	33(1)	25(1)	23(1)	8(1)	-9(1)	-12(1)
C(17)	27(1)	22(1)	24(1)	5(1)	-4(1)	-11(1)
C(18)	24(1)	15(1)	21(1)	2(1)	-4(1)	-5(1)
C(19)	43(2)	48(2)	17(1)	3(1)	-4(1)	-21(1)
C(20)	25(1)	21(1)	22(1)	2(1)	-2(1)	-7(1)

C(21)	38(2)	20(1)	27(1)	4(1)	-7(1)	-7(1)
S(4)	29(1)	18(1)	18(1)	1(1)	-3(1)	-9(1)
S(5)	28(1)	16(1)	18(1)	2(1)	-4(1)	-8(1)
S(6)	32(1)	19(1)	20(1)	4(1)	-5(1)	-10(1)
O(9)	31(1)	23(1)	26(1)	6(1)	-6(1)	-7(1)
O(10)	32(1)	18(1)	25(1)	0(1)	-5(1)	-10(1)
O(11)	43(1)	40(1)	21(1)	3(1)	-8(1)	-24(1)
O(12)	37(1)	26(1)	17(1)	5(1)	-3(1)	-16(1)
O(13)	45(1)	25(1)	28(1)	-4(1)	9(1)	-15(1)
O(14)	36(1)	26(1)	28(1)	-3(1)	-12(1)	-5(1)
O(15)	37(1)	21(1)	25(1)	1(1)	-4(1)	-10(1)
O(16)	39(1)	18(1)	24(1)	6(1)	-5(1)	-7(1)
N(2)	27(1)	19(1)	19(1)	1(1)	-4(1)	-11(1)
C(22)	24(1)	17(1)	17(1)	1(1)	-3(1)	-7(1)
C(23)	23(1)	19(1)	19(1)	5(1)	-4(1)	-9(1)
C(24)	24(1)	19(1)	19(1)	3(1)	-3(1)	-7(1)
C(25)	22(1)	18(1)	19(1)	6(1)	-3(1)	-8(1)
C(26)	21(1)	16(1)	19(1)	2(1)	-2(1)	-6(1)
C(27)	21(1)	17(1)	21(1)	2(1)	-3(1)	-6(1)
C(28)	26(1)	19(1)	20(1)	1(1)	-2(1)	-8(1)
C(29)	24(1)	19(1)	22(1)	3(1)	0(1)	-9(1)
C(30)	42(2)	19(1)	36(2)	-2(1)	-5(1)	-13(1)
C(31)	24(1)	20(1)	20(1)	3(1)	-2(1)	-6(1)
C(32)	39(2)	32(2)	19(1)	7(1)	-1(1)	-12(1)
C(33)	22(1)	21(1)	20(1)	0(1)	-4(1)	-7(1)
C(34)	30(1)	28(1)	21(1)	2(1)	-5(1)	-9(1)
C(35)	30(1)	40(2)	21(1)	2(1)	-6(1)	-5(1)
C(36)	31(1)	40(2)	29(1)	-9(1)	-11(1)	-7(1)
C(37)	31(1)	31(2)	34(1)	-7(1)	-6(1)	-11(1)
C(38)	25(1)	25(1)	27(1)	-3(1)	-4(1)	-8(1)
C(39)	34(1)	16(1)	20(1)	3(1)	-4(1)	-8(1)
C(40)	63(2)	51(2)	28(2)	-5(1)	-17(2)	-22(2)
C(41)	26(1)	19(1)	22(1)	5(1)	-1(1)	-6(1)
C(42)	43(2)	19(1)	35(2)	9(1)	-5(1)	-5(1)

_

Table S5. Hydrogen coordinates (x 10^4) and isotropic displacement parameters ($Å^2$x 10^3) for compound 10a (No. CCDC 2205782).

	x	y	z	U(eq)
H(9A)	3915	8874	4094	46
H(9B)	5159	9220	4558	46
H(9C)	2999	9272	4799	46
H(11A)	896	8693	7160	47
H(11B)	2547	8255	7568	47
H(11C)	987	7663	7511	47
H(13)	10817	3554	3251	29
H(14)	11213	3748	2131	32
H(15)	9321	5026	1646	34
H(16)	7033	6146	2283	31
H(17)	6645	5987	3401	29
H(19A)	9243	3524	7546	52
H(19B)	8312	2649	7414	52
H(19C)	7152	3747	7389	52
H(21A)	10723	247	6595	42
H(21B)	12292	107	5975	42
H(21C)	10301	-112	5930	42
H(30A)	7431	-4089	9955	47
H(30B)	7461	-4165	10716	47
H(30C)	9327	-4130	10236	47
H(32A)	5992	-2113	12999	45
H(32B)	6448	-1071	13063	45
H(32C)	8102	-2021	12977	45
H(34)	7505	723	7698	31
H(35)	7107	25	6753	37
H(36)	6367	-1494	6775	39
H(37)	6077	-2342	7753	37
H(38)	6479	-1657	8698	30
H(40A)	10370	2926	11155	67
H(40B)	8328	2831	11471	67
H(40C)	8599	3762	11042	67
H(42A)	5324	5273	8262	50
H(42B)	6966	5270	8674	50
H(42C)	4886	5345	9031	50

Table S6. Torsion angles [°] for compound 10a (No. CCDC 2205782).

C(3)-S(1)-C(1)-C(5)	-177.8(2)
C(3)-S(1)-C(1)-S(2)	1.13(16)
C(2)-S(2)-C(1)-C(5)	179.7(2)
C(2)-S(2)-C(1)-S(1)	0.70(15)
C(1)-S(2)-C(2)-C(3)	-3.0(2)
C(1)-S(2)-C(2)-C(8)	172.24(18)
C(8)-C(2)-C(3)-C(10)	11.7(4)
S(2)-C(2)-C(3)-C(10)	-173.7(2)
C(8)-C(2)-C(3)-S(1)	-170.3(2)
S(2)-C(2)-C(3)-S(1)	4.4(3)
C(1)-S(1)-C(3)-C(2)	-3.3(2)
C(1)-S(1)-C(3)-C(10)	175.13(18)
C(12)-N(1)-C(4)-C(5)	-176.8(2)
C(12)-N(1)-C(4)-S(3)	0.2(4)
C(7)-S(3)-C(4)-N(1)	-175.9(2)
C(7)-S(3)-C(4)-C(5)	1.25(18)
S(1)-C(1)-C(5)-C(6)	0.5(4)
S(2)-C(1)-C(5)-C(6)	-178.37(19)
S(1)-C(1)-C(5)-C(4)	177.47(17)
S(2)-C(1)-C(5)-C(4)	-1.4(3)
N(1)-C(4)-C(5)-C(1)	-2.8(3)
S(3)-C(4)-C(5)-C(1)	179.73(18)
N(1)-C(4)-C(5)-C(6)	174.6(2)
S(3)-C(4)-C(5)-C(6)	-2.8(2)
C(1)-C(5)-C(6)-C(7)	-179.4(2)
C(4)-C(5)-C(6)-C(7)	3.5(3)
C(1)-C(5)-C(6)-C(18)	6.5(4)
C(4)-C(5)-C(6)-C(18)	-170.7(2)
C(5)-C(6)-C(7)-C(20)	179.2(2)
C(18)-C(6)-C(7)-C(20)	-6.6(4)
C(5)-C(6)-C(7)-S(3)	-2.5(3)
C(18)-C(6)-C(7)-S(3)	171.70(18)
C(4)-S(3)-C(7)-C(6)	0.7(2)
C(4)-S(3)-C(7)-C(20)	179.26(19)
C(9)-O(2)-C(8)-O(1)	-1.0(4)
C(9)-O(2)-C(8)-C(2)	-177.0(2)
C(3)-C(2)-C(8)-O(1)	29.2(4)
S(2)-C(2)-C(8)-O(1)	-145.5(2)

C(3)-C(2)-C(8)-O(2)	-154.9(2)
S(2)-C(2)-C(8)-O(2)	30.4(3)
C(11)-O(4)-C(10)-O(3)	2.9(4)
C(11)-O(4)-C(10)-C(3)	-179.3(2)
C(2)-C(3)-C(10)-O(3)	-161.6(3)
S(1)-C(3)-C(10)-O(3)	20.3(3)
C(2)-C(3)-C(10)-O(4)	20.5(4)
S(1)-C(3)-C(10)-O(4)	-157.58(17)
C(4)-N(1)-C(12)-C(13)	-19.1(4)
C(4)-N(1)-C(12)-C(17)	161.8(2)
N(1)-C(12)-C(13)-C(14)	-179.0(2)
C(17)-C(12)-C(13)-C(14)	0.1(4)
C(12)-C(13)-C(14)-C(15)	-0.7(4)
C(13)-C(14)-C(15)-C(16)	0.6(4)
C(14)-C(15)-C(16)-C(17)	0.0(4)
C(15)-C(16)-C(17)-C(12)	-0.6(4)
C(13)-C(12)-C(17)-C(16)	0.5(4)
N(1)-C(12)-C(17)-C(16)	179.7(2)
C(19)-O(6)-C(18)-O(5)	-1.8(4)
C(19)-O(6)-C(18)-C(6)	174.7(2)
C(7)-C(6)-C(18)-O(5)	-81.7(3)
C(5)-C(6)-C(18)-O(5)	91.9(3)
C(7)-C(6)-C(18)-O(6)	101.7(3)
C(5)-C(6)-C(18)-O(6)	-84.7(3)
C(21)-O(8)-C(20)-O(7)	-2.7(4)
C(21)-O(8)-C(20)-C(7)	177.4(2)
C(6)-C(7)-C(20)-O(7)	173.7(3)
S(3)-C(7)-C(20)-O(7)	-4.6(3)
C(6)-C(7)-C(20)-O(8)	-6.5(4)
S(3)-C(7)-C(20)-O(8)	175.27(17)
C(24)-S(4)-C(22)-C(26)	177.5(2)
C(24)-S(4)-C(22)-S(5)	-3.34(16)
C(23)-S(5)-C(22)-C(26)	-179.3(2)
C(23)-S(5)-C(22)-S(4)	1.64(16)
C(22)-S(5)-C(23)-C(24)	1.4(2)
C(22)-S(5)-C(23)-C(29)	-174.19(19)
C(29)-C(23)-C(24)-C(31)	-11.1(4)
S(5)-C(23)-C(24)-C(31)	173.8(2)
C(29)-C(23)-C(24)-S(4)	171.1(2)
S(5)-C(23)-C(24)-S(4)	-4.0(3)

C(22)-S(4)-C(24)-C(23)	4.4(2)
C(22)-S(4)-C(24)-C(31)	-173.78(18)
C(33)-N(2)-C(25)-C(26)	-179.8(2)
C(33)-N(2)-C(25)-S(6)	1.8(4)
C(28)-S(6)-C(25)-N(2)	178.6(3)
C(28)-S(6)-C(25)-C(26)	0.11(19)
S(4)-C(22)-C(26)-C(27)	-3.5(4)
S(5)-C(22)-C(26)-C(27)	177.5(2)
S(4)-C(22)-C(26)-C(25)	179.10(18)
S(5)-C(22)-C(26)-C(25)	0.1(3)
N(2)-C(25)-C(26)-C(22)	-0.3(4)
S(6)-C(25)-C(26)-C(22)	178.38(19)
N(2)-C(25)-C(26)-C(27)	-178.1(2)
S(6)-C(25)-C(26)-C(27)	0.6(3)
C(22)-C(26)-C(27)-C(28)	-178.8(2)
C(25)-C(26)-C(27)-C(28)	-1.2(3)
C(22)-C(26)-C(27)-C(39)	-2.6(4)
C(25)-C(26)-C(27)-C(39)	174.9(2)
C(26)-C(27)-C(28)-C(41)	179.4(2)
C(39)-C(27)-C(28)-C(41)	3.0(4)
C(26)-C(27)-C(28)-S(6)	1.3(3)
C(39)-C(27)-C(28)-S(6)	-175.03(19)
C(25)-S(6)-C(28)-C(27)	-0.8(2)
C(25)-S(6)-C(28)-C(41)	-178.9(2)
C(30)-O(10)-C(29)-O(9)	-4.5(4)
C(30)-O(10)-C(29)-C(23)	172.0(2)
C(24)-C(23)-C(29)-O(9)	-22.8(4)
S(5)-C(23)-C(29)-O(9)	152.4(2)
C(24)-C(23)-C(29)-O(10)	160.7(2)
S(5)-C(23)-C(29)-O(10)	-24.1(3)
C(32)-O(12)-C(31)-O(11)	0.4(4)
C(32)-O(12)-C(31)-C(24)	-174.4(2)
C(23)-C(24)-C(31)-O(11)	151.8(3)
S(4)-C(24)-C(31)-O(11)	-30.3(3)
C(23)-C(24)-C(31)-O(12)	-33.1(4)
S(4)-C(24)-C(31)-O(12)	144.80(19)
C(25)-N(2)-C(33)-C(34)	17.2(4)
C(25)-N(2)-C(33)-C(38)	-163.5(3)
N(2)-C(33)-C(34)-C(35)	-179.1(3)
C(38)-C(33)-C(34)-C(35)	1.6(4)

C(33)-C(34)-C(35)-C(36)	-0.4(4)
C(34)-C(35)-C(36)-C(37)	-0.8(4)
C(35)-C(36)-C(37)-C(38)	0.7(4)
C(36)-C(37)-C(38)-C(33)	0.6(4)
C(34)-C(33)-C(38)-C(37)	-1.8(4)
N(2)-C(33)-C(38)-C(37)	178.9(2)
C(40)-O(14)-C(39)-O(13)	-4.5(4)
C(40)-O(14)-C(39)-C(27)	176.2(2)
C(28)-C(27)-C(39)-O(13)	90.6(3)
C(26)-C(27)-C(39)-O(13)	-85.3(3)
C(28)-C(27)-C(39)-O(14)	-90.1(3)
C(26)-C(27)-C(39)-O(14)	94.0(3)
C(42)-O(16)-C(41)-O(15)	-2.2(4)
C(42)-O(16)-C(41)-C(28)	176.3(2)
C(27)-C(28)-C(41)-O(15)	8.7(4)
S(6)-C(28)-C(41)-O(15)	-173.4(2)
C(27)-C(28)-C(41)-O(16)	-169.9(2)
S(6)-C(28)-C(41)-O(16)	8.1(3)
